# Three Is Better than One: A Multimetal Complex that Triggers Immunogenic Cell Death

**DOI:** 10.1002/anie.202514351

**Published:** 2025-08-12

**Authors:** Tomer Babu, Matthew S. Levine, Sourav Acharya, Esther Y. Maier, Jonathan L. Sessler

**Affiliations:** ^1^ Department of Chemistry The University of Texas at Austin 105 East 24th Street Austin Texas 78712‐1224 USA; ^2^ Institute for Drug Research School of Pharmacy The Hebrew University of Jerusalem Jerusalem 9112102 Israel; ^3^ Innovation for Health Institute College of Pharmacy The University of Texas at Austin 1400 Barbara Jordan Blvd Austin Texas 78723 USA

**Keywords:** Gold(I) N‐heterocyclic carbenes, Immunogenic cell death, Metals in medicine, Platinum(IV) prodrugs, Ruthenium(II) 2‐pyridinecarbothioamides

## Abstract

Immunogenic cell death (ICD) is a form of regulated cell death capable of stimulating an adaptive immune response through release of damage‐associated molecular patterns (DAMPs). While several metal complexes have shown promise as ICD inducers, conjugates containing more than one metal center remain to be explored for ICD. Here, we report the design, synthesis, and evaluation of a tri‐metallic Au^I^‐Pt^IV^‐Ru^II^ prodrug (**5**) that codelivers oxaliplatin (Type I ICD inducer), Ru^II^ arene 2‐pyridinecarbothioamides (Type I), and Au(I) bis‐N‐heterocyclic (Type II) using a single scaffold. Upon reduction, complex **5** releases all three cytotoxic species, resulting in enhanced inhibition of thioredoxin reductase (TrxR1&2), elevated production of reactive oxygen species (ROS), and in vitro DAMP release. In vivo studies of complex **5** in a colorectal cancer mouse model demonstrated tumor growth suppression, reduced off‐target metal accumulation, and improved immune memory in a tumor challenge study compared to a 1:1:1 mixture of the constituent agents. Furthermore, peripheral white blood cell (WBC) profiling revealed that complex **5** activates both the innate and adaptive immune compartments. This study demonstrates an integrated chemo‐immunotherapeutic strategy that unifies multiple ICD triggers within a single prodrug framework. It thus highlights a promising approach to the creation of metal‐based multimodal anticancer therapies.

## Graphical abstract

A triple metal prodrug that codelivers Pt^II^, Ru^II^, and Au^I^ species triggers a potent immunogenic cell death (ICD) response via combined Type I/II pathways. The tri‐metallic scaffold promotes tumor inhibition, immune memory, and peripheral leukocyte activation, offering a next‐generation chemo‐immunotherapeutic approach (created in BioRender. Babu, T., 2025).

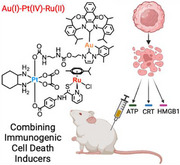

## Introduction

**Figure 1:**
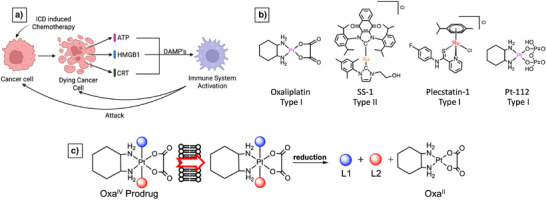
a) General mechanism of action (MOA) of ICD induced chemotherapeutics. b) Examples of type I and type II metal‐based ICD inducers. c) Schematic illustration of Pt(IV) oxaliplatin intracellular reduction, releasing both axial ligands (L1 and L2) and the FDA approved drug Oxa.

Immunogenic cell death (ICD) represents a unique strategy in oncology, converting dying cancer cells into a vaccine‐like stimulus that elicits durable antitumor immunity. During ICD, tumor cells release damage‐associated molecular patterns (DAMPs), such as calreticulin (CRT), adenosine triphosphate (ATP), and high mobility group box 1 protein (HMGB1), that promote immune system activation (Figure 1a), effectively transforming dying tumor cells into a source of in situ immunization.^[^ ^1^, ^2^ ^]^ In recent years, metal complexes have emerged as potent and versatile ICD inducers, capable of engaging diverse cellular stress pathways while offering chemical modularity and tunable pharmacology.^[^ ^3^, ^4^ ^]^

ICD inducers are broadly categorized into two types. Type I inducers, such as oxaliplatin (Oxa)^[^ ^5^, ^6^ ^]^ and reports of potential ICD activity by Ru^II^ arene 2‐pyridinecarbothioamides,^[^ ^7^ ^]^ that elicit ICD by targeting non‐ER structures, such as DNA or mitochondria, with endoplasmic reticulum (ER) stress arising as a downstream consequence. In contrast, Type II inducers, which include Ir^III^ N‐heterocyclic^[^ ^8^ ^]^ (NHC) and Au^I^ complexes,^[^ ^9^, ^10^ ^]^ directly engage the ER, typically through the localized production of reactive oxygen species (ROS), to promote immunogenic signaling (Figure 1b). While several gold NHC complexes have been reported to trigger DAMPs release, they may also exert immunosuppressive effects depending on the ligands that are coordinated to the metal center.^[^ ^11^ ^]^

**Figure 2:**
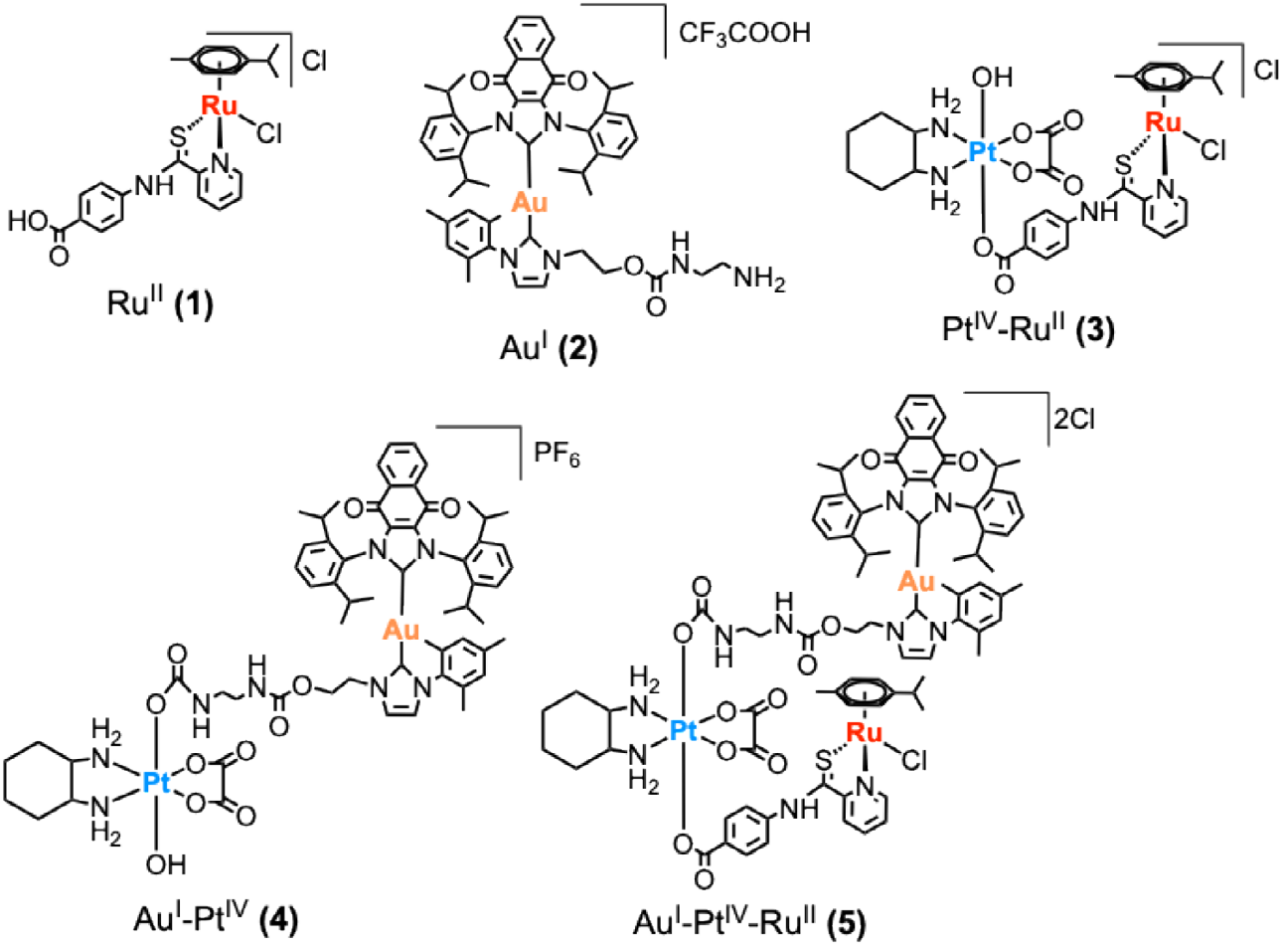
Chemical structures of newly synthesized mono, bi‐ & tri‐metallic complexes used in this study.

Chemotherapeutics that can induce ICD are attractive in that they can both inhibit primary tumors and promote a systemic immune response that suppresses distant or metastatic tumors.^[^ ^2^ ^]^ This outcome can potentially be increased by combining ICD inducers with other chemotherapeutic treatments. However, achieving this in practice is not necessarily straightforward. In spite of its widespread use, the coadministration of multiple drugs faces practical and pharmacological challenges.^[^ ^12^ ^]^ Differences in solubility, circulation half‐lives, cellular uptake, and metabolism often lead to asynchronous delivery, unpredictable local concentrations, and diminished synergy. Moreover, drug mixtures can result in increased off‐target toxicity or antagonistic interactions.^[^ ^13^ ^]^ One approach to avoid these drawbacks involves integrating multiple drugs into a single prodrug scaffold that can release several moieties simultaneously upon activation.^[^ ^14^ ^]^ Pt^IV^ scaffolds offer several advantages in this context (Figure 1c): They are kinetically inert under physiological conditions, chemically versatile, and selectively activated in tumor cells and in the tumor microenvironment (TME).^[^ ^15^ ^]^ This activation comes in the form of reduction to the corresponding Pt^II^ species by endogenous reductants, such as ascorbate, glutathione (GSH), or low molecular weight antioxidents.^[^ ^16^, ^17^ ^]^ This leads to loss of the axial ligands and conversion from a six‐coordinate pseudo‐octahedral complex to a square planar four‐coordinate species. If the axial ligands comprise a drug, this provides a mechanism for targeted drug release. The viability of this approach was demonstrated by Gibson et al. who showed that Pt^IV^ prodrugs containing two cytotoxic heavy metals displayed low systemic toxicity.^[^ ^18^ ^]^ Furthermore, a conjugate linking doxorubicin to Oxa^IV^, a Pt^IV^ analogue of Oxa, exhibited superior tumor inhibition and enhanced immunogenicity in vivo compared to the coadministration of the constituent species.^[^ ^19^ ^]^ Rationally combining Type I and Type II ICD stimuli within such construct presents an opportunity to intensify and diversify the immunogenic response shown for stand‐alone ICD agents while addressing key issues associated with coadministration of multiple drugs. Herein, we report the synthesis, characterization, and biological evaluation of a series of bi‐, and tri‐metallic complexes **3**–**5** (Figure 2) that contain Ru^II^‐ and Au^I^‐based ICD inducers linked to Oxa^IV^. These conjugates were designed to codeliver Type I and Type II ICD stimuli, respectively, upon reduction of Oxa^IV^ to Oxa. The tri‐metallic conjugate **5**, in particular, demonstrated in vitro cytotoxicity, potent DAMP release, in vivo tumor inhibition, and vaccination model efficacy, outperforming both the individual agents and their physical mixtures. To our knowledge, this is the first report on combining distinct metal complexes, where each is an ICD inducer.

## Results and Discussion

### Synthesis and Design of Mono‐, Bi‐, and Tri‐Metallic Complexes 3–5

Complexes **3**, **4**, and **5** were designed and synthesized by derivatizing Ru^II^ and Au^I^ scaffolds previously shown to modulate plectin and inhibit TrxR enzymes, as well as displaying features consistent with an ICD response in cancer cells (Figure 2).^[^ ^7^, ^9^ ^]^ Complex **1** contains a carboxylic acid functional group suitable for conjugation to the axial position of an Oxa^IV^ scaffold, while complex **2** bears a primary amine, enabling linkage through a recently reported carbamate formation strategy.^[^ ^20^ ^]^

**Scheme 1:**
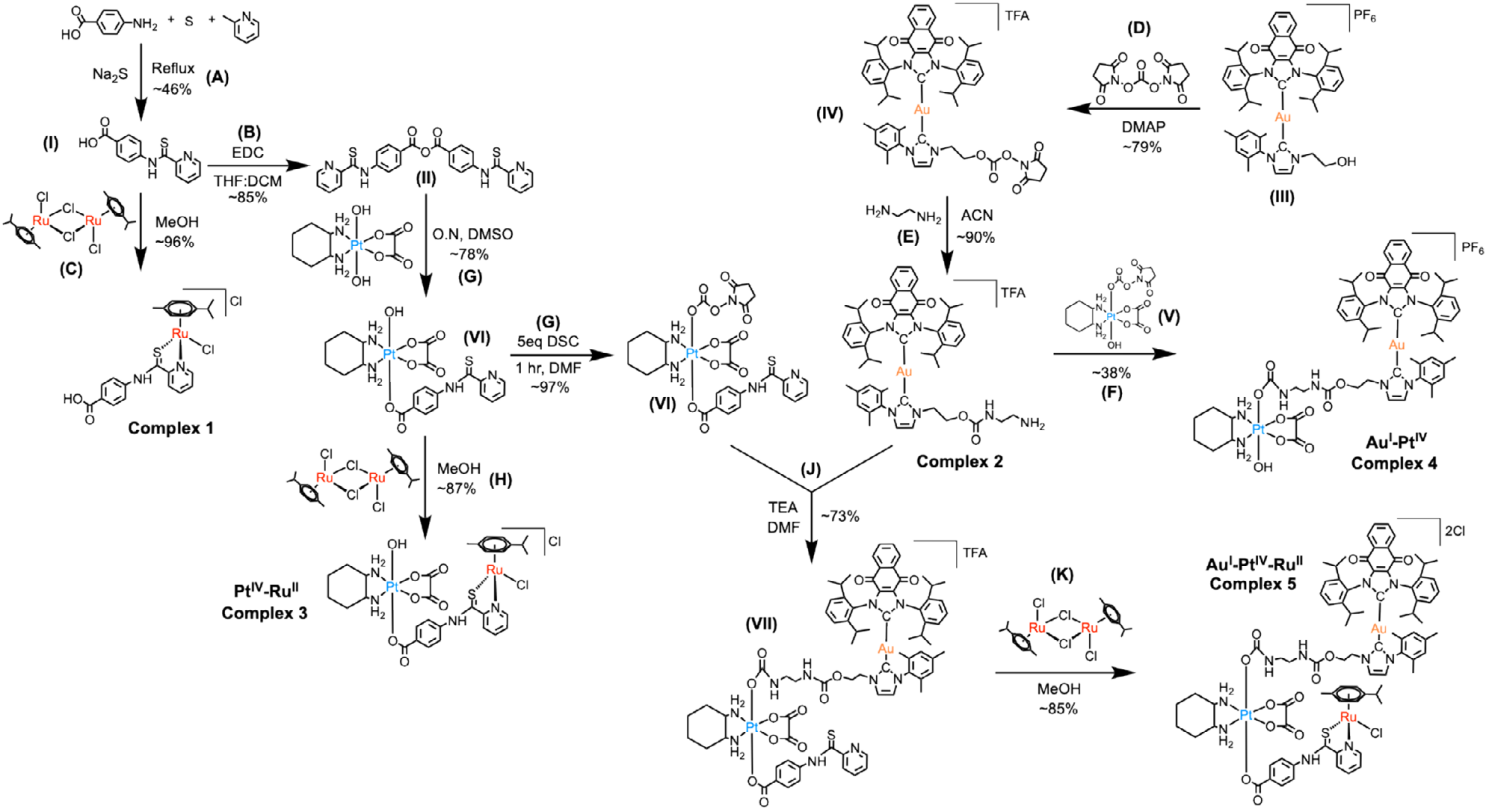
Synthetic routes to the bi‐ & tri‐metal complexes of this study. Reaction conditions: A) 2‐Picoline, 135 °C, overnight (O.N.); B) 0.3 h, room temperature (r.t); C) pH 6.6, 0.25 h, r.t.; D) acetonitrile (ACN), 1 h, r.t.; E) ACN, 0.16 h, r.t.; F) ACN / DMF, 3 h, r.t.; G) DMSO, 40 °C, O.N.; H) MeOH, 0.16 h, r.t.; J) DMF, 1 h, r.t.; K) MeOH, 0.16 h, r.t. (see Supporting Information for detailed synthesis description).

Using this modular approach, bi‐metallic complexes **3** and **4** were generated by coupling either complex **1** (Ru^II^) or complex **2** (Au^I^) to Oxa^IV^, while tri‐metallic complex **5** incorporated both metals in a single complex; it was prepared in accord with the synthetic strategy depicted in Scheme 1. The approach used to access complex **5** differs from the coupling strategies typically used to prepared hetero‐metallic complexes. Generally, separate metal complexes are prepared and then linked to one another as the final step. In case of the Ru^II^ precursor, complex **1**, we were unable to link it directly to the axial position of Oxa^IV^. Thus, as an alternative, we first coupled the pyridinecarbothioamide ligand via anhydride preparation with subsequent addition of [Ru(η6‐p‐cymene)Cl_2_]_2_ to generate either complex **3** or **5**. All newly synthesized mono‐, bi‐, and tri‐metallic complexes were obtained in good yield and high purity. They were fully characterized by HPLC, NMR spectroscopy, HRMS, and elemental analysis (see Supporting Information).

### Physiochemical Properties

The physicochemical properties of complexes **3**–**5** were assessed with the goal of evaluating their propensity to undergo reduction, lipophilicity, and extracellular stability under physiological conditions. All three complexes were subjected to stability assessment in RPMI1640 medium at 37 °C, monitored by HPLC. This analysis revealed distinct degradation profiles (Figure S30), with complex **5** displaying the highest stability (*t*_1/2_ = 115.5 h), followed by complexes **4** (*t*_1/2 _= 89 h) and **3** (*t*_1/2 _= 5.8 h). The ability of complexes **3**–**5** to undergo reduction in the presence of a 10‐fold excess of ascorbate at pH 7.0, 37 °C was also assessed by HPLC. All three complexes were found to be readily reduced under these conditions with half‐lives of less than an hour (Table 1).

**Table 1:** Half‐lives of complexes **3**–**5** in RPMI1640 medium determined in the presence of excess ascorbic acid and their respective logP values (tested in triplicate).

**Complex**	**Reduction t1/2 (h)**	**Stability t1/2 (h)**	**logP**
3	0.24	5.8	−0.81 ± 0.02
4	0.08	89	1.73 ± 0.36
5	0.4	115.5	1.05 ± 0.01

To complement these data, we examined the lipophilicity of complexes **3**–**5**, a key factor influencing membrane permeability and cellular uptake.^[^ ^21^ ^]^ The logP values of the new heterometallic complexes were evaluated using the octanol/water shake‐flask method (see Supporting Information). Complex **3**, bearing a Ru^II^ complex conjugated to Oxa^IV^, displayed a logP of −0.81 ± 0.02, indicating high hydrophilicity. The presence of the lipophilic Au^I^ complex increased the logP value of complex **4** to 1.73 ± 0.36 and that of the tri‐metallic complex **5** to 1.05 ± 0.01. The higher logP of complex **4** relative to **3** and **5** may result in increased membrane affinity.^[^ ^22^ ^]^

### Monitoring Reduction Products

**Figure 3:**
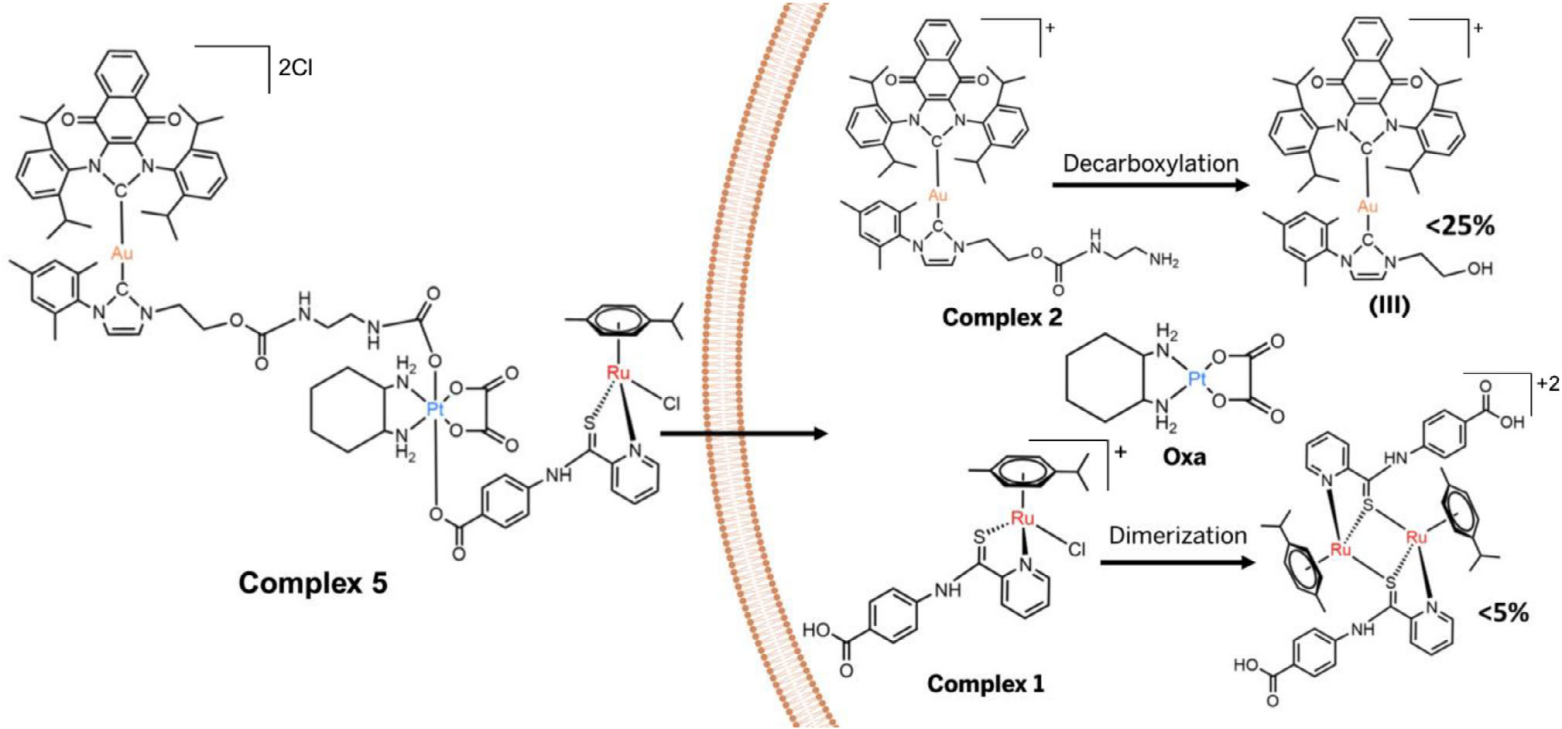
Illustration of the reduction of complex **5** that is thought to occur in the TME and upon entering a cancer cell. Evidence supporting the presumed reduction of **5** came from studies where the complex was treated with 10 equiv. of ascorbic acid at pH 7 at 37 °C. This resulted in the release of the constituent moieties and several other species. See text and Scheme 1 for details.

As noted above, Pt^IV^ complexes are subject to reduction in cancer cells and in the TME. This leads to cleavage of the axial ligands and release of the active Pt^II^ core.^[^ ^23^ ^]^ We thus monitored the reduction products of Au^I^‐Oxa^IV^‐Ru^II^ (complex **5**) under reducing conditions (10 equiv. ascorbate, 37 °C, pH 7.0) using HPLC and electron‐spray ionization mass spectrometry (Figure 3). As anticipated, the axial ligands (complex **1** (Ru^II^) and complex **2** (Au^I^)) were released alongside the Pt^II^ drug, Oxa. Additionally, we observed a hydroxyl‐Au(I) species (Scheme 1, complex **III**) (∼25%), previously reported as an ICD inducer;^[^ ^9^ ^]^ this product was presumed to arise from the partial degradation of the carbamate complex **2**. A small quantity of a Ru^II^ dimer (formed in < 5% yield) was also detected by HRMS. This latter finding is consistent with earlier reports describing Ru^II^‐F (Figure 1b) and other pyridinecarbothioamide Ru^II^ complexes forming dimers in aqueous environments (Figure S32).^[^ ^24^ ^]^

### Antiproliferative Activity

We examined the antiproliferative activity of the heterometallic complexes **3**–**5** and compared them against their monometallic constituent components, as well as a physical 1:1:1 mixture of **1 **+ Oxa + **2** after 72 h incubation across a panel of five cancer cell lines: A2780 (ovarian carcinoma), A2780cis (ovarian carcinoma resistant to cisplatin, also referred to as A2780CP), CT26 WT (murine colorectal carcinoma), A375 (melanoma), and PC9 (non‐small cell lung cancer). The results are summarized in Table 2.

**Table 2:** IC_50_ values [µM] for various cell lines after a 72 h incubation period (tested in triplicate).

**Compounds**	**Oxa**	**1**	**2**	**3**	**4**	**1+Oxa+2**	**5**
A2780	0.29 ± 0.03	15.7 ± 4.3	0.34 ± 0.08	3.8 ± 0.2	0.31 ± 0.09	0.24 ± 0.01	0.44 ± 0.04
A2780 CisR	1.01 ± 0.22	>40	0.56 ± 0.09	13.5 ± 1.1	1.60 ± 0.02	0.49 ± 0.05	1.20 ± 0.21
CT 26 WT	20.0 ± 1.4	>40	14.9 ± 0.4	23.1 ± 2.6	17.1 ± 0.9	11.7 ± 1.7	15.2 ± 1.3
A375	3.16 ± 0.49	>40	2.48 ± 0.35	26.6 ± 1.9	3.6 ± 0.4	2.4 ± 0.2	2.6 ± 0.4
PC9	8.2 ± 1.0	>40	0.49 ± 0.13	24.2 ± 0.8	0.78 ± 0.23	0.53 ± 0.09	0.80 ± 0.04

We first assessed the monometallic agents (Oxa, and complexes **1** & **2**) to establish a baseline for activity and to evaluate the individual contributions of each component. Oxa displayed low micromolar activity in the human cancer cell lines A2780, A2780cis, PC9, and A375, while showing reduced activity in CT26, with an IC_50_ of 20 µM. Complex **1** was less active in all models, a finding we ascribe to its poor cellular permeability resulting from its negatively charged carboxylate group. Complex **2**, demonstrated low micromolar activity across most cell lines, similar to Oxa. Next, we examined the heterometallic series of complexes **3**–**5** in an effort to understand whether combining complexes into a single conjugate modulated the antiproliferative effects of the constituents. General trends emerged–complex **3** (Pt^IV^–Ru^II^) showed increased activity compared to complex **1** but was not as active as Oxa, with its IC_50_ values exceeding 20 µM in most lines. Complex **4** (Pt^IV^–Au^I^) retained the potency of complex **2** but did not significantly exceed it. Complex **5** which comprises all three metal complexes, demonstrated activity comparable to the mono‐metal species, namely Oxa, complex **2**, and complex **4**, as well as a mixture consisting of a 1:1:1 molar ratio of **1 **+ Oxa + **2**. This leads us to suggest that while combining multiple bioactive moieties may not necessarily lead to enhanced potency, possibly due to differences in individual potency, the activity of its dominant constituents is preserved. These results also lead us to conclude that the cytotoxic activity of the heterometallic series **3**–**5** is primarily driven by the platinum and gold constituents, with the ruthenium moiety contributing to a lesser extent.

### Mechanism of Action Studies

Apart from serving as putative ICD inducers, the multi‐component complexes **3**–**5** were expected to interact with different cellular targets. We thus explored several targets in addition to cellular and organelle uptake after 24 h incubation. A lipoate‐to‐dihydrolipoate reduction assay in HCT‐116 cells was used to evaluate the ability of the complexes to inhibit reducing enzymes, such as lipoamide dehydrogenase, glutathione reductase, and TrxR.^[^ ^25^ ^]^ Auranofin, which is a strong inhibitor of several reducing enzymes,^[^ ^26^ ^]^ resulted in the lowest dihydrolipoate production (55% relative to control), indicating the highest degree of enzymes inhibition. The triple metal complex **5**, a 1:1:1 mixture of **1 **+ Oxa + **2**, and complex **2** followed with 60%, 65%, and 72% dihydrolipoate formation, respectively. The bimetallic complexes **3** and **4**, as well as Oxa and complex **1** showed activity similar to untreated cells (control) (Figure S33).

A number of gold and ruthenium complexes are known to inhibit members of the TrxR enzyme family.^[^ ^27^ ^]^ TrxR is upregulated in several tumors.^[^ ^28^, ^29^ ^]^ We thus sought to evaluate the activity of our gold‐ and ruthenium‐containing complexes against TrxR1 (found in the cytoplasm) and TrxR2 (found in the mitochondria). Specifically, we evaluated the ability of the monometallic complexes and the triple **1 **+ Oxa + **2** mixture (simulating the products produced from complex **5** with its rapid half‐life for reduction) to inhibit the isolated human TrxR1 & TrxR2 enzymes (Table 3). Oxa (25.6 and 28.2 µM) and auranofin (0.06 and 0.22 µM) were used as negative and positive controls, respectively. The Ru^II^ complex **1** (3.44 and 4.36 µM) outperformed Oxa and was able to inhibit both enzymes at µM levels, whereas the Au^I^ complex **2** (1.62 and 1.27 µM) showed higher inhibitory activity toward TrxR2. Equal molar ratios of **1 **+ Oxa + **2** (at 0.97 and 0.81 µM concentrations) gave rise to a synergistic effect as inferred from a combination index calculation involving both TrxR1 and TrxR2 (see Supporting Information). This latter finding was taken as a rationale for the further study of the tri‐metal complex **5**.

**Table 3:** IC_50_ values [µM] for the inhibition of human TrxR1 & TrxR2 after 75 min incubation at 37 °C (tested in triplicate).

	**Auranofin**	**Oxa**	**1**	**2**	**1+Oxa+2**
**TrxR1**	0.06 ± 0.02	25.6 ± 3.7	3.44 ± 0.32	1.62 ± 0.13	0.97 ± 0.05
**TrxR2**	0.22 ± 0.04	28.2 ± 2.9	4.36 ± 0.41	1.27 ± 0.21	0.81 ± 0.16

**Figure 4:**
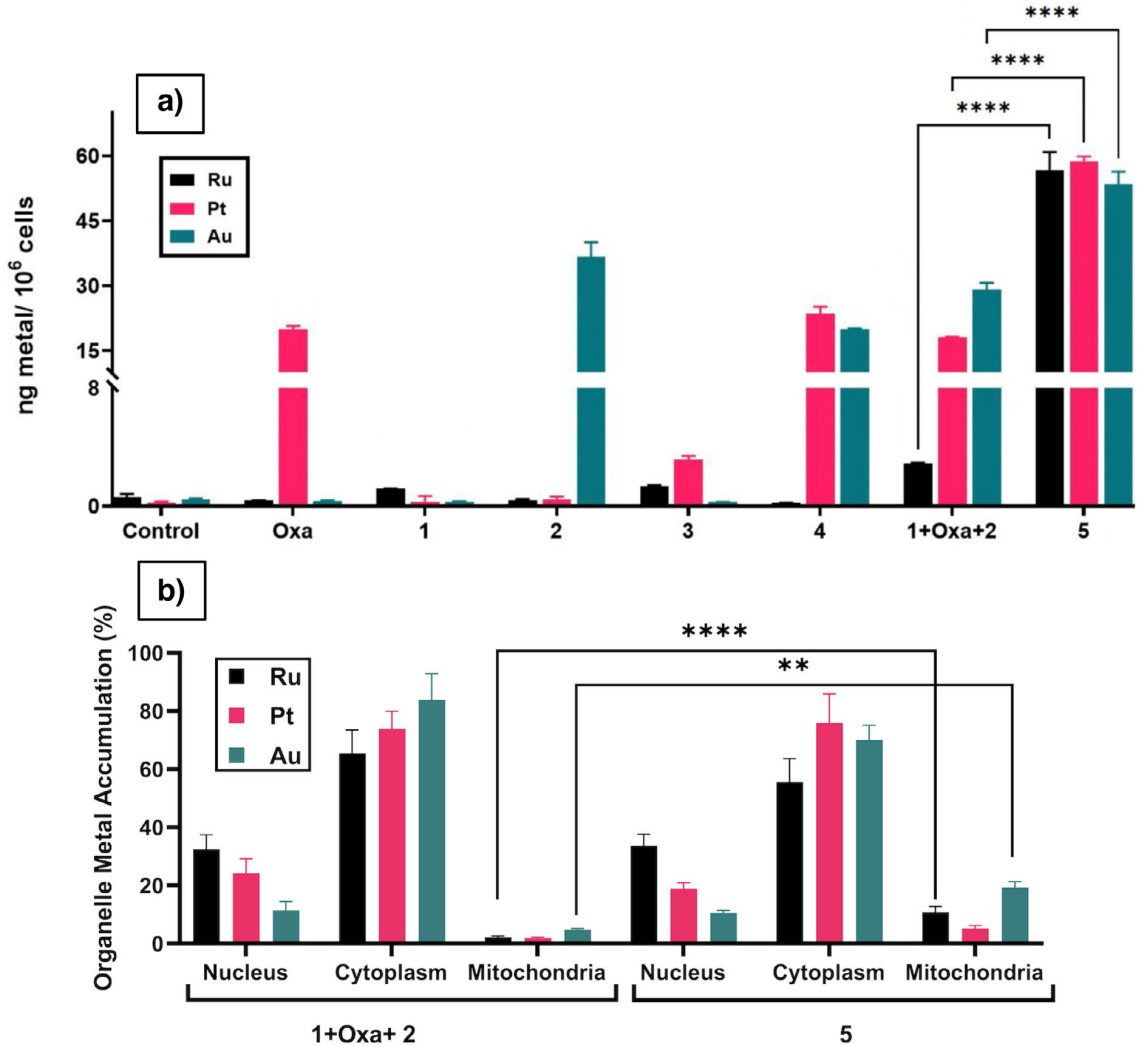
a) Total cellular metal uptake of various complexes and mixtures. b) Organelle metal accumulation of a 1:1:1 mixture of **1** + Oxa + **2** versus complex **5**. The metal levels shown in both A & B were determined by ICP‐MS after a 24 h period of incubation of HCT‐116 cells at a 1 µM concertation of the indicated complexes. **p < 0.01, ****p < 0.0001 (two‐way ANOVA).

It is important to note the TrxR assay was performed using isolated enzymes. It thus does not take into consideration cellular uptake of the complexes nor organelle distribution. Recognizing this, we incubated HCT‐116 cells with 1 µM of complexes **1**–**5** for 24 h and quantified the total metal content via ICP‐MS (Figure 4a). Complex **1** (containing only Ru^II^) showed low accumulation of ruthenium consistent with its minimal antiproliferative activity. The same proved true for complex **3** (Pt^IV^‐Ru^II^), which engendered only low levels of platinum and ruthenium accumulation; again, this finding provides a rationale for why complex **3** displays reduced cytotoxicity compared to Oxa. Complex **4** (Au^I^‐Pt^IV^) showed similar metal uptake as its monometallic counterparts, while complex **5** (Au^I^‐Pt^IV^‐Ru^II^) was found to provide for increased accumulation of all three constituent metals, but particularly ruthenium, compared to complexes **3** and **4** or the 1:1:1 **1 **+ Oxa + **2** mixture.

To understand further the metal distribution within the cell, we isolated samples from the nucleus, cytoplasm, and mitochondria from HCT‐116 cells after 24 h incubation with 1 µM of complex **5** or the 1:1:1 **1 **+ Oxa + **2** mixture. Quantification was achieved via ICP‐MS. This study was carried out to test the expectation that TrxR2 enzyme inhibition would only be seen if both gold and ruthenium were taken up into the mitochondria concurrently. The results are presented in Figure 4b. They are shown in percentages due to the starting difference in total cellular uptake. The multi‐metal complex **5** showed a statistically significant higher accumulation of gold and ruthenium in the mitochondria compared to the 1:1:1 mixture of **1 **+ Oxa + **2**. This finding leads us to suggest that an ability to inhibit TrxR2 plays a role in the mechanism of action (MOA) of complex **5**. Support for this conclusion came from the finding that complex **5** also increased ROS production in the mitochondria as inferred from confocal microscopy studies (Figure S34).

### In Vitro Assessment of DAMP Production

**Figure 5:**
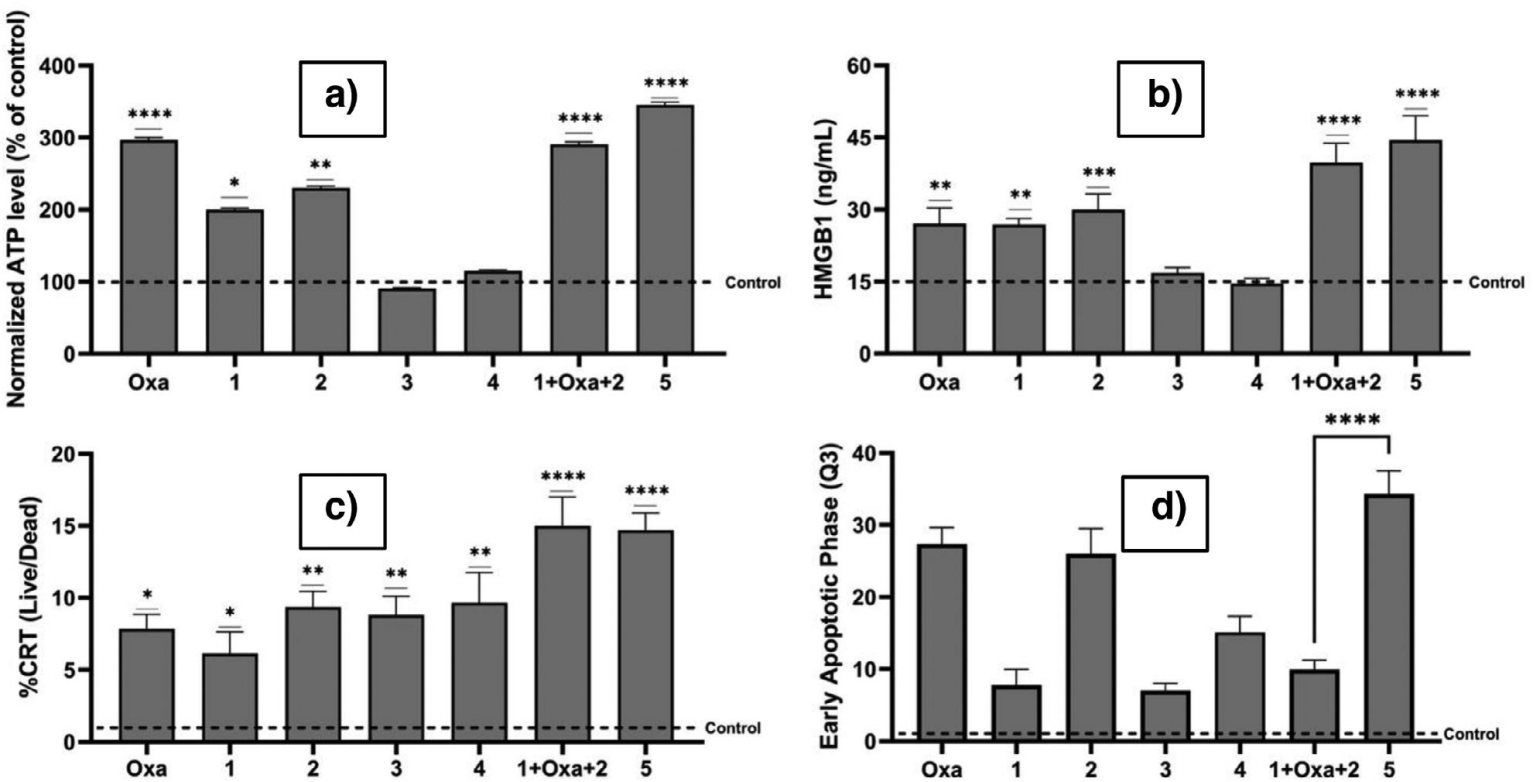
ICD DAMPs released after 24 h incubation of CT26 cells with IC50 concentrations of the indicated species. a) Extracellular ATP release. Data were normalized to the untreated control, set to 100%. b) Extracellular HMGB1 release. c) Externalized calreticulin (CRT) on the cell membrane of live versus dead CT26 cells. d) Early apoptotic phase percentage of CT26 cells as determined from the Annexin‐V quadrant Q3. *p < 0.05, **p < 0.01, ***p < 0.001, ****p < 0.0001 (one‐way ANOVA).

To investigate the immunogenic potential of the new complexes, we monitored the DAMPs associated with ICD (ATP, HMGB1, and CRT). This was done by incubating CT26 cells with IC_50_ concentrations of complexes **1**–**5** for 24 h (see Supporting Information for experimental details). ATP release was markedly elevated in cells treated with complex **5** (>300% of control). A similar increase was seen for the mixture (**1 **+ Oxa + **2**); however, significantly lower enhancements were seen for all other treatments (Figure 5a). Notably, the bimetallic complexes **3** and **4** did not induce significant ATP release relative to their monometallic counterparts or control. This lack of ATP release may reflect poor cellular uptake (complex **3**) or excessive lipophilicity that impedes intracellular distribution (complex **4**).

Extracellular HMGB1 was induced effectively by complex **5** (Figure 5b). Oxa alone produced a modest effect, consistent with its known ICD profile.^[^ ^5^ ^]^ The 1:1:1 mixture of **1 **+ Oxa + **2** yielded HMGB1 levels comparable to those of complex **5**, while the bimetallic derivatives (complexes **3** and **4**) did not induce significant release after 24 h incubation.

Surface calreticulin exposure (Figure 5c) revealed CRT translocation in all tested complexes with complex **5** (∼15%) and the physical mixture (**1 **+ Oxa + **2**), outperforming the mono and bi‐metallic derivatives. During the early stages of apoptosis, immunogenic dying cells relocate calreticulin (CRT) and ERp57 from the perinuclear endoplasmic reticulum (ER) to the cell surface. The externalization of the CRT/ERp57 complex acts as a pro‐phagocytic “eat me” signal, facilitating recognition and engulfment by dendritic cells.^[^ ^30^ ^]^ Thus, higher populations of pre‐apoptotic cells may increase the immunogenic response in vitro. An annexin‐V assay was used to determine the percentage of early apoptotic cells. As shown in Figure 5d, an increase in the percentage of early apoptotic cells was seen in the case of complex **5** (>35%) compared to the 1:1:1: mixture of **1 **+ Oxa + **2** (∼10%) (Figure S35).

Altogether, the above results provide support for the suggestion that the integrated trimetallic scaffold embodied in complex **5** can induce production of all three ICD markers (DAMPs), outperforming both the individual monometallic constituents and the congeneric bimetallic constructs.

### In Vivo Efficacy and Toxicity

**Figure 6:**
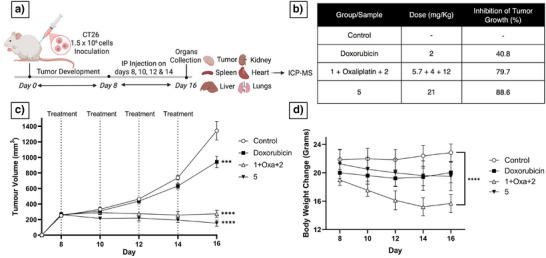
a) In vivo tumor inhibition efficacy studies in a CT26 colorectal cancer model involving i.p. injection of the indicated treatments (Created in BioRender. Babu, T., 2025). b) Dosages and percentage of solid tumor growth reduction produced by the test compounds. c) Tumor volume changes over time. d) Body weight as a function of time. ***p < 0.001, ****p < 0.0001 compared to control.

Encouraged by the in vitro results obtained with complex **5**, we compared its in vivo antitumor activity in a CT26 murine model against a 1:1:1 ratio of **1 **+ Oxa + **2**, as well as doxorubicin, an FDA approved, nonmetal drug that displays ICD effects^[^ ^31^ ^]^ and was chosen as a mechanistically distinct control. The experimental protocol consisted of inoculating mice with 1.5 x 10^6^ CT26 cells on day 0. When the tumors became palpable, a series of four intraperitoneal (i.p.) injections were carried out on days 8, 10, 12, & 14. On day 16 mice (*n* = 5) were euthanized and organs including tumors (Figure S37) were collected and analyzed for their metal content via ICP‐MS (Figure 6a). The doxorubicin dosage was based on a previous report.^[^ ^32^ ^]^ The molar equivalence dosages for complex **5** and the **1 **+ Oxa + **2** mixture were calculated based on an Oxa dose of **4** mg kg^−1^. Tumor growth reduction percentages are summarized in Figure 6b and tumor volume changes over time are shown in Figure 6c. Complex **5** displayed the highest tumor growth inhibition (∼88%) followed by **1 **+ Oxa + **2** (∼80%), both outperforming doxorubicin (∼40%). However, as shown in Figure 6d, **1 **+ Oxa + **2** was associated with acute toxicity, as inferred from the observed reduction in body weight. This was not observed in case of **5**. Thus, a clear benefit can be inferred for the combined three‐metal approach embodied in complex **5**.

**Figure 7:**
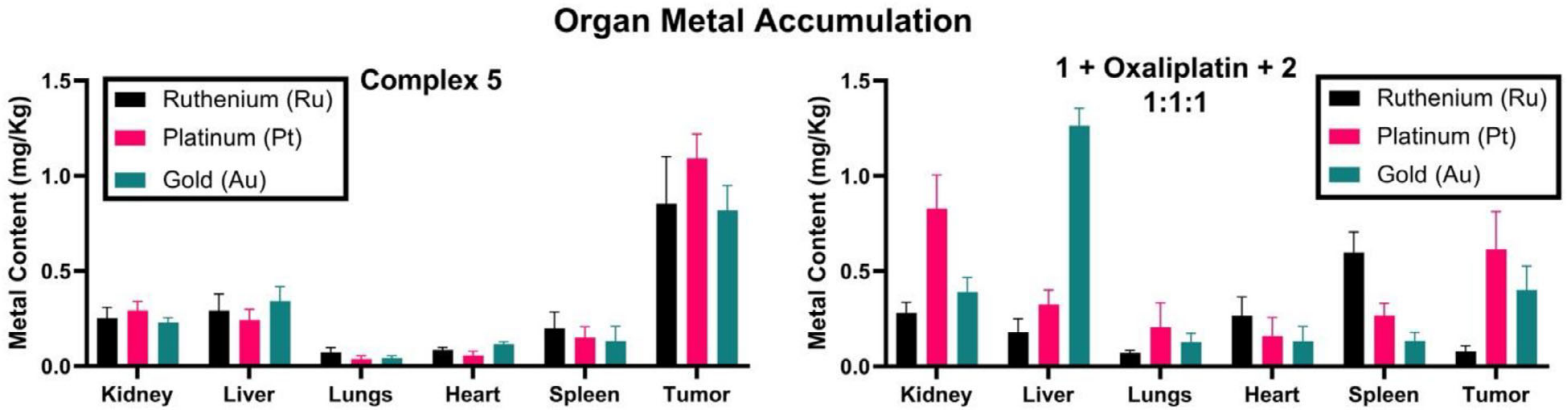
In vivo biodistribution profiles of complex **5** (left) and a 1:1:1 combination of **1** + Oxa + **2** (right) in BALB/c mice (*n* = 5) as monitored via ICP‐MS at day 16 post‐administration.

To obtain a better understanding of the differences in toxicity, we performed ICP‐MS analyses of tumor, spleen, heart, lung, kidney, and liver samples of the treated animals to quantify platinum, gold, and ruthenium content (Figure 7). Tumor and organs were mineralized using aqua regia and re‐constituted in 2% nitric acid for analysis (see Supporting Information). In the case of complex **5**, similar accumulation levels across all tested organs, as well as tumor, were seen for all three metals leading us to suggest that its stability is sufficient for in vivo use. Notably, the hepatic Au, renal Pt, and splenic Ru levels were increased for **1 **+ Oxa + **2** compared to **5**. We take this as an indication that the covalent conjugation of three metals into a single scaffold in the form of complex **5** can alter their biodistribution.

### In Vivo Challenge and White Blood Cell Monitoring

**Figure 8:**
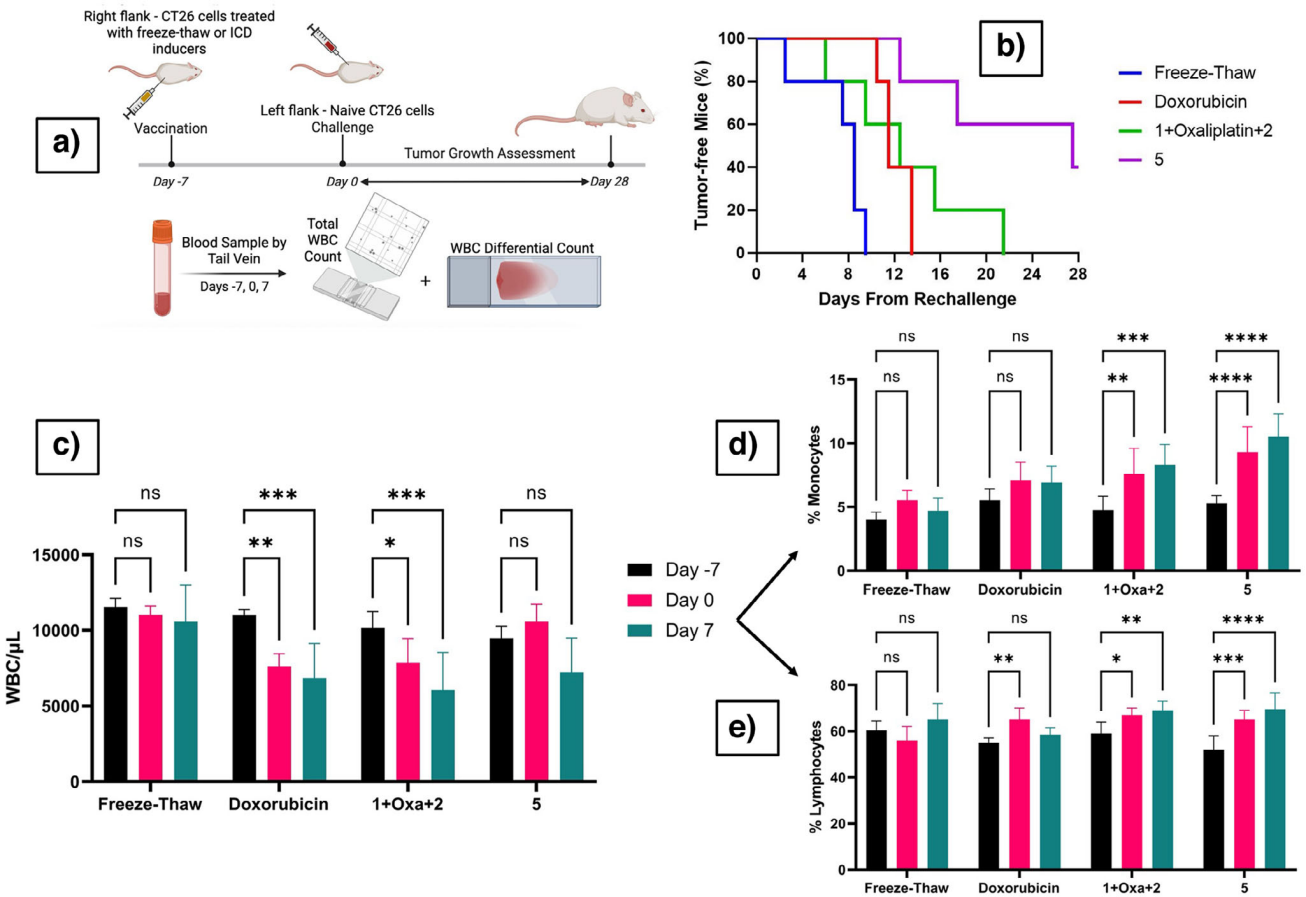
a) Protocol used for ICD challenge in vivo with blood samples collected on days ‐7, 0, and 7. b) Percent tumor free mice (*n* = 5) following challenge (created in BioRender. Babu, T., 2025). c) Total WBC count determined on different days and WBC differential percentage of d) monocytes and E) lymphocytes. *p < 0.05, **p < 0.01, ***p < 0.001, ****p < 0.0001.

Induction of an immunogenic response in vivo was determined using the established preclinical CT26 challenge model depicted in Figure 8a.^[^ ^33^ ^]^ Briefly, 1.5 x 10^6^ CT26 cells pretreated for 24 h with the species of interest at a concentration corresponding to the IC_50_ or control freeze–thaw cells were injected to the right flank (vaccination flank) of BALB/c mice (*n* = 5). After 7 days, the mice were challenged with 5 x 10^5^ naive CT26 cells injected into the left flank (challenge flank). Mice were monitored thereafter to assess the level, if any, of tumor development on the challenge (left) side. The results are summarized in Figure 8b. Monitoring was continued for 28 days. This revealed tumor free survival up to day 10 for the freeze–thaw group. These are animals in which ICD induction was not expected. Tumor free survival was seen up to day 14 and up to day 21 in the doxorubicin and **1 **+ Oxa + **2** groups, respectively. In contrast, the group treated with complex **5** displayed enhanced tumor‐free survival with 40% of the animals surviving through the end of the study period (day 28).

Chemotherapeutics that elicit ICD immunotherapy properties are expected to activate both the innate and adaptive immune systems, particularly through maturing dendritic cells that stimulate CD8 + T‐cell activation.^[^ ^34^ ^]^ Unfortunately, validating such responses in vivo typically requires complex, time‐ and resource‐intensive assays. We thus aimed to establish a potentially more facile method to monitor systemic immune engagement. Reports of changes in peripheral blood biomarkers in cancer patients receiving immunotherapies, such as PD‐L1 inhibitors, provide support for the notion that changes in peripheral leukocyte dynamics could provide a marker for drug‐induced immune activation.^[^ ^35^, ^36^ ^]^ More specifically, we hypothesized that blood‐based white blood cells (WBC) profiling—total and differential—could provide an indicator of ICD induction in vivo.

To test this proposition, whole blood samples were collected from mice before vaccination (day ‐7), before challenge (day 0), and 7 days after challenge (day 7) as outlined in Figure 8a. The samples were subjected to a manual count of total white blood cell (WBC) using Tuerk staining and differential leukocytes (monocytes, lymphocytes, neutrophils, eosinophils, and basophils) using Wright staining (see Supporting Information). The results are summarized in Figure 8c as total WBC per µL (Figure 8c), differential percentages of monocytes (Figure 8d), and lymphocytes (Figure 8e) corresponding to an innate and adaptive immune response, respectively. The neutrophil, eosinophil, and basophil levels remained unchanged. Note that mice were injected only with pretreated, dying tumor cells, not the compounds themselves; however, the cells in question could have retained the test species as the result of, e.g., intracellular accumulation. To the extent this occurred, it could influence the immune response.

As shown in Figure 8c–e, at day ‐7, no significant differences in the total WBC counts or differential profiles were observed among the treatment groups in agreement with previous mice WBC counts.^[^ ^37^ ^]^ At day 0, a significant reduction in total WBC count was observed in the doxorubicin group. This can be ascribed to the myelosuppressive effect of doxorubicin.^[^ ^38^ ^]^ A similar decrease was noted in the **1 **+ Oxa + **2** mixture group, which is thought to reflect the leukopenia side effects associated with Oxa treatment.^[^ ^39^ ^]^ In contrast, the freeze–thaw and complex **5** groups showed no statistically significant changes. These differing trends were retained through day 7. This is taken as evidence that neither the freeze–thaw control nor complex **5** induced systemic leukopenia. Differential analysis showed no change in monocyte or lymphocyte percentages in the freeze–thaw group across all time points, consistent with a lack of immunogenic DAMP release. Doxorubicin treatment led to a transient increase in lymphocyte percentages by day 0. Both the **1 **+ Oxa + **2** mixture and complex **5** groups exhibited elevated monocyte and lymphocyte percentages, with complex **5** inducing a more pronounced and sustained response. These results align well with tumor free survival and lead us to propose that WBC count monitoring could emerge as a useful tool for monitoring ICD induction.

## Conclusion

In summary, we have prepared a tri‐metallic Pt^IV^ prodrug (complex **5**) that integrates Type I and Type II ICD inducers into a single molecular scaffold. Designed to co‐release Pt^II^, Ru^II^, and Au^I^ species upon reduction, this complex offers a modular approach to multi‐pronged chemotherapy. The three different active metal species embodied in **5** were, in turn, found to inhibit TrxR1/TrxR2 and generate a robust ICD response in vitro as reflected in the production of key DAMPs. Although the in vitro IC_50_ values of complex **5** are comparable to a 1:1:1 mixture of the single agents (**1 **+ Oxa + **2**), in vivo, complex **5** was found to reduce tumor growth, provide for favorable biodistribution and reduced acute toxicity effects (e.g., weight loss) compared to a  mixture of its constituents. Complex **5** also prolonged tumor‐free survival in a mouse challenge study, and elicited sustained leukocyte activation—highlighting its dual chemo‐immunotherapeutic mode of action. This work underscores the potential of chemically integrated, multimodal ICD inducers to drive coordinated immunogenic responses. As such, it is expected to set the stage for future efforts to develop metal‐based cancer immunotherapies.

## Supporting Information

The Supporting Information accompanying this text provides experimental details and characterization data. Additional references are also cited within the Supporting Information.^[^ ^40^, ^41^, ^42^, ^43^, ^44^, ^45^ ^]^

## Conflict of Interests

The authors declare no conflict of interest.

## Supporting information



Supporting Information

## Data Availability

The data that support the findings of this study are available in the Supporting Information of this article.
