# Supplementary material for: Three Is Better than One: A Multimetal Complex that Triggers Immunogenic Cell Death
Source: Angew Chem Int Ed Engl. 2025 Aug 12;64(39):e202514351. doi: 10.1002/anie.202514351 (PMC12455390; doi:10.1002/anie.202514351)
Supplement: Supplementary file 1 — Supporting Information [file ANIE-64-e202514351-s001.docx]

Three is Better than One: A Multimetal Complex that Triggers Immunogenic Cell Death

Tomer Babu^a^, Matthew S. Levine^a^, Sourav Acharya^b^, Esther Y. Maier^c^ and Jonathan L. Sessler*^a^

[a] Department of Chemistry The University of Texas at Austin 105 East 24th Street, Austin, Texas 78712-1224, United States

[b] Institute for Drug Research, School of Pharmacy
The Hebrew University of Jerusalem
Jerusalem 9112102, Israel

[c] Innovation for Health Institute, College of Pharmacy The University of Texas at Austin 1400 Barbara Jordan Blvd, Austin, Texas 78723, United States

**Corresponding Email -** Sessler@cm.utexas.edu

# Experimental Section

## 1.1. Materials and Methods

All chemicals and solvents used in this study were procured from commercial sources and used without further purification.

The newly synthesized Au, Ru and conjugate complexes were characterized by ^1^H NMR spectral, HRMS, and elemental analyses. Complexes that contain Pt^IV^ were also characterized by ^195^Pt NMR spectroscopy. Progress of reactions was monitored by analytical HPLC (Thermo Scientific UltiMate 3000) with a reverse-phase C18 column Phenomenex Kinetex, length 100 mm, internal diameter 4.60 mm, particle size 2.6 μm, pore size 100 Å. The purity and retention time (RT) of the newly synthesized compounds reported here were measured with the same analytical HPLC system water/acetonitrile gradient at a flow rate of 1 mL/min. Reaction mixtures were purified on a preparative HPLC system (Thermo Scientific UltimaMate 3000 station) equipped with a reverse-phase C18 column (Phenomenex Luna 250 × 21.2 mm, 10 μm, 100 Å) using a similar mobile phase at a flow rate of 15 mL/min. UV spectroscopic detection was measured at 220 nm in the case of both the HPLC systems. Appropriate fractions were combined and lyophilized to yield the desired pure compounds.

All NMR spectroscopic data were collected on a Bruker AVANCE III^TM^ HD 500 MHz spectrometer. The data were processed using either MestreNova or Bruker TopSpin 3.6.0 software. ^1^H NMR chemical shifts were referenced with the individual solvent residual peaks of the respective NMR solvents. ^195^Pt NMR chemical shifts were reported with respect to the chemical shift of a standard (K_2_PtCl_4_ in water) at −1624 ppm. High resolution mass spectra (HR-MS) were recorded using a X500R QTOF by direct injection ^+^ve mode electrospray ionization. Elemental analyses were performed using a Thermo Scientific FLASH 2000 element analyzer.

## 1.2. Synthesis

**Ru^II^-F (Plecstatin-1)**

Plecstatin-1 was prepared using a reported procedure.^1^

^1^H NMR (500 MHz, DMSO-*d*_6_) δ 9.56 (d, J = 5.6 Hz, 1H), 8.18 (s, 1H), 7.73 (s, 1H), 7.47 – 7.13 (m, 5H), 6.00 (d, J = 6.0 Hz, 1H), 5.92 (d, J = 6.0 Hz, 1H), 5.84 (d, J = 6.8 Hz, 1H), 5.56 (d, J = 5.7 Hz, 1H), 2.64 (p, J = 6.9 Hz, 1H), 2.10 (s, 3H), 1.11 (d, J = 6.9 Hz, 3H), 1.03 (d, J = 6.9 Hz, 3H).

**N-(4-Benzoic acid)-2-pyridinecarbothioamide (I)**

4-Aminobenzoic acid (3.42 g, 25 mmol), sulfur (2.41 g, 75 mmol), and sodium sulfite (0.13 g, 0.5 mol%) were heated at reflux in 2-picoline (15 mL), 135ºC overnight. Afterwards, the volatiles were removed under reduced pressure and MeOH was added to form a yellow mass that was collected by filtration and washed with diethyl ether. The resulting product was recrystallized from hot methanol. Yield – 46%, 3 g.

^1^H NMR (400 MHz, DMSO-*d*_6_) δ 12.44 (s, 1H), 8.68 (ddt, J = 4.8, 1.7, 0.8 Hz, 1H), 8.50 (dq, J = 8.0, 0.9 Hz, 1H), 8.30 – 8.09 (m, 2H), 8.08 – 7.95 (m, 3H), 7.66 (ddt, J = 7.5, 4.8, 0.9 Hz, 1H). HPLC – 5.4 min (0-100% acetonitrile (ACN) in 5.84 min + 2 min constant 100% ACN).

**N-(4-Benzoic acid)-2-pyridinecarbothioamide anhydride (II)**

N-(4-Benzoic acid)-2-pyridinecarbothioamide (150 mg, 0.581 mmol) was reacted with 0.48 equiv. of 1-ethyl-3-(3-dimethylaminopropyl)carbodiimide (EDC) for 20 min in 35 mL of tetrahydrofuran (THF) : dichloromethane (DCM) 1:1 at room temperature. After confirmation of reaction completion by TLC, the reaction was concentrated under reduced pressure to 1 mL and the product was isolated by silica gel column chromatography using DCM as the mobile phase. Yield 85%, 123 mg. HPLC – 7.4 min (0-100% ACN in 5.84 min + 2 min constant 100% ACN).

**[Chlorido(η6-p-cymene)(N-(4-benzoic acid)-2-pyridinecarbothioamide)ruthenium(II)] chloride (Complex 1)**

N-(4-Benzoic acid)-2-pyridinecarbothioamide (28.7 mg, 0.11 mmol) were dissolved in MeOH and the pH was adjusted to 6.6 with HCl. [Ru(η6-p-cymene)Cl_2_]_2_ (22 mg; 0.5 equiv.) were dissolved in MeOH and added at once and stirred for 15 min at room temperature. The reaction turned dark red and completion was monitored via HPLC. After the reaction was deemed complete, the reaction mixture was concentrated to 0.5 mL under reduced pressure and the product was isolated through precipitation using excess diethyl ether and after collection by filtration, washed extensively with ether. Yield – 96%, 60 mg.

^1^H NMR (500 MHz, DMSO-*d_6_*) δ 9.49 (dd, J = 5.7, 1.5 Hz, 1H), 8.24 (dd, J = 16.0, 6.2 Hz, 1H), 8.10 (td, J = 7.7, 1.5 Hz, 1H), 8.03 – 7.95 (m, 2H), 7.66 (ddd, J = 7.3, 5.7, 1.6 Hz, 1H), 7.20 (d, J = 8.1 Hz, 2H), 5.90 (d, J = 6.0 Hz, 1H), 5.88 – 5.77 (m, 1H), 5.75 (d, J = 6.0 Hz, 1H), 5.47 (d, J = 5.9 Hz, 1H), 2.64 (hept, J = 6.9 Hz, 1H), 2.09 (s, 3H), 1.12 (d, J = 6.9 Hz, 3H), 1.02 (d, J = 6.9 Hz, 3H). HPLC – 4.34 min (0-100% acetonitrile (ACN) in 5.84 min + 2 min constant 100% ACN) ESI-HRMS (*m*/*z*) calculated for [C_23_H_24_ClN_2_O_2_RuS-Cl-H]^+^: 493.04, found 493.05; Elemental analysis; calculated for C_23_H_24_Cl_2_N_2_O_2_RuS: C 48.94, H 4.29, N 4.96% Found: C, 48.62, H 4.30, N 4.91%.

**(1,3-bis(2,6-Diisopropylphenyl)-4,9-dioxo-1,3,4,9-tetrahydro-2*H*-2λ3-naphtho[2,3-*d*]imidazol-2-yl)(1-(2-hydroxyethyl)-3-mesityl-1,3-dihydro-2*H*-2λ3-imidazol-2-yl) gold(I) (Complex III)**

This Au(I) complex was prepared using a reported procedure.^2^

^1^H NMR (500 MHz, DMSO-*d_6_*) δ 8.03 (dd, J = 5.7, 3.4 Hz, 2H), 7.91 (dd, J = 5.9, 3.3 Hz, 2H), 7.63 (t, J = 7.8 Hz, 2H), 7.51 (d, J = 1.8 Hz, 1H), 7.34 (d, J = 7.8 Hz, 4H), 7.28 (d, J = 1.8 Hz, 1H), 6.82 (s, 2H), 4.93 (t, J = 4.9 Hz, 1H), 3.58 (t, J = 5.0 Hz, 2H), 3.23 (q, J = 5.0 Hz, 2H), 2.58 – 2.51 (m, 8H), 2.40 (s, 3H), 1.56 (s, 6H), 1.05 (dd, J = 6.8, 2.8 Hz, 20H). HPLC – 6.7 min (0-100% ACN in 5.84 min + 2 min constant 100% ACN)

**(1,3-bis(2,6-Diisopropylphenyl)-4,9-dioxo-1,3,4,9-tetrahydro-2*H*-2λ3-naphtho[2,3-*d*]imidazol-2-yl)(1-(2-((((2,5-dioxopyrrolidin-1-yl)oxy)carbonyl)oxy)ethyl)-3-mesityl-1,3-dihydro-2*H*-2λ3-imidazol-2-yl) gold(I) (Complex IV)**

30 mg (0.03 mmol) of (1,3-bis(2,6-diisopropylphenyl)-4,9-dioxo-1,3,4,9-tetrahydro-2*H*-2λ3-naphtho[2,3-*d*]imidazol-2-yl)(1-(2-hydroxyethyl)-3-mesityl-1,3-dihydro-2*H*-2λ3-imidazol-2-yl) gold(I) were reacted with 1 equiv. DMAP (3.3 mg) and 10 equiv. N,N′-disuccinimidyl carbonate (DSC) (70 mg) for 60 min in ACN. Afterwards, the product, referred to as the activated form, was purified using prep-HPLC (50-100% ACN in 0.1% TFA in 7.5 min + 5 min constant 100% ACN) and lyophilized to give a pale-yellow solid as the TFA salt. Yield – 79%, 26.8 mg. HPLC – 6.7 min (0-100% ACN in 5.84 min + 2 min constant 100% ACN)

**(1-(2-(((2-Aminoethyl)carbamoyl)oxy)ethyl)-3-mesityl-1,3-dihydro-2*H*-2λ3-imidazol-2-yl)(1,3-bis(2,6-diisopropylphenyl)-4,9-dioxo-1,3,4,9-tetrahydro-2*H*-2λ3-naphtho[2,3-*d*]imidazol-2-yl)gold (I) TFA salt (Complex 2)**

20 mg (0.02 mmol) of (1,3-bis(2,6-diisopropylphenyl)-4,9-dioxo-1,3,4,9-tetrahydro-2H-2λ3-naphtho[2,3-d]imidazol-2-yl)(1-(2-((((2,5-dioxopyrrolidin-1-yl)oxy)carbonyl)oxy)ethyl)-3-mesityl-1,3-dihydro-2H-2λ3-imidazol-2-yl) gold(I) were dissolved in 5 mL ACN and 8 equiv. of ethylenediamine were dissolved in 2 mL ACN and added dropwise while vigorously stirring for 10 min at room temperature. Afterwards, the reaction volume was reduced to 0.5 mL under reduced pressure and the product precipitated using excess diethyl ether. After collection by filtration, it was washed 3 times with ether. Yield- 90%, 17 mg.

For biological work, this compound was isolated as its TFA salt using prep-HPLC (0-100% ACN in 0.1% TFA in 15 min + 5 min constant 100% ACN).

^1^H NMR (500 MHz, DMSO-*d_6_*) δ 8.03 (dd, J = 5.7, 3.3 Hz, 2H), 7.92 (dd, J = 5.8, 3.3 Hz, 2H), 7.63 (s, 2H), 7.52 (s, 2H), 7.34 (d, J = 7.8 Hz, 4H), 6.83 (s, 1H), 6.53 (s, 2H), 3.79 – 3.75 (m, 4H), 3.16 (q, J = 6.5 Hz, 2H), 2.81 (t, 2H), 2.55 (q, J = 6.8 Hz, 8H), 2.41 (s, 3H), 1.92 (s, 2H), 1.56 (s, 6H), 1.05 (dd, J = 6.8, 3.2 Hz, 20H). HPLC – 5.64 min (0-100% ACN in 5.84 min + 2 min constant 100% ACN) ESI-HRMS (*m*/*z*) calculated for [C_52_H_62_AuN_6_O_4_]^+^: 1032.07, found 1031.44; Elemental analysis calculated for C_54_H_62_AuF_3_N_6_O_5_: C 57.44, H 5.54, N 7.44% Found: C, 57.26, H 5.34, N 7.11 %.

**Oxali(MSC)(OH) (Complex V)**

This mono-activated oxaliplatin(IV) was prepared using a reported procedure.^3^

**Oxali(N-(4-Benzoic acid)-2-pyridinecarbothioamide)(OH) and Oxali(N-(4-Benzoic acid)-2-pyridinecarbothioamide)(MSC) (Complex VI)**

40 mg (0.09 mmol) of Pt(IV) oxali(OH)_2_ were suspended in 4 mL of DMSO to which 51 mg (1.1 equiv.) N-(4-benzoic acid)-2-pyridinecarbothioamide anhydride were added. The reaction was allowed to proceed overnight at 40ºC. The following day, the formation of the product was confirmed using ^195^Pt NMR spectroscopy and analytical HPLC. It was extracted using diethyl ether to give a bright yellow solid. Yield – 78%, 48.5 mg.

For the preparation of oxali(IV)-Ru(II) (H), oxali(N-(4-benzoic acid)-2-pyridinecarbothioamide)(OH) was dissolved in MeOH and further purified via prep-HPLC (0-100% ACN in water over 15 min). HPLC – 4.5 min (0-100% acetonitrile (ACN) in 5.84 min + 2 min constant 100% ACN)

For activation of the second axial position, 5 equiv. of N,N′-disuccinimidyl carbonate (DSC) were added *in situ* after an overnight reaction over the course of 1 hr at room temperature. The product was then purified using prep-HPLC (0-100% ACN in water over 15 min) and lyophilized. Yield - 97%, 50 mg (based on 25.7 mg of oxali(OH)_2_ starting material). HPLC – 4.9 min (0-100% acetonitrile (ACN) in 5.84 min + 2 min constant 100% ACN)

**[Oxali(IV)-Ru(II)] chloride (Complex 3)**

Lyophilized oxali(N-(4-benzoic acid)-2-pyridinecarbothioamide)(OH) (G) (29.9 mg, 0.04 mmol) were dissolved in 1.5 mL of MeOH and in a different vial, 14.3 mg [Ru(η6-p-cymene)Cl_2_]_2_ (0.5 equiv.) were dissolved in 1 mL MeOH. Both solutions were combined in a dropwise manner with stirring over the course of 10 min at room temperature. Once the reaction was deemed complete, the solution was concentrated under reduced pressure to a volume of 0.5 mL. The product was precipitated with excess diethyl ether and after being collected by filtration the product was washed several times with ether. Yield – 87%, 37.8 mg.

^1^H NMR (500 MHz, DMSO-*d_6_*) δ 9.47 (d, *J* = 6.1 Hz, 1H), 8.33 (s, 1H), 8.08 (t, *J* = 7.8 Hz, 1H), 7.93 – 7.86 (m, 2H), 7.65 (t, *J* = 6.3 Hz, 1H), 7.41 (s, 1H), 7.14 (s, 2H), 5.88 – 5.72 (m, 3H), 5.45 (d, *J* = 6.0 Hz, 1H), 2.77 – 2.55 (m, 4H), 2.36 – 2.25 (m, 1H), 2.10 – 2.08 (m, 3H), 1.58 – 1.50 (m, 4H), 1.21 – 1.03 (m, 8H). ^195^Pt NMR (108 MHz, DMSO) δ 1406.46. HPLC – 3.91 min (0-100% ACN in 5.84 min + 2 min constant 100% ACN) ESI-MS (*m*/*z*) calculated for [C_31_H_38_ClN_4_O_7_PtRuS-Cl-H]^+^: 905.87, found 906.10; Elemental analysis calculated for C_31_H_38_Cl_2_N_4_O_7_PtRuS: C 38.08, H 3.92, N 5.73% Found: C, 37.92, H 3.81, N 5.81%.

**[Oxali(IV)-Au(I)] PF_6_ (Complex 4)**

30 mg (0.026 mmol) of (1-(2-(((2-aminoethyl)carbamoyl)oxy)ethyl)-3-mesityl-1,3-dihydro-2H-2λ3-imidazol-2-yl)(1,3-bis(2,6-diisopropylphenyl)-4,9-dioxo-1,3,4,9-tetrahydro-2H-2λ3-naphtho[2,3-d]imidazol-2-yl)gold(I) were dissolved in 3 mL of ACN and 2 equiv. of triethylamine (TEA) were added at once. Separately, 18.2 mg of oxali(MSC)(OH) were dissolved in 1 mL DMF and added dropwise to the gold solution (complex **2**) under stirring for 3 h in room temperature. After confirmation of product formation and reaction completion by analytical HPLC, the product was purified using prep-HPLC (50-100% ACN in 0.1% PF_6_ over 12.5 min + 5 min constant 100% ACN) and lyophilized as PF_6_ salt. Yield – 15.2 mg, 38.5%.

^1^H NMR (500 MHz, DMSO-*d_6_*) δ 8.03 (dd, J = 5.7, 3.3 Hz, 2H), 7.91 (dd, J = 5.8, 3.3 Hz, 2H), 7.63 (t, J = 7.8 Hz, 2H), 7.51 (s, 1H), 7.35 (d, J = 7.8 Hz, 4H), 7.06 (s, 2H), 6.82 (s, 2H), 3.83 – 3.71 (m, 7H), 2.97 – 2.92 (m, 5H), 2.91 – 2.83 (m, 2H), 2.54 (q, J = 6.0 Hz, 6H), 2.41 (s, 3H), 2.16 – 1.99 (m, 2H), 1.56 (s, 6H), 1.49 (t, J = 15.4 Hz, 4H), 1.32 (s, 1H), 1.05 (dd, J = 6.8, 4.3 Hz, 25H). ^195^Pt NMR (108 MHz, DMSO-*d_6_*) δ 1417.35. HPLC – 6.03 min (0-100% ACN in 5.84 min + 2 min constant 100% ACN) ESI-HRMS (*m*/*z*) calculated for [C_61_H_76_AuN_8_O_11_Pt]^+^: 1489.37, found 1489.49; Elemental analysis calculated for C_61_H_76_AuF_6_N_8_O_11_PPt: C 44.83, H 4.69, N 6.86% Found: C, 44.74, H 4.39, N 6.91%.

**Oxali(N-(4-benzoic acid)-2-pyridinecarbothioamide)((1-(2-(((2-aminoethyl)carbamoyl)oxy)ethyl)-3-mesityl-1,3-dihydro-2*H*-2λ3-imidazol-2-yl)(1,3-bis(2,6-diisopropylphenyl)-4,9-dioxo-1,3,4,9-tetrahydro-2*H*-2λ3-naphtho[2,3-*d*]imidazol-2-yl)gold (I)) (Complex VII)**

Oxali(N-(4-benzoic acid)-2-pyridinecarbothioamide)(MSC) (21 mg, 0.026 mmol, 1.3 equiv.) were dissolved in 2 mL DMF and in a different vial, 21 mg of (1-(2-(((2-aminoethyl)carbamoyl)oxy)ethyl)-3-mesityl-1,3-dihydro-2H-2λ3-imidazol-2-yl)(1,3-bis(2,6-diisopropylphenyl)-4,9-dioxo-1,3,4,9-tetrahydro-2H-2λ3-naphtho[2,3-d]imidazol-2-yl)gold (I) (1 equiv.) and 2 equiv. of TEA were dissolved in 2 mL of DMF and added dropwise to the oxaliplatin solution over the course of 1 h at room temperature. Once the reaction was deemed complete via analytical HPLC, the product was purified using prep-HPLC (0-100% ACN in 0.1% TFA in 15 min + 5 min constant 100% ACN) and lyophilized as the TFA salt. Yield – 73.5%, 25 mg. HPLC – 6.7 min (0-100% ACN 5.84 min + 2 min constant 100% ACN).

**[Au(I)-oxali(IV)-Ru(II)] chloride (Complex 5)**

The triple metal complex **5** was prepared by dissolving in two separate vials, 20 mg (0.011 mmol) of lyophilized oxali(N-(4-benzoic acid)-2-pyridinecarbothioamide)((1-(2-(((2-aminoethyl)carbamoyl)oxy)ethyl)-3-mesityl-1,3-dihydro-2H-2λ3-imidazol-2-yl)(1,3-bis(2,6-diisopropylphenyl)-4,9-dioxo-1,3,4,9-tetrahydro-2H-2λ3-naphtho[2,3-d]imidazol-2-yl)gold (I)) in 1 mL MeOH and 10 mg [Ru(η6-p-cymene)Cl_2_]_2_ (1.4 equiv.) in 1 mL MeOH. The reagents were then combined at once and the mixture allowed to stir for 10 min in room temperature. After the reaction was deemed complete via analytical HPLC, the reaction volume was concentrated to 0.5 mL. The product was then precipitated with excess diethyl ether and after collecting by filtration washed several times with ether. Yield – 85%, 19.3 mg.

^1^H NMR (500 MHz, DMSO-*d_6_*) δ 9.43 (d, J = 5.8 Hz, 1H), 7.97 (ddd, J = 58.9, 5.9, 3.4 Hz, 5H), 7.62 (dt, J = 19.3, 7.5 Hz, 4H), 7.35 (d, J = 7.7 Hz, 4H), 7.10 – 6.99 (m, 3H), 6.83 (s, 2H), 5.84 – 5.76 (m, 3H), 5.67 (d, J = 5.9 Hz, 1H), 5.39 (d, J = 6.1 Hz, 1H), 4.86 (d, J = 5.2 Hz, 1H), 4.62 (t, J = 5.7 Hz, 1H), 4.05 – 3.88 (m, 4H), 3.75 (s, 6H), 3.39 (q, J = 7.0 Hz, 4H), 3.05 – 2.56 (m, 14H), 2.41 (s, 3H), 2.31 – 2.25 (m, 2H), 2.08 (d, J = 10.6 Hz, 2H), 1.57 (s, 6H), 1.27 – 1.00 (m, 30H). ^195^Pt NMR (108 MHz, DMSO-*d_6_*) δ 1601.30. HPLC – 5.99 min (0-100% ACN in 5.84 min + 2 min constant 100% ACN) ESI-HRMS (*m*/*z*) calculated for [C_84_H_98_AuClN_10_O_12_PtRuS-Cl-H]^2+^: 981.97, found 981.76; Elemental analysis calculated for C_84_H_98_AuCl_3_N_10_O_12_PtRuS: C 48.71, H 4.77, N 6.76% Found: C, 48.97, H 4.70, N 6.91%.

## 1.3. Reduction and Stability

The dual and triple metal complexes **3**-**5** were dissolved, respectively, in MeOH. A concentrated solution of ascorbic acid was prepared in 1:1 MeOH : H_2_O, and the pH was adjusted to 7.0 with NaOH. A 10-fold molar excess of the ascorbic acid solution was added to the complexes and the expected reduction monitored by analytical HPLC. The half-lives (t_1/2_) for the reduction of all three complexes were determined via the linear fitting of ln(A_t_/A_0_) vs time (t) in accord with the pseudo first order rate equation A_t_ = A_0_e^−kt^, where A_0_ and A_t_ are the integrated areas of the HPLC peaks of the respective complexes at t = 0 and at time t, respectively, and k (slope) is the rate constant. The value of t_1/2_ was calculated using the equation t_1/2_ = -ln(2)/k.

Stability studies were conducted by dissolving the complexes in 1% DMSO and RPMI medium at pH 7.4, and maintaining them at 37°C. HPLC chromatograms were recorded until the peak areas were less than half the starting peak area. The t_1/2_ value was calculated by linear fitting of ln(A_t_/A_0_) vs time (t) where A_t_ and A_0_ are the integrated peak areas determined by HPLC at t = 0 and at time t.

## 1.4. Shake-Flask LogP Measurements

The octanol-water partition coefficients for the metal complexes **3**-**5** were determined using a modification of a reported shake-flask method.^4^ Water (50 mL) and n-octanol (50 mL) were shaken together for 72 hours to allow saturation of both phases. A solution of each complex was prepared in the aqueous phase with the addition of 100 µL of DMSO. An equal volume of octanol was then added. Due to the stability of the complexes in octanol and water, the biphasic solutions were mixed for ten minutes and then allowed to sit for 15 minutes. Samples were then centrifuged for 15 minutes at 3000 g to allow separation. Aliquots of each phase were taken and further spun in a microcentrifuge carefully withdrawing samples for analysis. The concentrations of the complexes in both phases were determined by UV-Vis spectroscopy. The reported logP values are defined as log{[complex]octanol/[complex]water}. Final values are reported as the mean of three independent determinations.

## 1.5. Cell Culture Maintenance

A375 human malignant melanoma cells, PC9 human non-small cell lung carcinoma cells, and CT26 WT murine colon carcinoma cells were maintained in DMEM (Dulbecco's Modified Eagle Medium) (high glucose, no glutamine) supplemented with L-glutamine solution (2% v/v; final concentration 4 mM), 10% (v/v) heat-inactivated fetal bovine serum, and 1% (v/v) Pen-Strep solution (containing penicillin: 10,000 units/mL and streptomycin: 10 mg/mL) and were passaged twice a week. A2780 human ovarian carcinoma cells, and A2780cis (cisplatin-resistant) ovarian carcinoma cells were cultured in RPMI 1640 (no glutamine) supplemented with heat-inactivated fetal bovine serum (10% v/v), gentamycin sulfate solution (50 µg/mL) and L-glutamine solution (1% v/v; final concentration 2 mM). Once a week, a solution of cisplatin in RPMI medium (final concentration: 1 μM) was added to the A2780cis cells to maintain their resistance. RPMI1640, DMEM high glucose, L-glutamine solution, and then Pen-Strep solution were purchased from Sartorius; heat-inactivated fetal bovine serum (non-USA origin, sterile-filtered) was purchased from Sigma. A375, PC9, A2780, and A2780cis cells were obtained from the American Type Culture Collection (ATCC, Rockville, MD), the CT26 WT cells were a gift given from Prof. Galia Blum’s lab.

## 1.6. Antiproliferative assay in tumorigenic cells

A375 melanoma cells (5000 cells/well), PC9 small lung cell carcinoma cells (5000 cells/well), CT26 WT murine colon carcinoma cells (5000 cells/well), A2780 ovarian carcinoma cells (5000 cells/well), and A2780cis cisplatin-resistant ovarian carcinoma cells (7000 cells/well), were seeded in flat-bottom 96-well plates in their respective cell culture media (100 µL) and incubated at 37°C in a 5% CO_2_ environment for 24 h. After that, the media were replaced with the fresh media. Stock solutions of the compounds in DMSO or in cell culture medium (for Oxa) were freshly prepared and diluted with the respective cell culture medium to graded concentrations (final concentration of DMSO_max_: 0.5% v/v). After 72 h of exposure, the media were removed and 100 µL of 0.5 mg/mL 3-(4,5-dimethylthiazol-2-yl)-2,5-diphenyl tetrazolium bromide (MTT) in DMEM or RPMI were added and the plate in question further incubated for an additional 1 h. The media containing the MTT solution was removed and the resulting formazan crystals were dissolved in 100 µL DMSO. The absorbance of the solution in each well was read at 570 nm using a BioTek Cytation3 plate reader. The IC_50_ value, which is the drug concentration needed for 50% reduction in survival, based on the survival curve, was calculated by fitting nonlinear curves in GraphPad Prism 5 Ver 5.03, using a normalized response variable slope model constructed by plotting cell viability (%) vs log of the concentration of the complex in question in nM. The results are presented as the mean values of three independent experiments.

## 1.7. Lipoate Reduction Assay

Lipoate reduction was evaluated using our previously reported protocol.^5^

In short, HCT-116 cells were harvested and seeded at a density of 10,000 cells/well in 96-well culture plates. Following an overnight incubation at 37°C under a 5% CO_2_ atmosphere, the medium was replaced, and the cells incubated with 1 μM of each complex for additional 24 hr. Thereafter, the media were removed from each well, which were then washed with 200 μL of HBSS solution. Finally, 100 µL of 5 mM lipoate and 1 mM of DTNB solutions were added to each well. The absorbance of each well at 405 nm was recorded immediately and once every 20 min for one hour using a microplate reader. Plates were covered with aluminum foil between readings.

## 1.8. Human TrxR1 vs TrxR2 inhibition

Human TrxR1 and TrxR2 were purchased from SeLENOZYME and inhibition studies were carried out by means of a DTNB assay in 96-well culture plates. Enzymes were diluted to achieve a concentration of 3.58 U mL^−1^. The compounds subject to testing were freshly dissolved in DMF. 10 μL aliquots of the enzyme solution and 10 μL of solutions of the compounds at graded concentrations or 10 μL of 1% DMF in PBS (positive control) were added and incubated for 75 min at 37 °C. A second control consisted of 10 μL of the highest concentration of each compound with 10 μL of PBS. After the chosen incubation time, 10 μL of NADPH (10 mM), 160 μL TE buffer (50 mM Tris-HCl, 2 mM EDTA, pH 7.5) and 10 μL ethanolic DTNB (50 mM) were added producing a total reaction volume of 200 μL. After proper mixing, the formation of 5-TNB was monitored with a microplate reader at 405 nm. This was done 10 times at 30 second intervals over the course of about 5 min. A TNB standard curve (0-2 mM in 200 µL TE buffer) was used to calculate the exact DTNB consumed in the reaction with linear region slopes being calculated by plotting A_405_ vs time (min). The IC_50_ values representing the concentration of compound serving to decrease the enzymatic activity of the untreated control by 50% were calculated and are given as the mean and standard error of three repeated experiments. The combination index (CI) was calculated using the Chou-Talalay method^6^

$$Cl=\frac{\left( D \right)1}{\left( Dx \right)1}+ \frac{\left( D \right)2}{\left( Dx \right)2}+\frac{\left( D \right)3}{\left( Dx \right)3}$$

where (D)_1_, (D)_2_  and (D)_3_ are the respective combination doses of compound 1 and compound 2 that yield an effect of 50% growth inhibition, with (Dx)_1_, (Dx)_2_ and (Dx)_3_ being the corresponding single doses for compounds 1, 2 & 3 that result in the same effect. A CI value of less than, equiv. to, and greater than 1 indicates synergy, additivity, and antagonism, respectively. The 1:1:1 mixture of **1**+Oxa+**2** yielded a CI score of 0.92 & 0.85 against TrxR1 and TrxR2 respectively.

## 1.9. Total Cellular Metal Accumulation

1 x 10^6^ HCT-116 cells were seeded in a 6-well plate (2 mL RPMI-1640). After incubating overnight, the medium was replaced, and the cells were treated with the test compounds (1 μM) for 24 h. The resulting cell monolayers were washed twice with cold PBS, harvested and counted in PBS for a total volume of 400 µL. Samples were divided, and each was mineralized with 1 mL aqua regia and evaporated to dryness on a 110 ºC hot plate overnight. Later, 1 mL of 2% HCl + 1% thiourea was added to each vial. Samples were quantified via ICP-MS. Metal accumulation levels were determined using the relative isotopic prevalence of ^195^Pt, ^101^Ru, and ^197^Au.

## 2.0. Metal Accumulation in Organelles

10 x 10^6^ HCT-116 cells were seeded in T-150 flasks (50 mL RPMI-1640). After incubating overnight, the medium was replaced and the cells were treated with either a 1:1:1 mixture of **1**+Oxa+**2** or complex **5** at 1 μM concentration for 24 h. The resulting cell monolayers were washed twice with cold PBS, harvested with trypsin, counted and resuspended in PBS for a total volume of 1000 µL. Isolation of nuclei, cytoplasm, and mitochondria was carried out using a reported procedure.^7^ Samples were quantified via ICP-MS. Metal accumulations were determined using the relative isotopic prevalence of ^195^Pt, ^101^Ru, and ^197^Au.

## 2.1. Annexin-V Apoptosis Assay

1 x 10^6^ CT-26 cells were seeded in a 6-well plate (2 mL RPMI-1640). After incubating overnight, the medium was replaced, and the cells were treated with the test complexes at their respective IC_50_ concentrations for 24 h. The cells were harvested using 0.5 mL Trypsin-EDTA (0.25%) and plated cells were obtained by centrifugation. Afterwards, the cells were stained using the Invitrogen Dead Cell Apoptosis Kit (Catalog V13241) with Annexin V Alexa Fluor 488 & propidium iodide and analyzed using a Cytek Northern Lights flow cytometer.

## 2.2. CRT Translocation Quantification

1 x 10^6^ CT-26 cells were seeded in a 6-well plate (2 mL RPMI-1640). After incubating overnight, the medium was replaced, and the cells were treated with the test complexes at their respective IC_50_ concentrations for 24 h. The cells were harvested using 0.5 mL Trypsin-EDTA (0.25%) and plated cells were obtained by centrifugation. CRT translocation was determined by reported procedure^8^ and analyzed using a Cytek Northern Lights flow cytometer.

## 2.3. Quantification of ATP Release

1 x 10^6^ CT-26 cells were seeded in a 6-well plate (2 mL RPMI-1640). After incubating overnight, the medium was replaced, and the cells were treated with the test complexes at their respective IC_50_ concentration for 24 h. The ATP release of the medium was quantified using Invitrogen ATP Determination Kit (Catalog A22066) according to the manufacturer’s instructions.

## 2.4. HMGB1 ELISA Assay

1 x 10^6^ CT-26 cells were seeded in a 6-well plate (2 mL RPMI-1640). After overnight incubation, the medium was replaced and the cells were treated with the test complexes at their respective IC_50_ concentration for 24 h. The HMGB1 release of the supernatant was quantified using HMGB1 Detection Kit ELISA (Chondrex, Catalog 6010) according to the manufacturer’s instructions.

## 2.5. Living Cells ROS Detection Confocal Microscopy

HCT-116 Tumor cells were seeded at a density of 1 x 10^5^ cells overnight in 35 mm glass dishes of 10 mm diameter. Afterwards, cells in the control group (no treatment) were subject to a medium change while others were incubated with a 1:1:1 mixture of **1** + oxaliplatin + **2** or complex **5** for 24 h at the respective IC_50_ concentrations. Post-incubation, the media were removed and the cells were washed (2x) with PBS. Cells were then incubated with 1 μM CM-H2DCFDA in the medium at 37 °C for 15 min. The dye-medium solution was removed, and the cells were washed with PBS (2x) before 50 nM Mitotracker^®^ Red in the medium were added and allowed to incubate for 30 min at 37 ºC. Later, the dye was washed off with PBS and 5 ug/mL Hoechst 33342 were added and the cells were allowed to stand for 30 min. Lastly, the cells were washed twice with PBS and RPMI1640 (free of phenol red) was added. The cells were then imaged fluorescently on a Nikon AXR-NSPARC laser confocal microscope while incubating at 37 ºC.

## 2.6. *In Vivo* Tumor Inhibition Efficacy

Procedures were conducted in accordance with the Guide for the Care and Use of Laboratory Animals (U.S. Public Health Service, National Institute of Health) and the protocol for this experiment was approved by the Institutional Animal Care and Use Committee (IACUC, protocol numbers AUP-2022-00101 & AUP-2024-00337) at The University of Texas at Austin. Briefly, 6-8 weeks female BALB/c mice were acclimatized for 7 days thereafter, murine colon carcinoma cells, CT26 (1.5 x 10^6^ cells in 100 μL of sterile PBS) were subcutaneously injected to the right flank. The experiments were initiated once tumors became palpable on Day 8. At this point, the animals were separated to 4 groups (n = 5). The animals were monitored for the development of pain or distress symptoms with the body weight and tumor size also being closely monitored. Control mice, treated with 100 µL of sterile saline, as well as those treated with doxorubicin 2 mg/kg, a physical mixture consisting of complex **1** (5.7 mg/kg) + Oxa (4 mg/kg) + complex **2** (12 mg/kg), and complex **5** (21 mg/kg) were administrated i.p. on days 8, 10, 12 & 14. On day 16, mice were euthanized, and tumor and organs (liver, kidney, spleen, heart and lungs) were collected for ICP-MS metal quantification.

## 2.7. Metal Accumulation *In Vivo*

Organs were weighed and mineralized with aqua regia for 3 h. Afterwards, solutions were completely dried at 110 ºC and 1000 µL of 2% HNO_3_ was added. The gold, platinum and ruthenium content of each sample was determined by ICP-MS.

## 2.8. *In Vivo* Tumor Rechallenge

All animals were group-housed in a semi-barrier animal facility and kept in clear cages with water and laboratory food pellets available. Procedures were conducted in accordance with the Guide for the Care and Use of Laboratory Animals (U.S. Public Health Service, National Institute of Health) and the protocol for this experiment was approved by the Institutional Animal Care and Use Committee (IACUC, protocol number AUP-2024-00337) at The University of Texas at Austin. Immunocompetent female BALB/c mice (Jackson Laboratory, Stock No: 000651; 6-8 weeks at the beginning of the experiment) were used for these studies.

CT26 cells were pretreated with IC_50_ concentrations of the test compounds for 24 h prior to inoculation or only subjected to medium change (control group). Animals were separated to 4 groups (n = 5) and were injected with 1.5*10^6^ cells to the right flank treated with 3X freeze-thaw cells, doxorubicin, a mixture of 1 + 2 + oxaliplatin or complex **5**. After 7 days, mice were inoculated s.c. with 5 x 10^5^ naive CT26 cancer cells into the contralateral flank. Prior to all injections, the supernatant of one flask per harvest day was tested for mycoplasma contamination (MycoAlert™, Lonza, Rockland, ME), which yielded negative results on all days. Body weights and tumor measurements were checked daily, and tumor development was compared among groups.

## 2.9. Total WBC and Differential Manual Count

Blood samples were collected from all mice groups by tail vein and were analyzed for total WBC count and differential count.^9^ Blood was draw on Day -7 (before vaccination), Day 0 (before rechallenge) and Day 7. Total WBC was analyzed by adding 2 µL of blood to 38 µL Tuerk solution (Sigma-Aldrich product - 93770) and was counted using a hemocytometer after 45 min under a 10x light microscope. Differential counting was done by preparing blood smears of blood samples, letting them air dry, fixing with methanol, and staining with Wright Stain solution (Sigma-Aldrich product - 45253). 200 WBC cells were counted and classified under 1000x magnification using a Leica DMI6000b microscope.


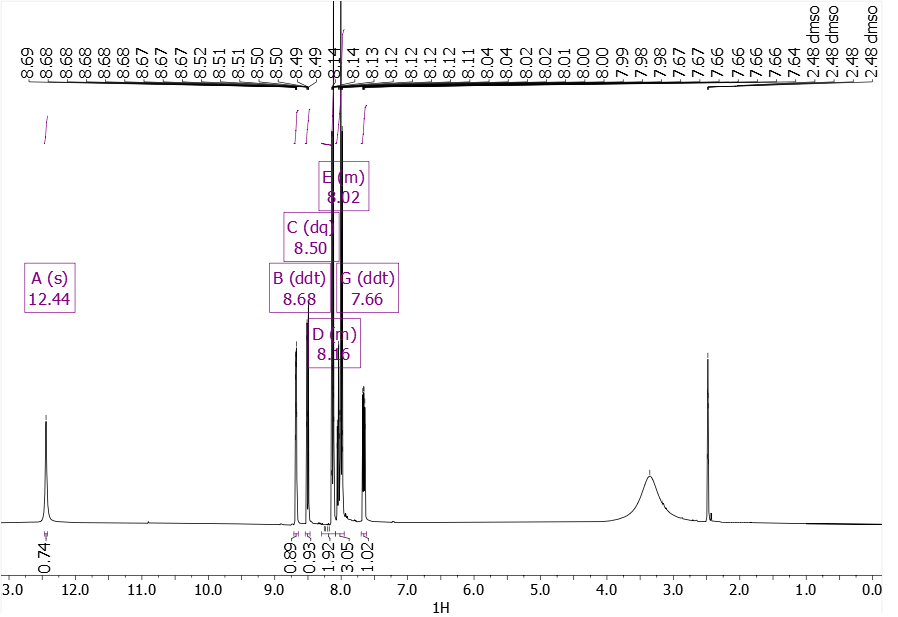


# Figure S1 ^1^H NMR spectrum of N-(4-benzoic acid)-2-pyridinecarbothioamide in DMSO-*d*_6_


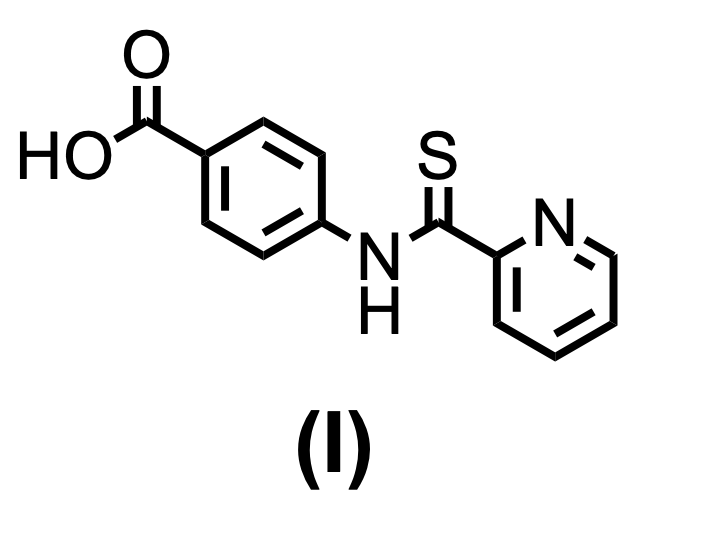

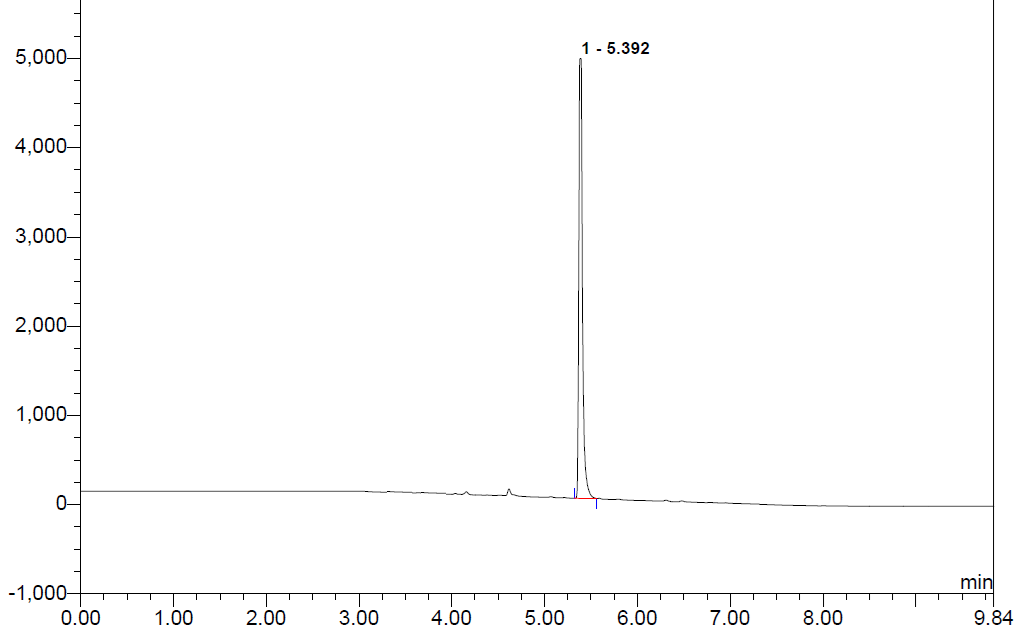


**Figure S2 HPLC chromatogram of N-(4-benzoic acid)-2-pyridinecarbothioamide –** 0-100% acetonitrile in 5.84 min + 4 min constant 100% acetonitrile.


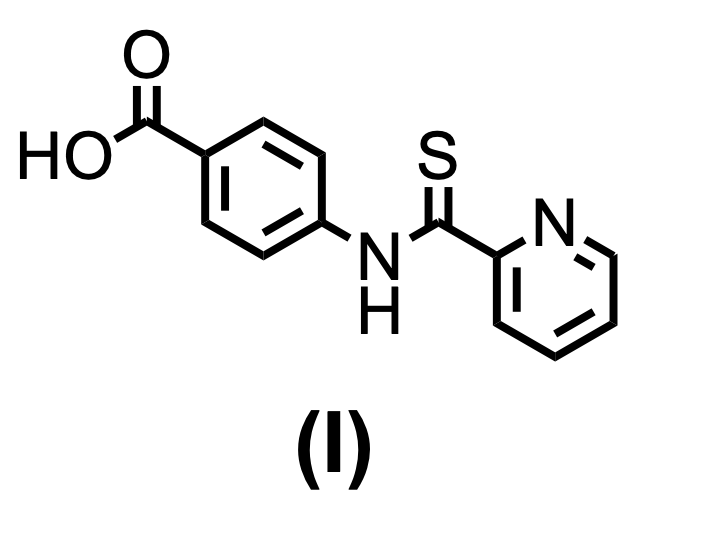


#


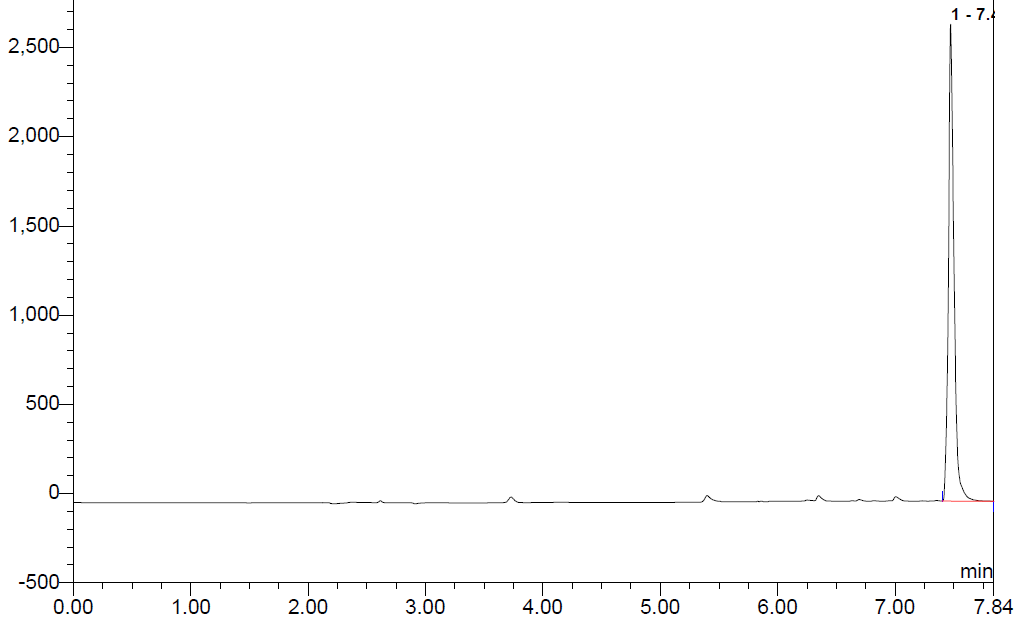


**Figure S3 HPLC chromatogram of N-(4-benzoic acid)-2-pyridinecarbothioamide anhydride –** 0-100% acetonitrile in 5.84 min + 2 min constant 100% acetonitrile.


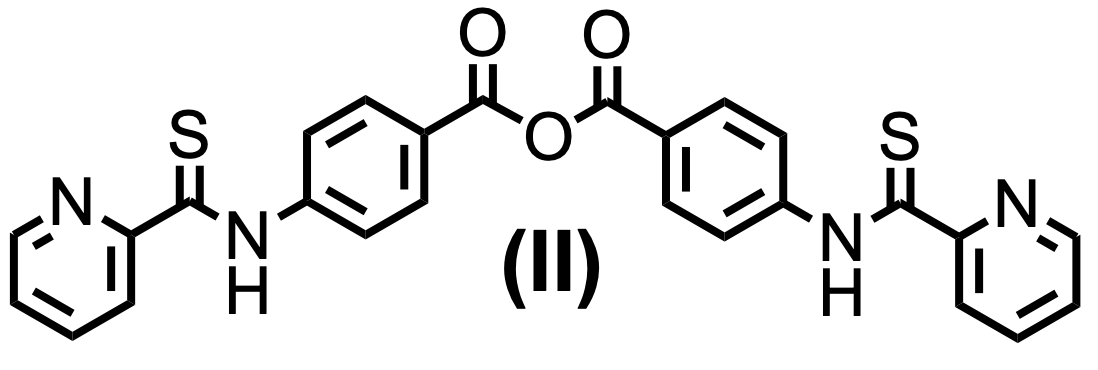


#

#


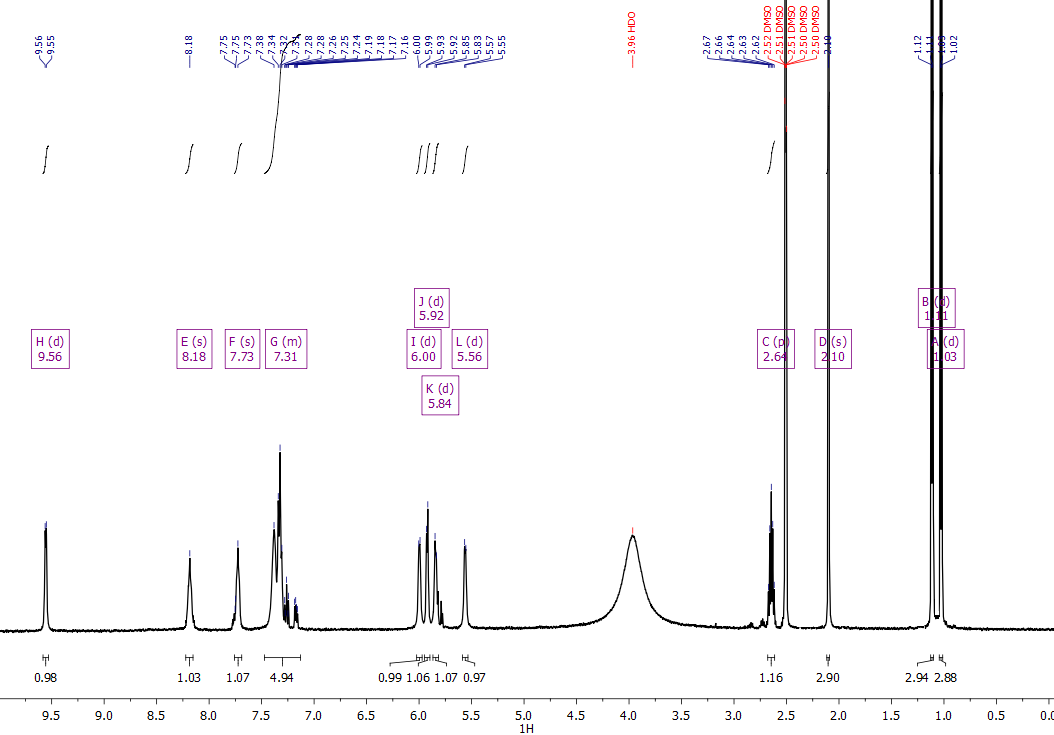


**Figure S4 ^1^H NMR spectrum of Ru^II^-F in DMSO-*d*_6_**


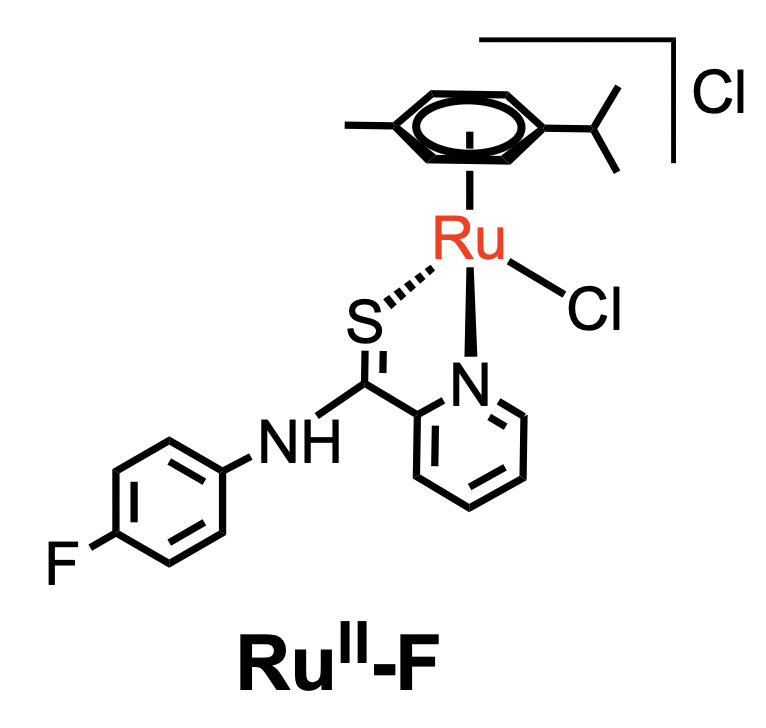

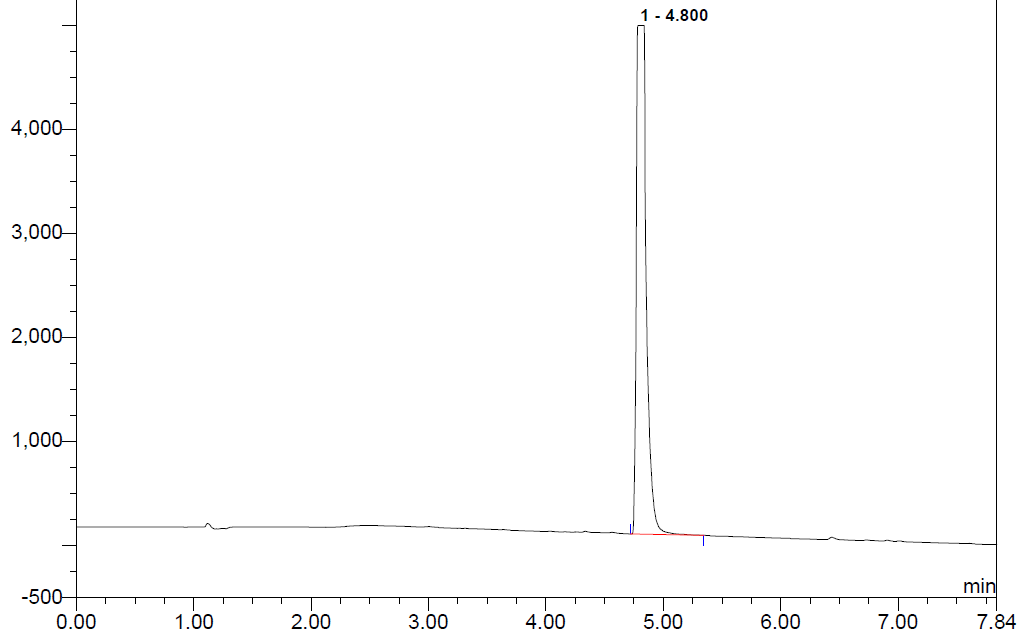


**Figure S5 HPLC chromatogram of Plecstatin-1 -** 0-100% acetonitrile in 5.84 min + 2 min constant 100% acetonitrile.


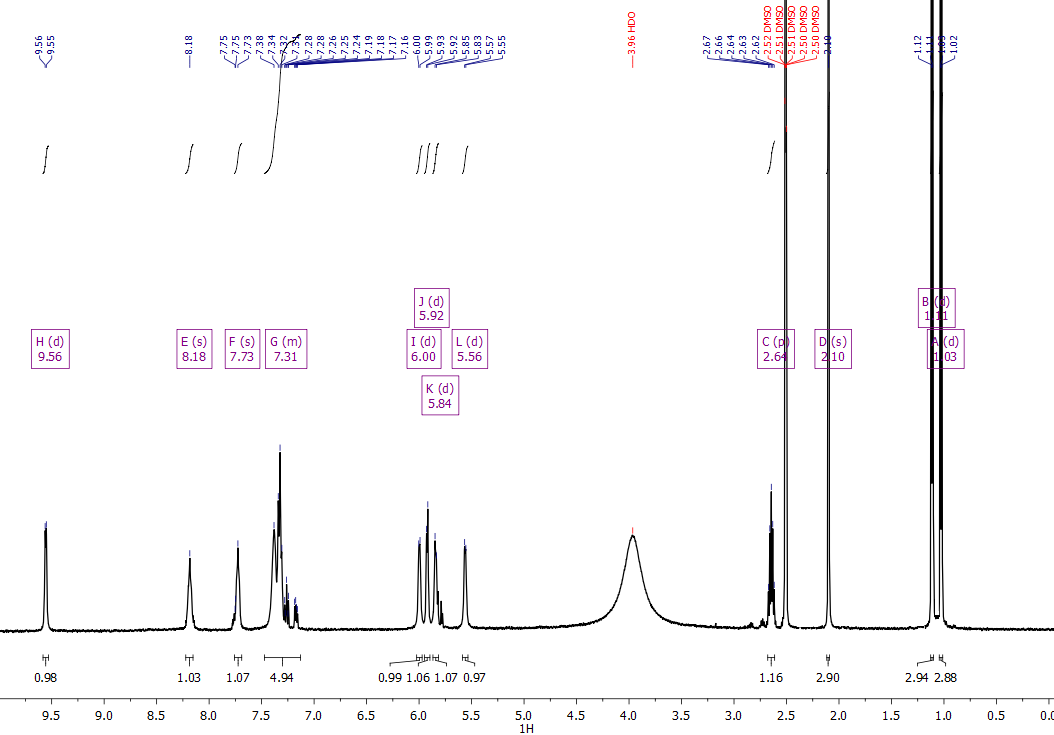

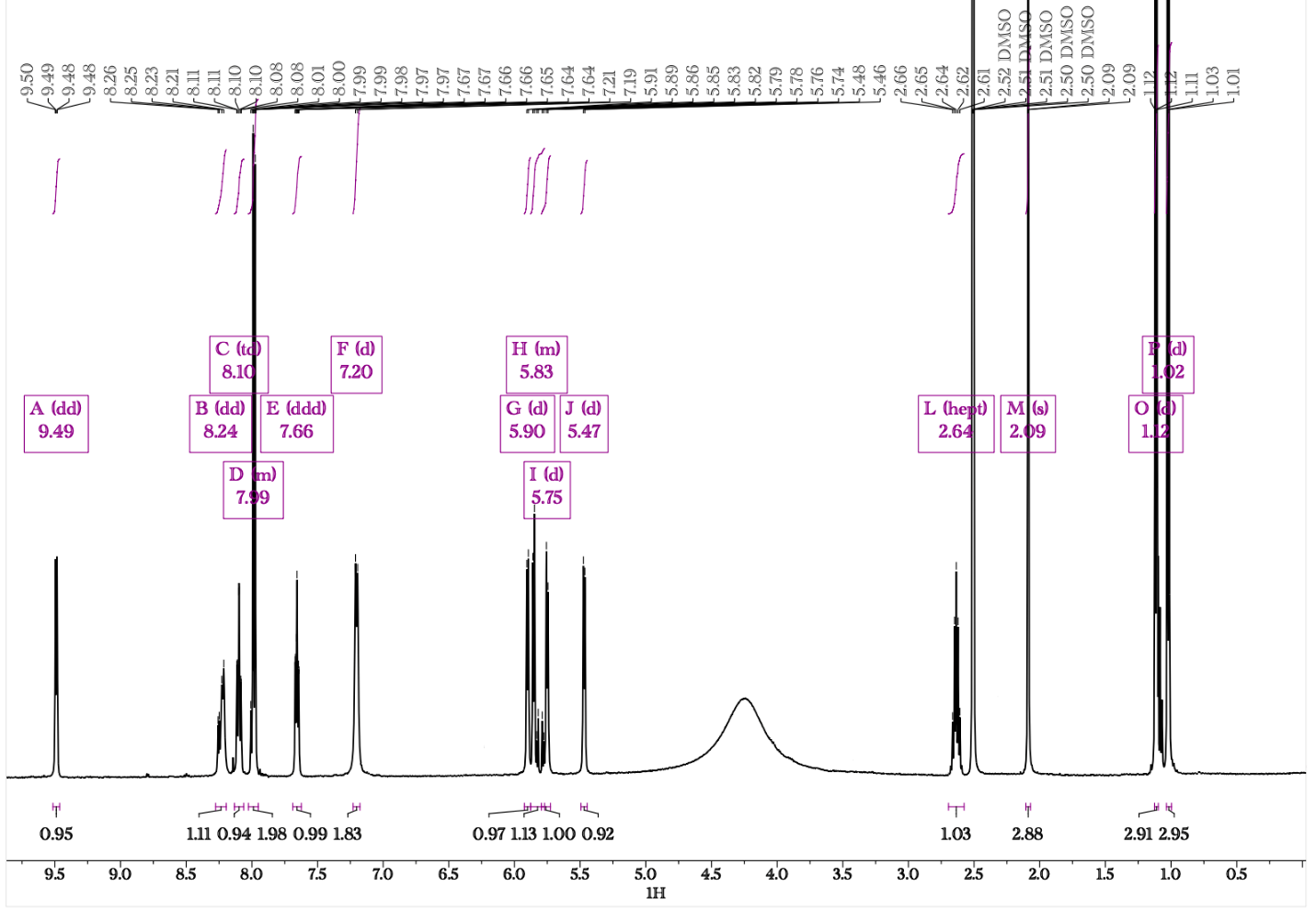


**Figure S6 ^1^H NMR spectrum of complex 1 in DMSO-*d*_6_**


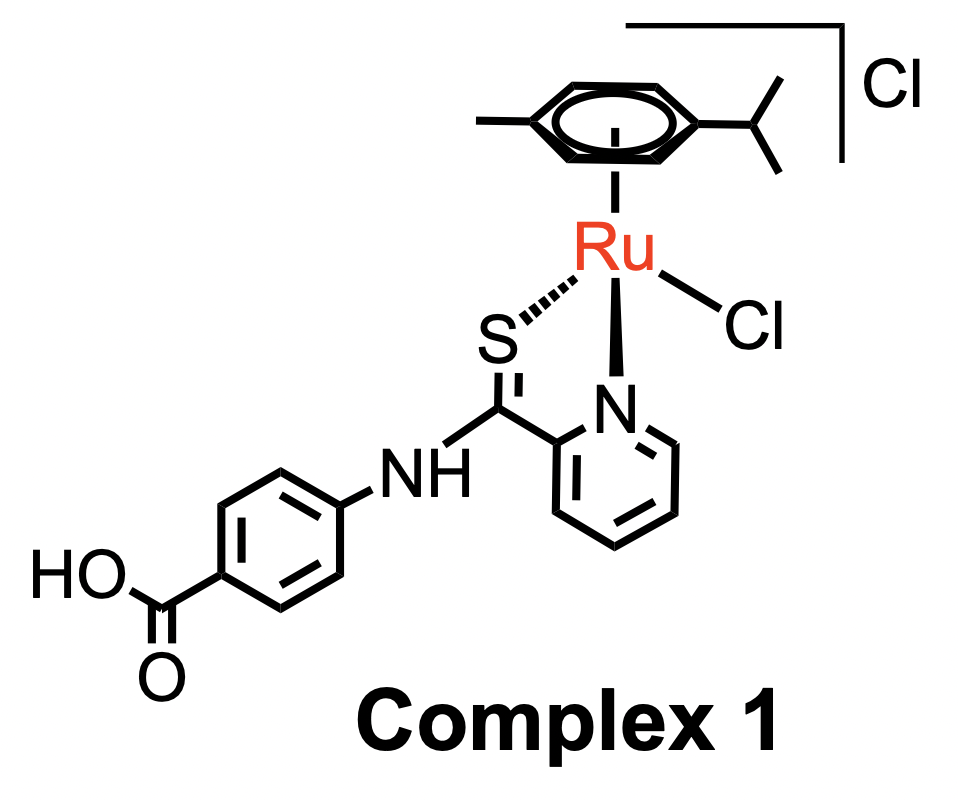

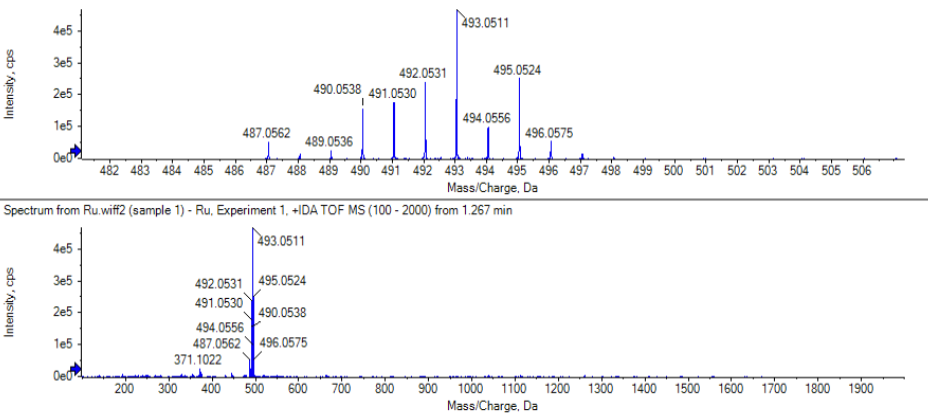

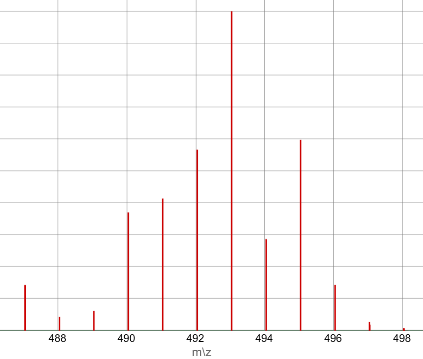


Calculated

**Figure S7 ESI-HRMS (+ve) of complex 1**


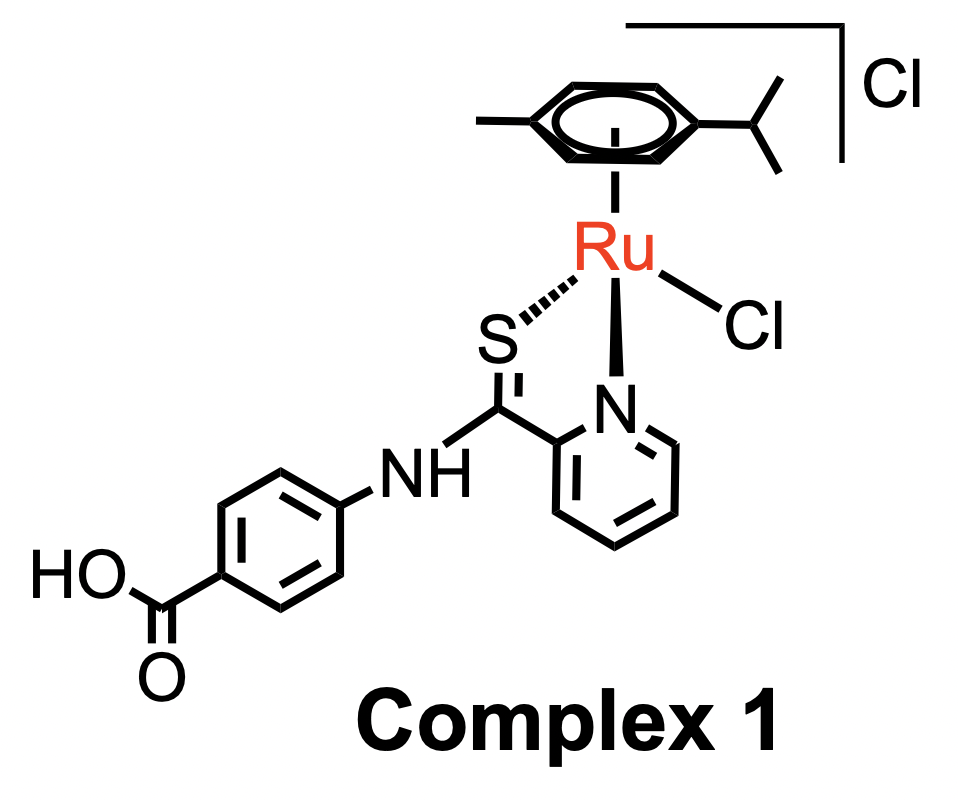

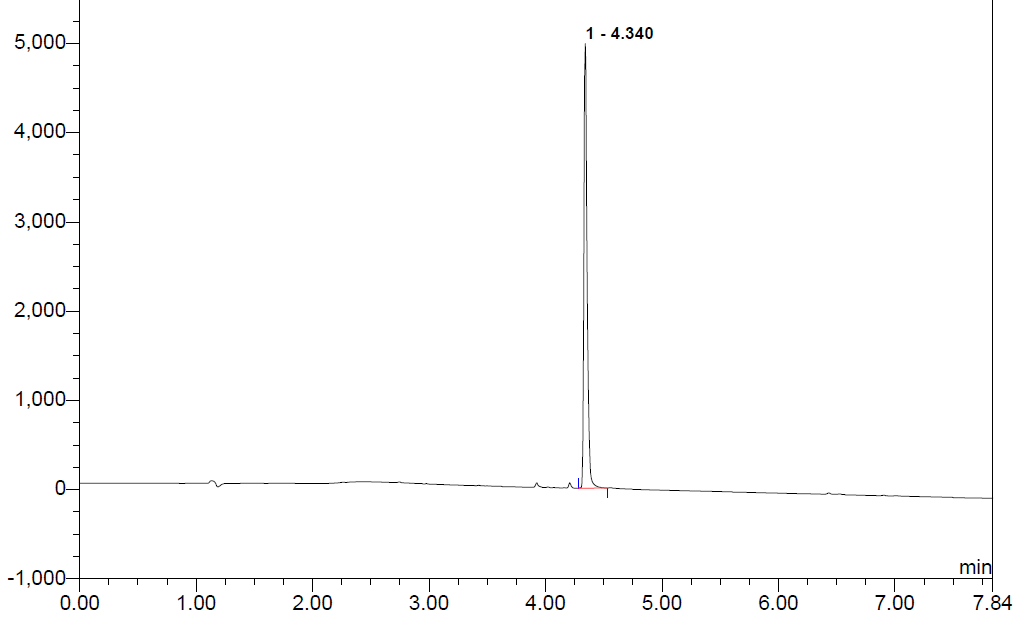


**Figure S8 HPLC chromatogram of complex 1 -** 0-100% acetonitrile in 5.84 min + 2 min constant 100% acetonitrile.


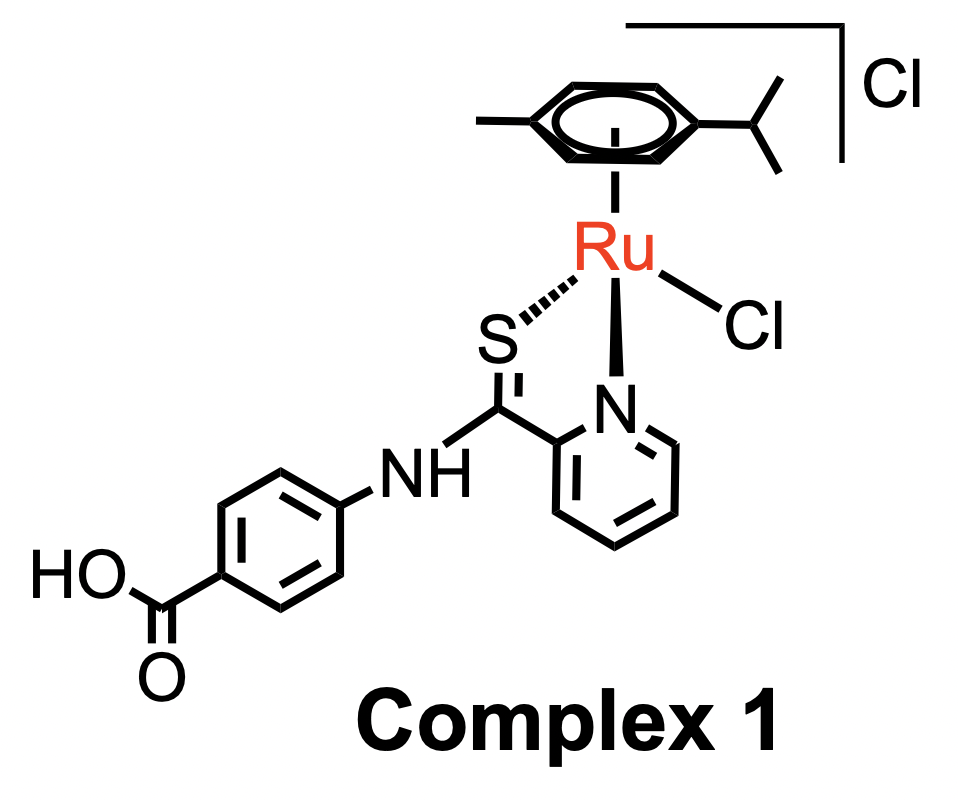

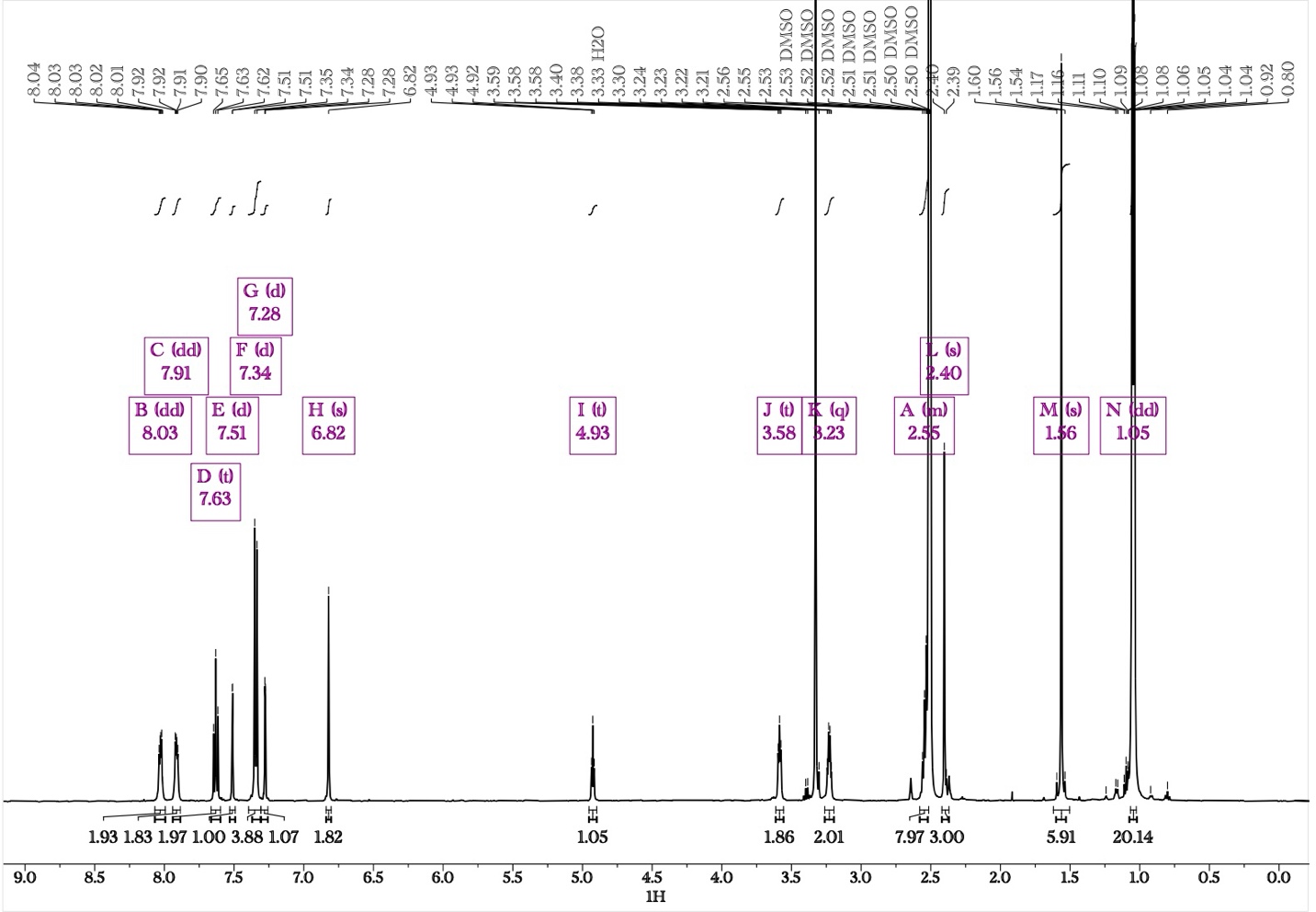


**Figure S9 ^1^H NMR spectrum of (1,3-bis(2,6-diisopropylphenyl)-4,9-dioxo-1,3,4,9-tetrahydro-2*H*-2λ3-naphtho[2,3-*d*]imidazol-2-yl)(1-(2-hydroxyethyl)-3-mesityl-1,3-dihydro-2*H*-2λ3-imidazol-2-yl) gold(I) in DMSO-*d*_6_**


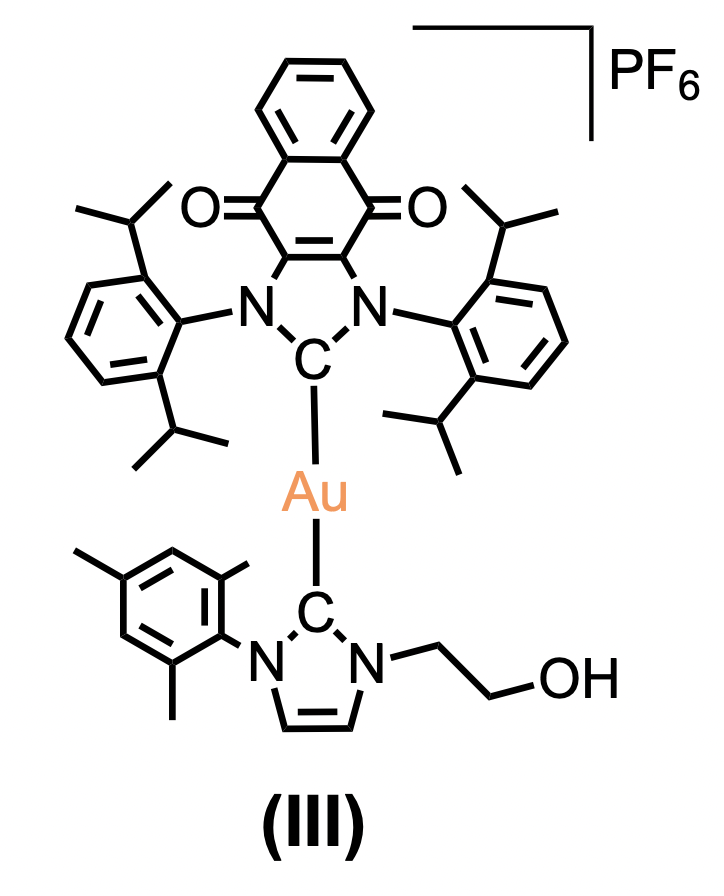

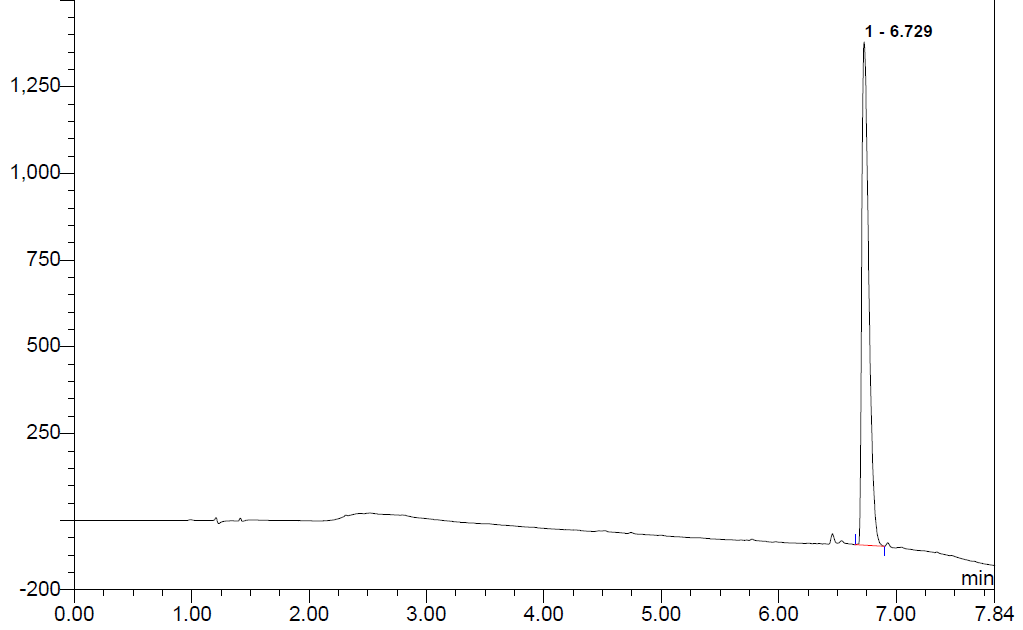


**Figure S10 HPLC chromatogram of (1,3-bis(2,6-diisopropylphenyl)-4,9-dioxo-1,3,4,9-tetrahydro-2*H*-2λ3-naphtho[2,3-*d*]imidazol-2-yl)(1-(2-hydroxyethyl)-3-mesityl-1,3-dihydro-2*H*-2λ3-imidazol-2-yl) gold(I) -** 0-100% acetonitrile in 5.84 min + 2 min constant 100% acetonitrile.


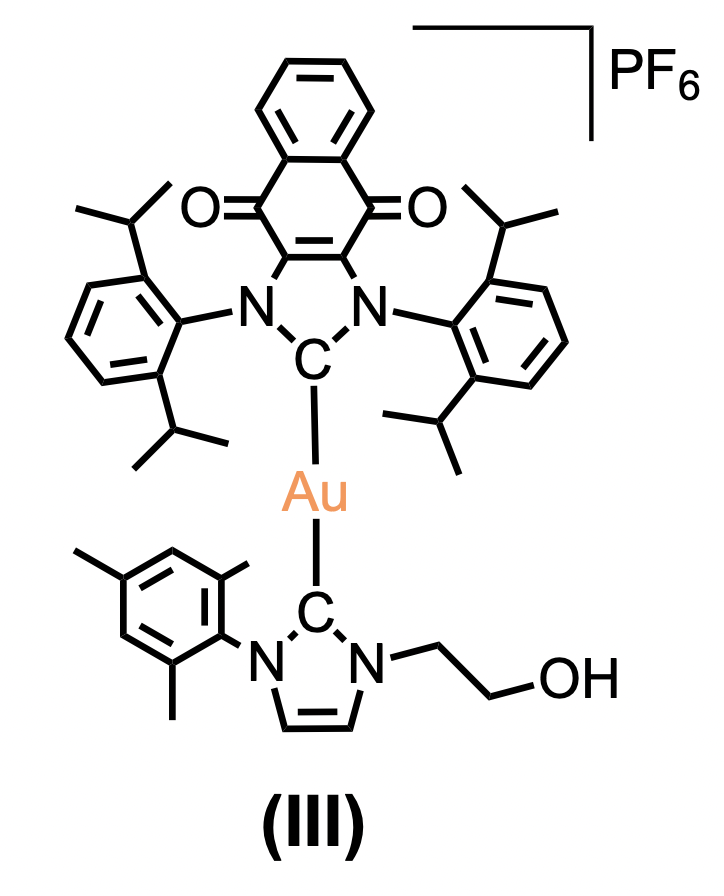

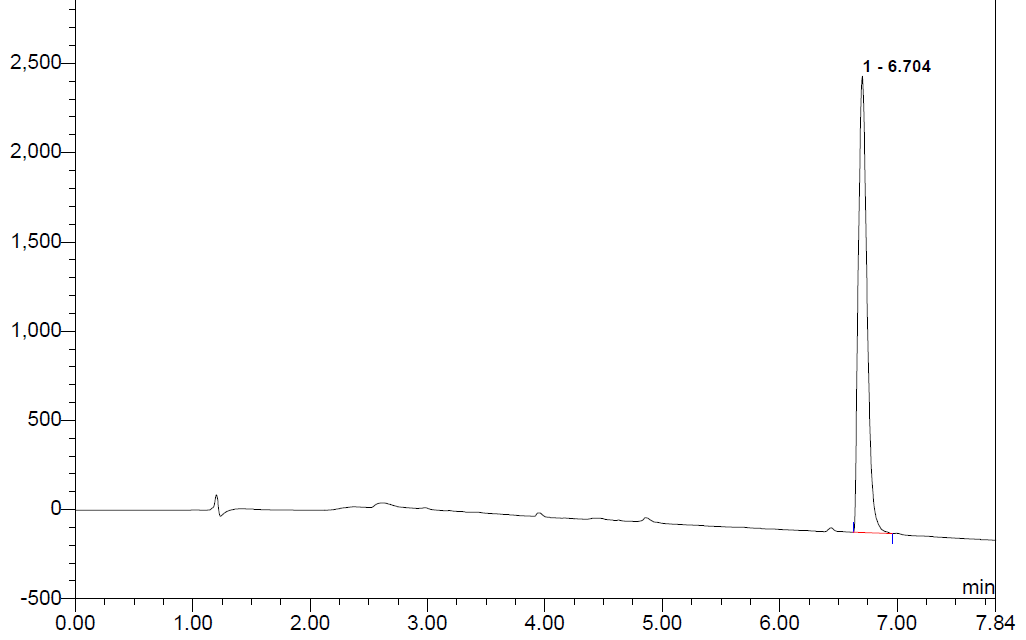


**Figure S11 HPLC chromatogram of (1,3-bis(2,6-diisopropylphenyl)-4,9-dioxo-1,3,4,9-tetrahydro-2*H*-2λ3-naphtho[2,3-*d*]imidazol-2-yl)(1-(2-((((2,5-dioxopyrrolidin-1-yl)oxy)carbonyl)oxy)ethyl)-3-mesityl-1,3-dihydro-2*H*-2λ3-imidazol-2-yl) gold(I) -** 0-100% acetonitrile in 5.84 min + 2 min constant 100% acetonitrile.


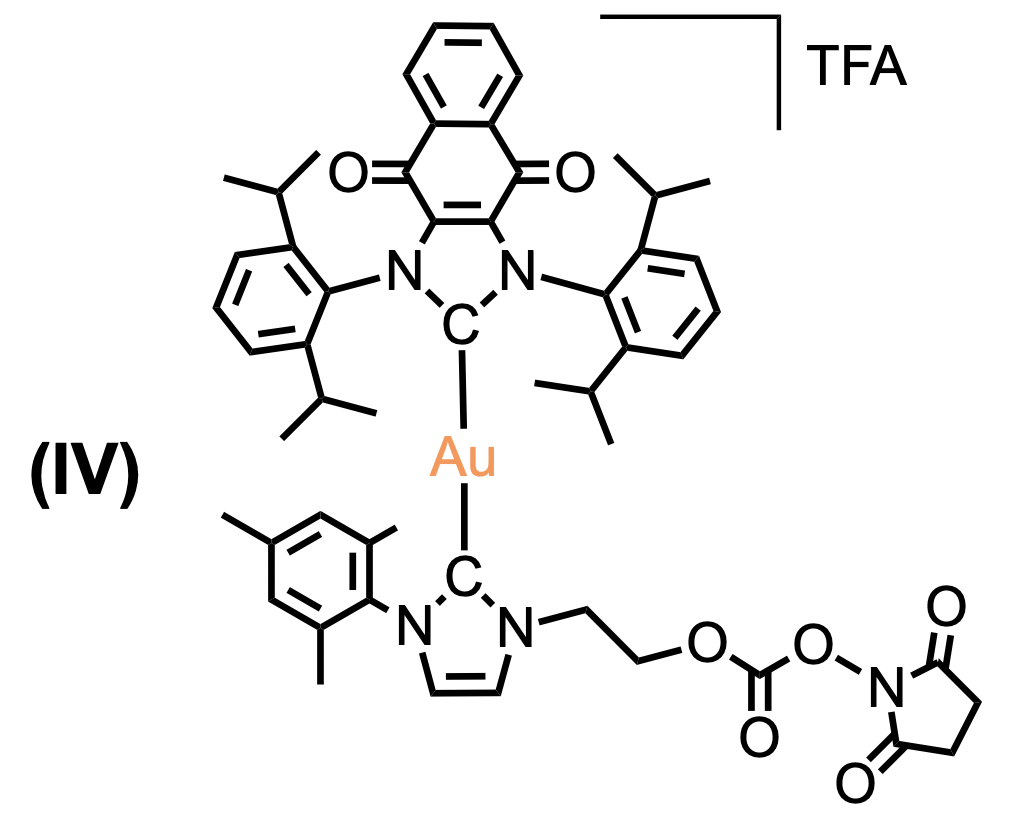

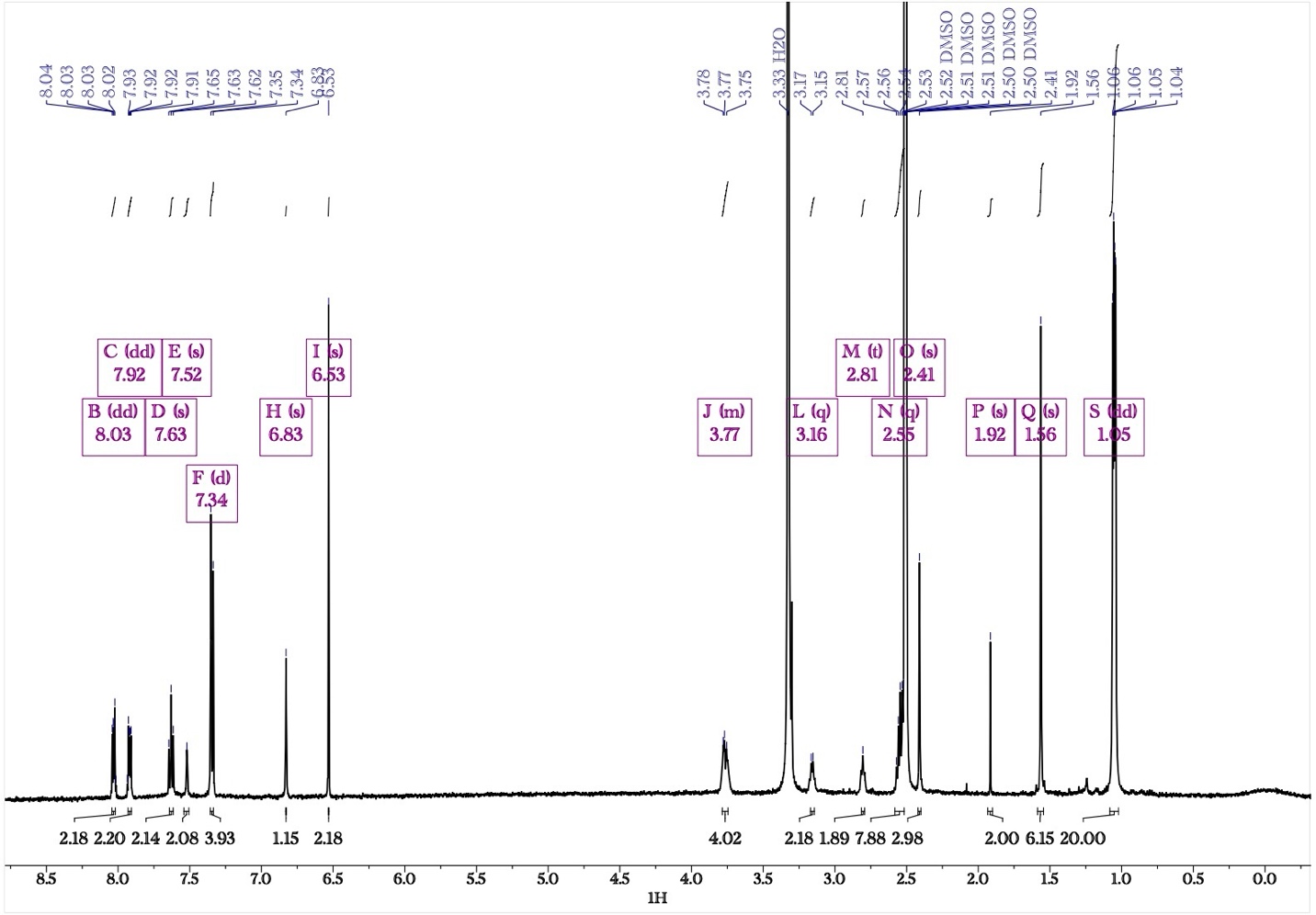


**Figure S12 ^1^H NMR spectrum of complex 2 in DMSO-*d*_6_**


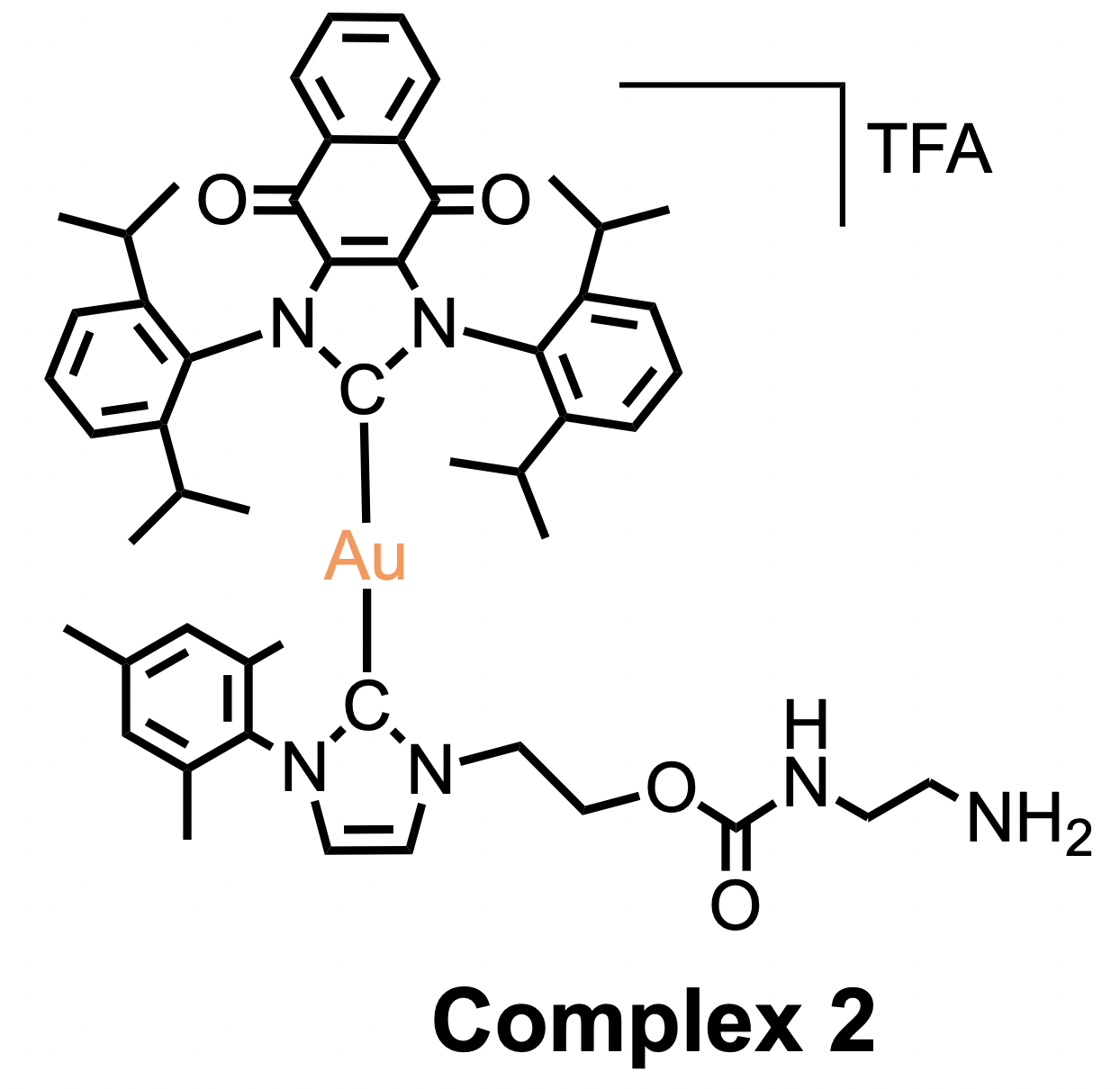

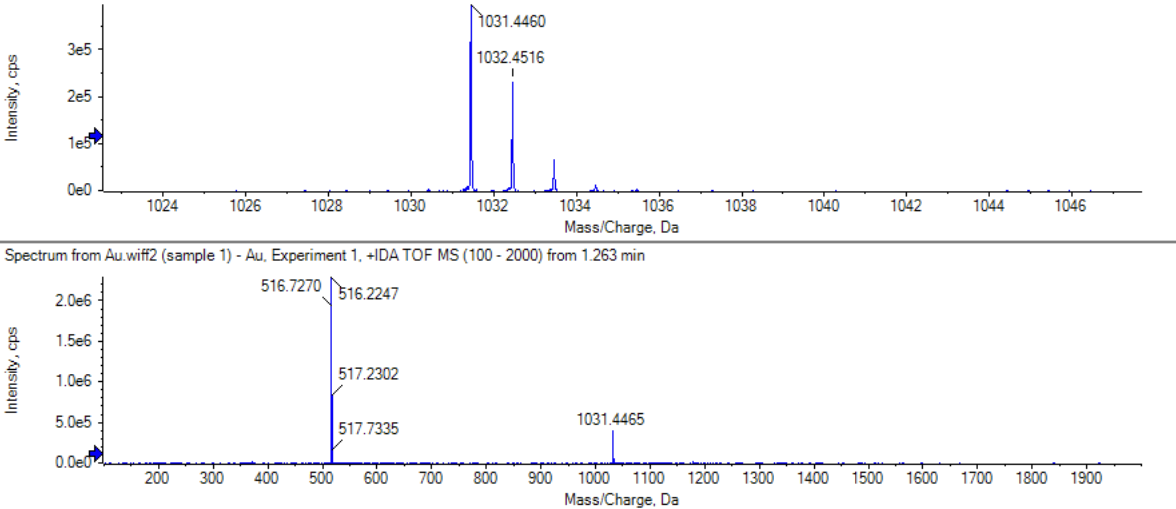

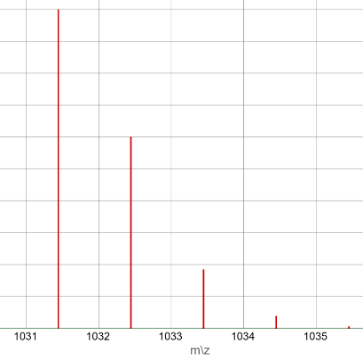


Calculated

**Figure S13 ESI-HRMS (+ve) of complex 2**


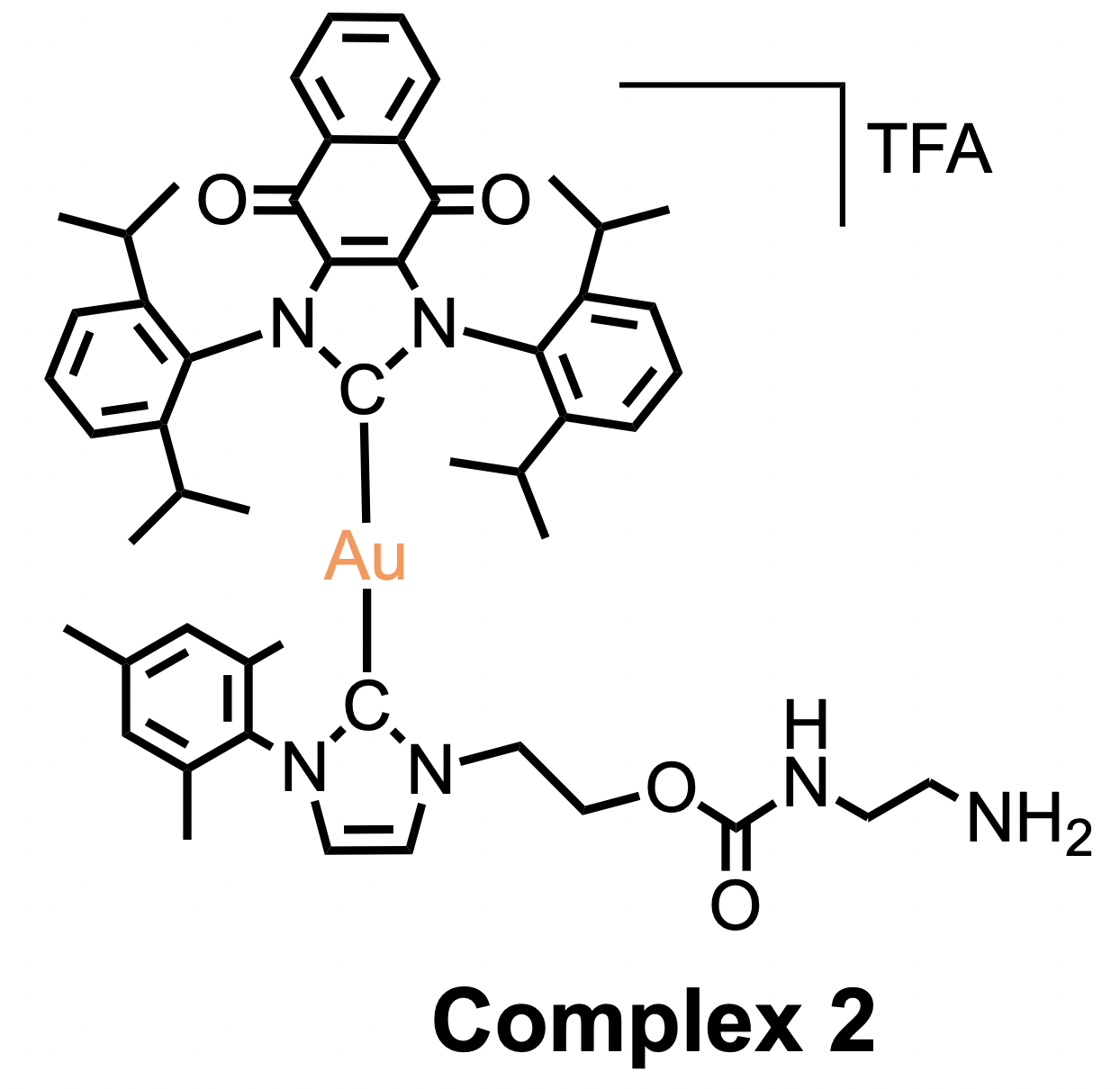

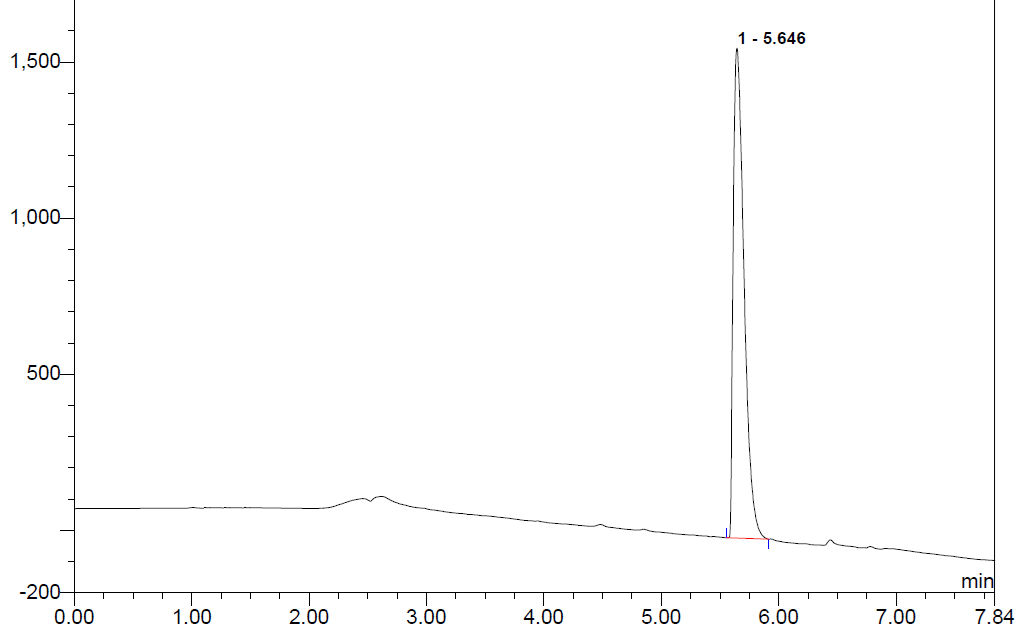


**Figure S14 HPLC chromatogram of complex 2 -** 0-100% acetonitrile in 5.84 min + 2 min constant 100% acetonitrile.


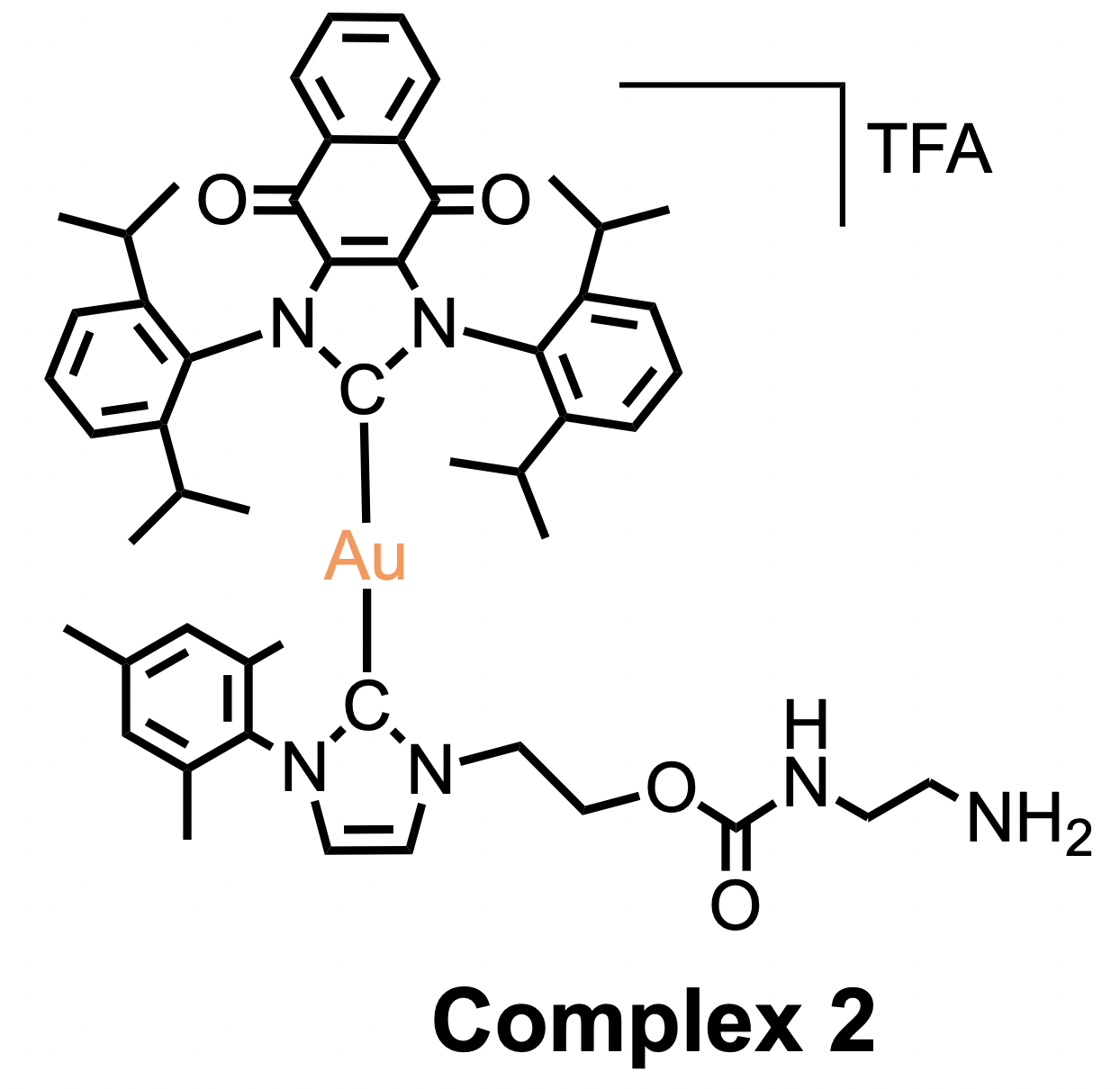

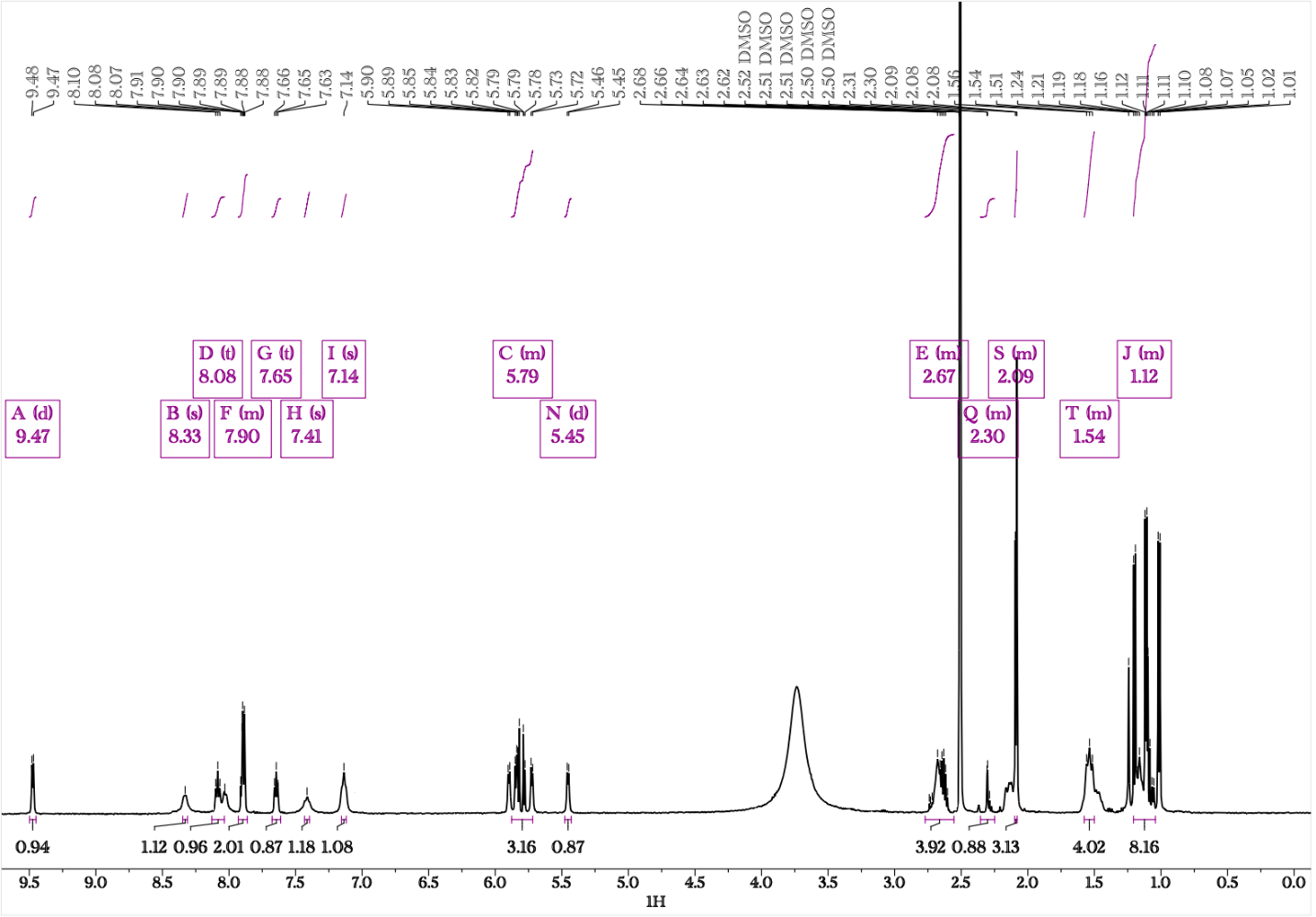


**Figure S15 ^1^H NMR spectrum of complex 3 in DMSO-*d*_6_**


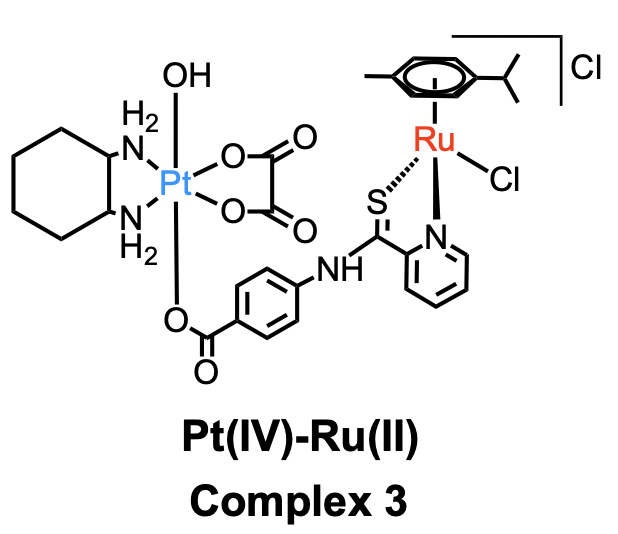

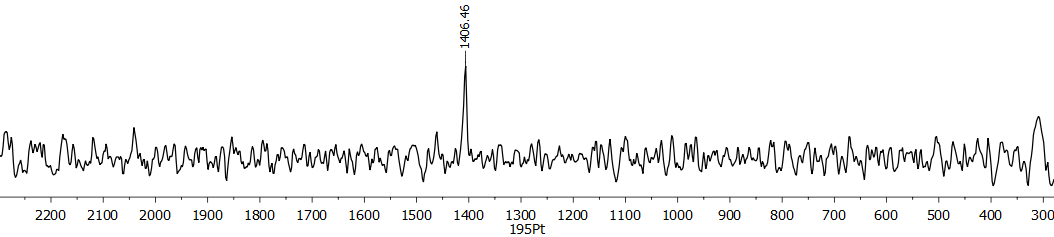


**Figure S16 ^195^Pt NMR spectrum of complex 3 in DMSO-*d_6_***


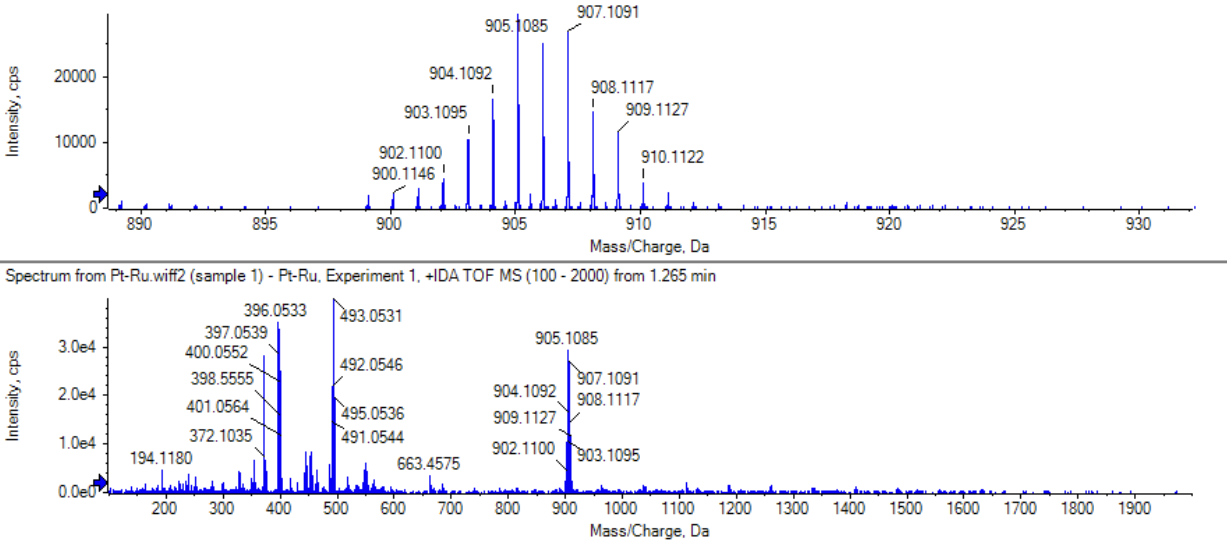

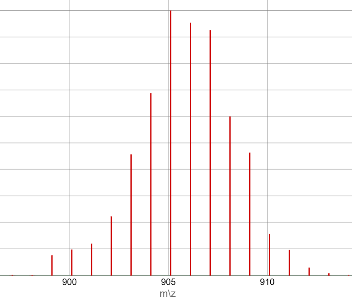


Calculated

**Figure S17 ESI-HRMS (+ve) of complex 3**


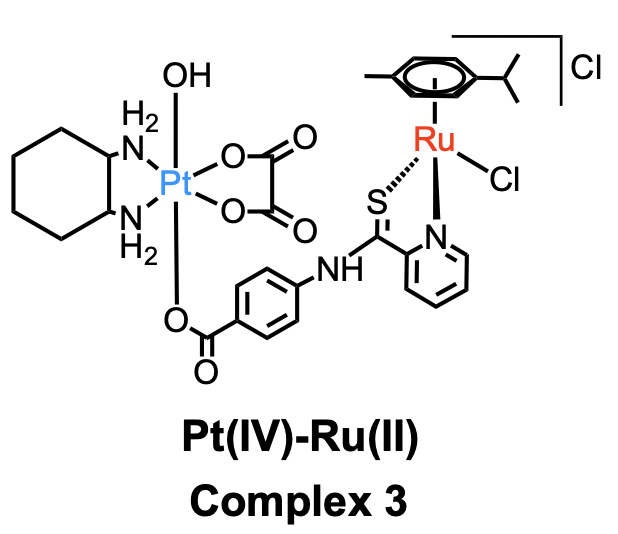

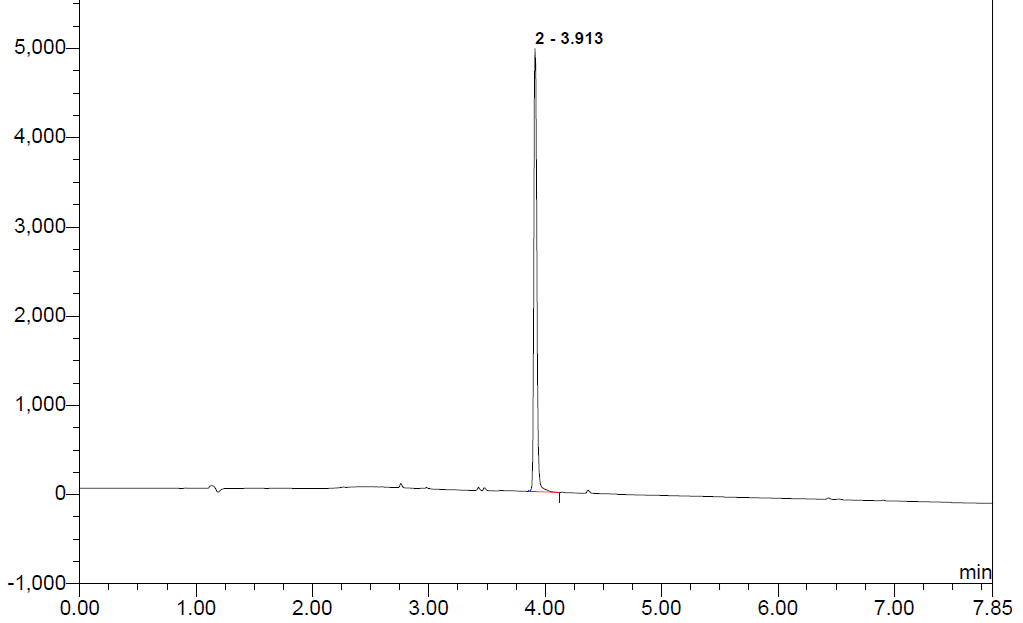


**Figure S18 HPLC Chromatogram of complex 3 -** 0-100% acetonitrile in 5.84 min + 2 min constant 100% acetonitrile.


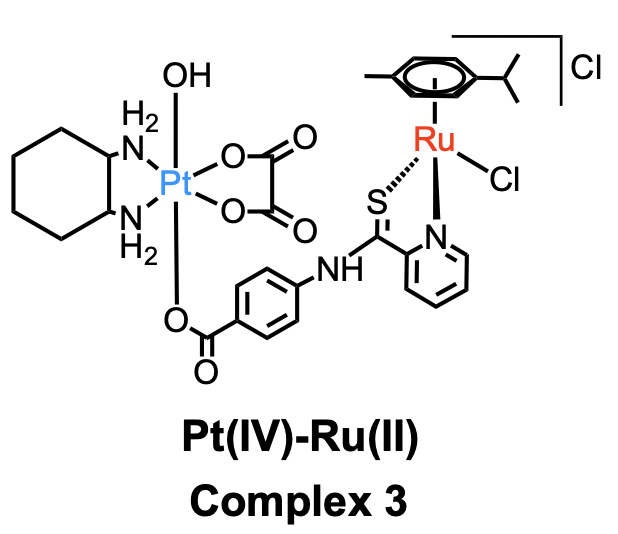

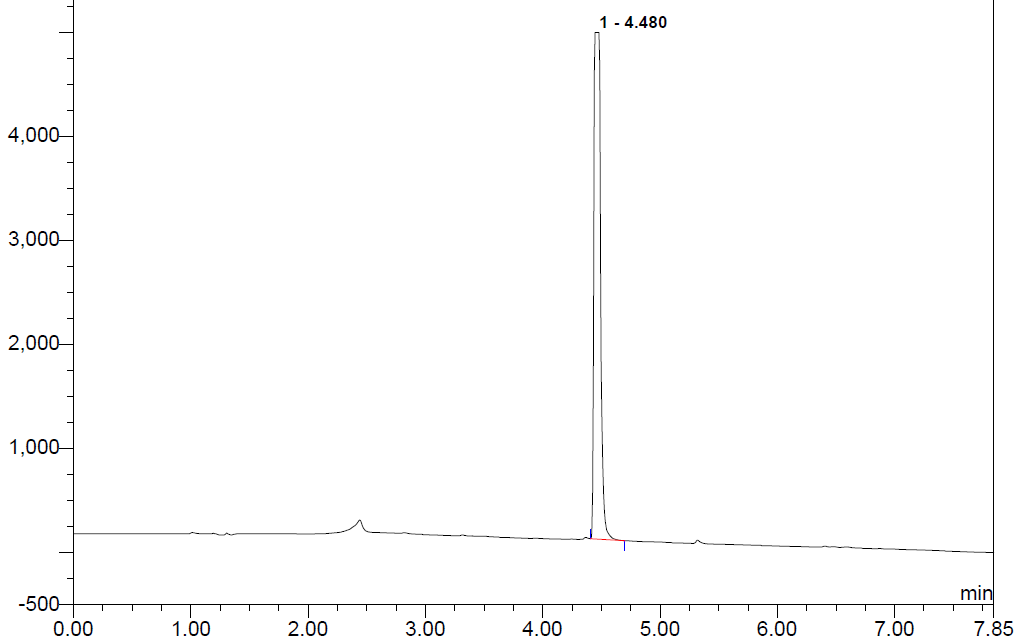


**Figure S19 HPLC chromatogram of oxali(N-(4-benzoic acid)-2-pyridinecarbothioamide)(OH)-** 0-100% acetonitrile in 5.84 min + 2 min constant 100% acetonitrile.


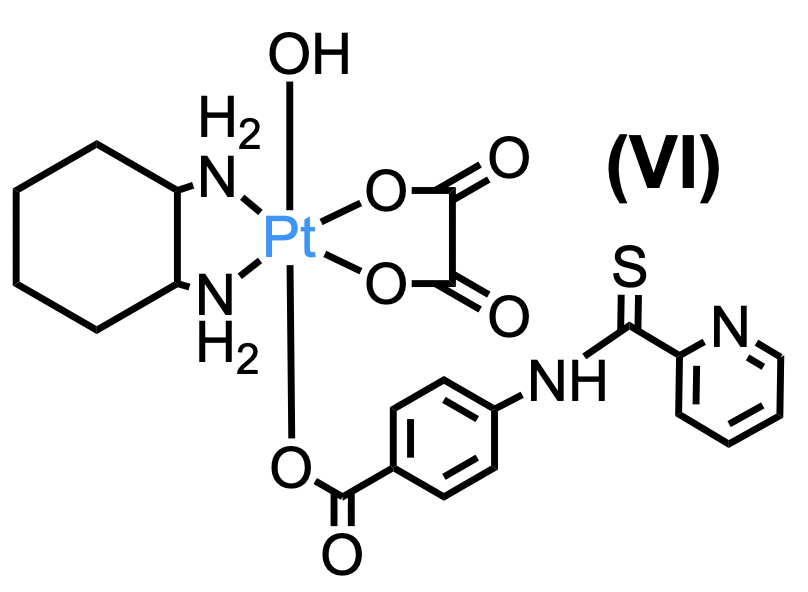

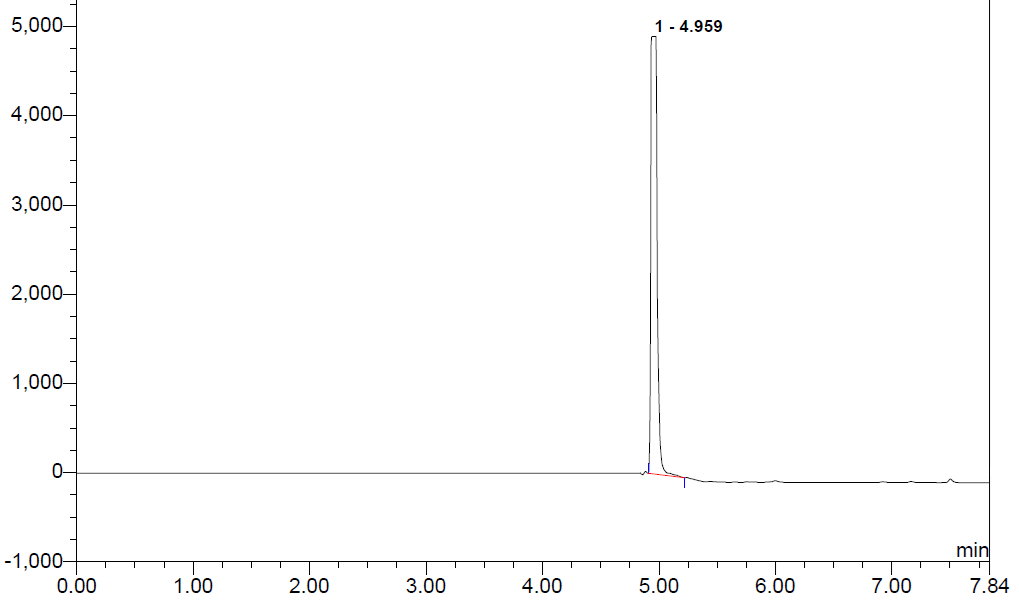


**Figure S20 HPLC chromatogram of oxali(N-(4-benzoic acid)-2-pyridinecarbothioamide)(MSC) -** 0-100% acetonitrile in 5.84 min + 2 min constant 100% acetonitrile.


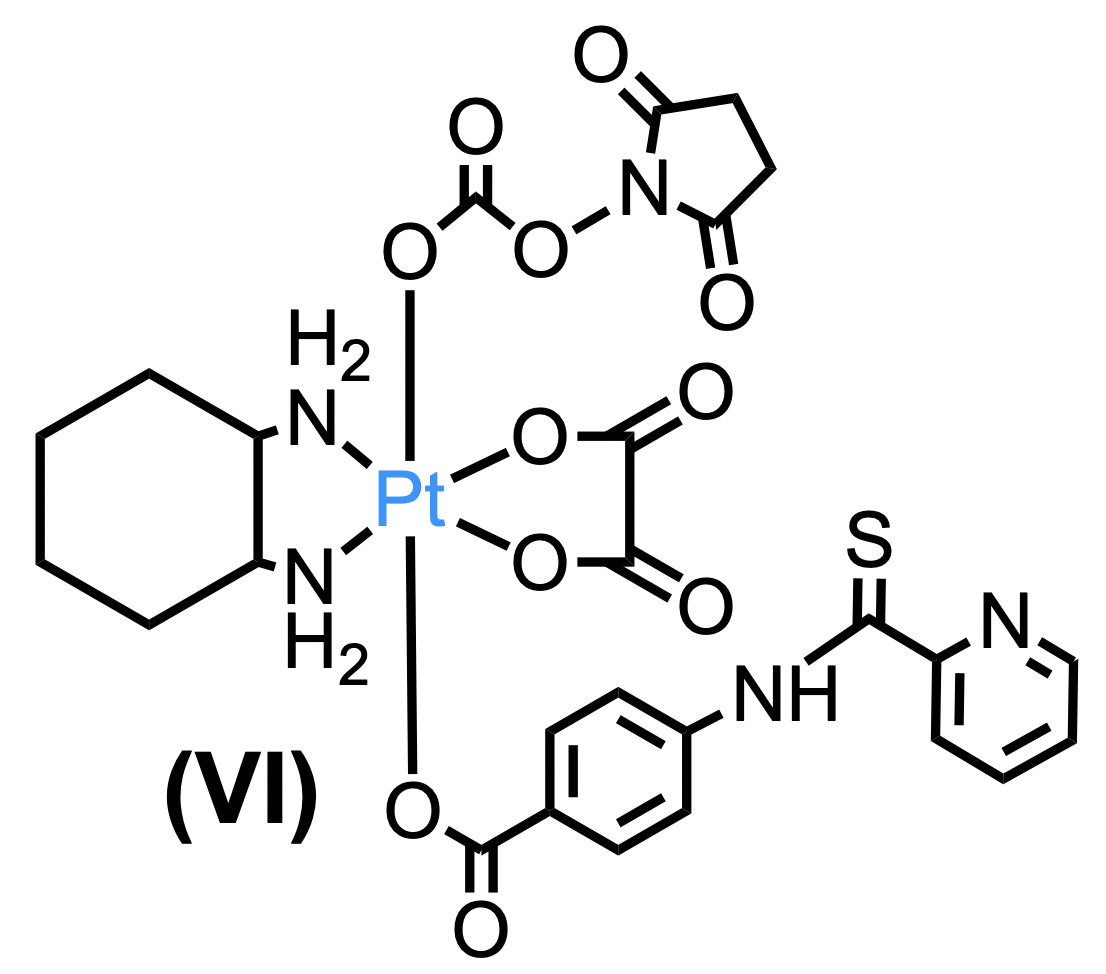

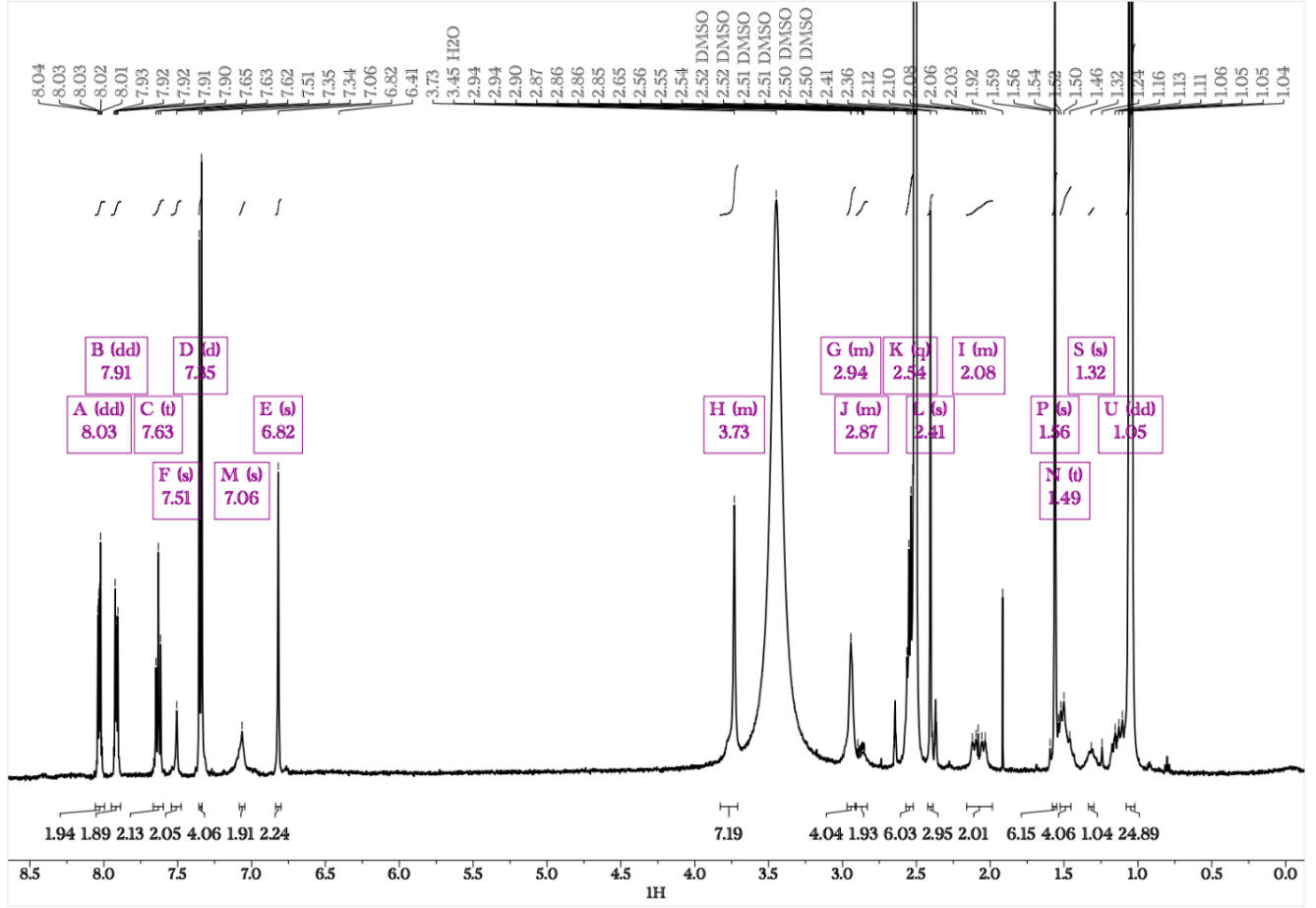


**Figure S21 ^1^H NMR spectrum of complex 4 in DMSO-*d*_6_**


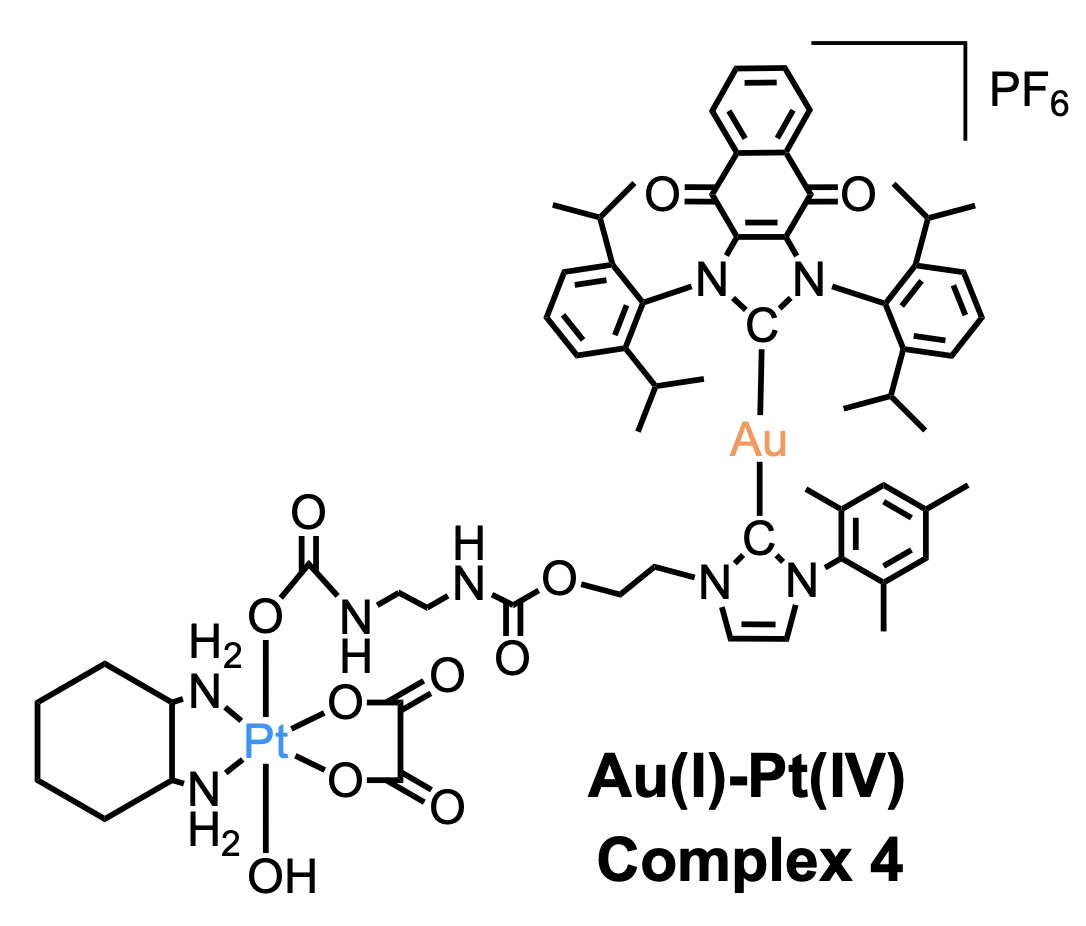

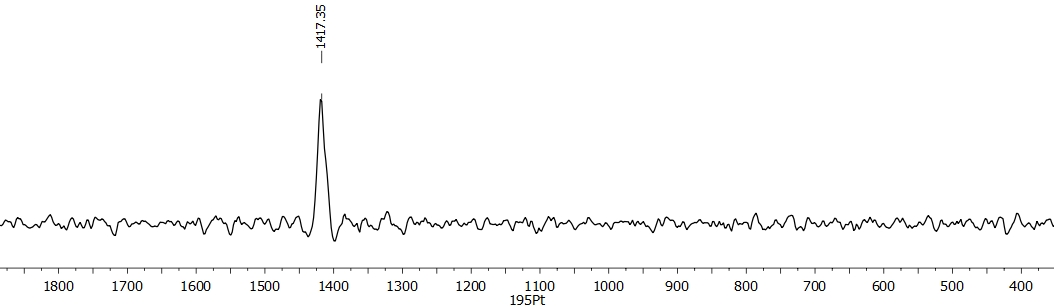


**Figure S22 ^195^Pt NMR spectrum of complex 4 in DMSO-*d_6_***

#


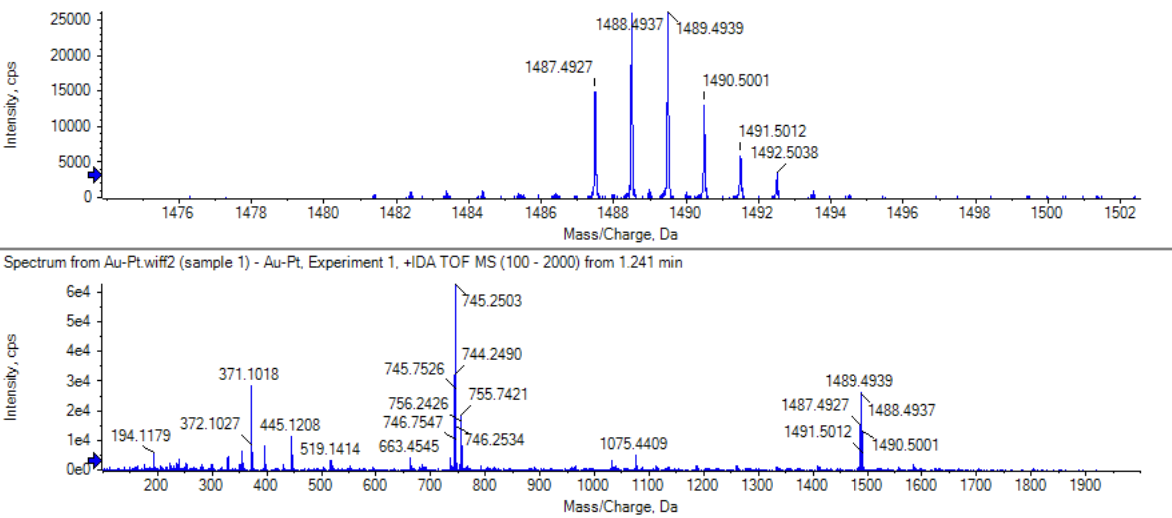

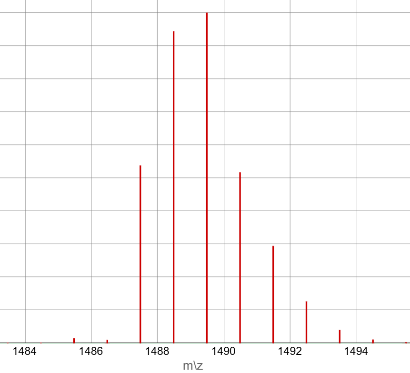


Calculated

**Figure S23 ESI-HRMS (+ve) of complex 4**


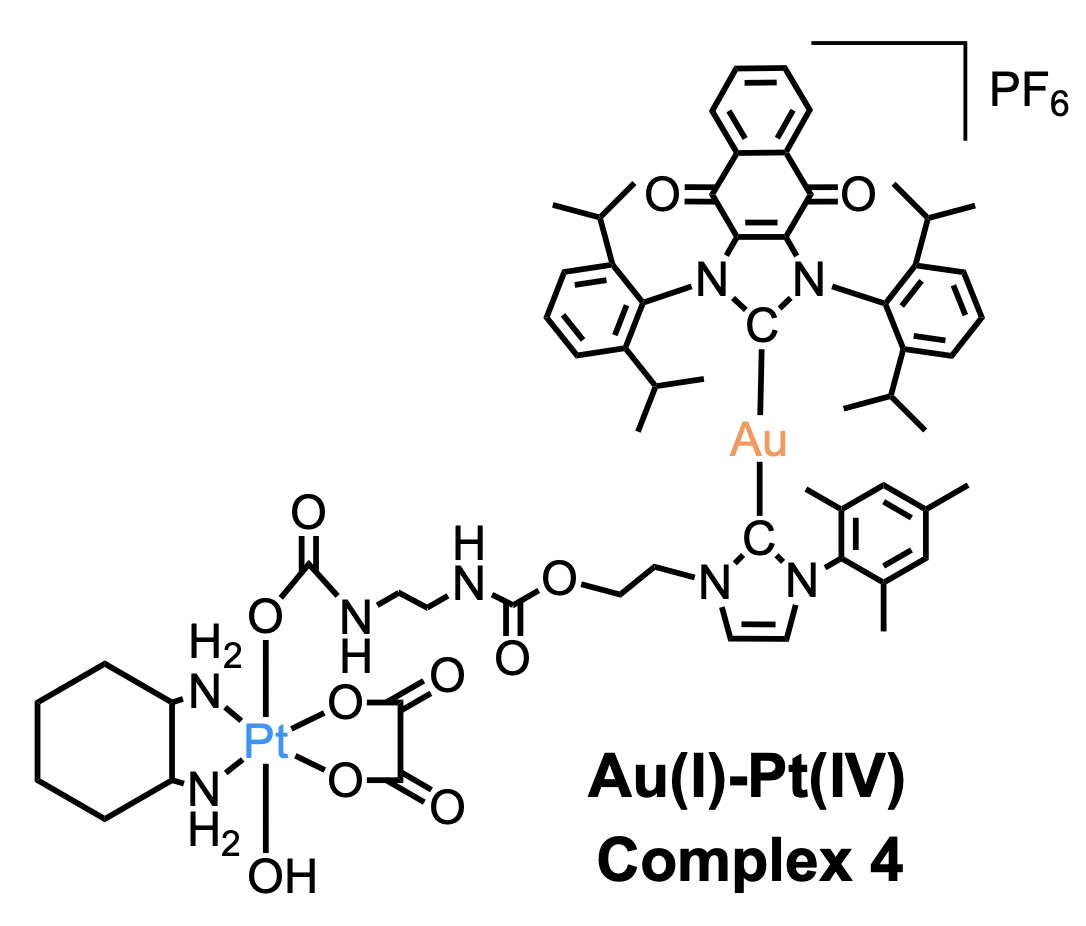

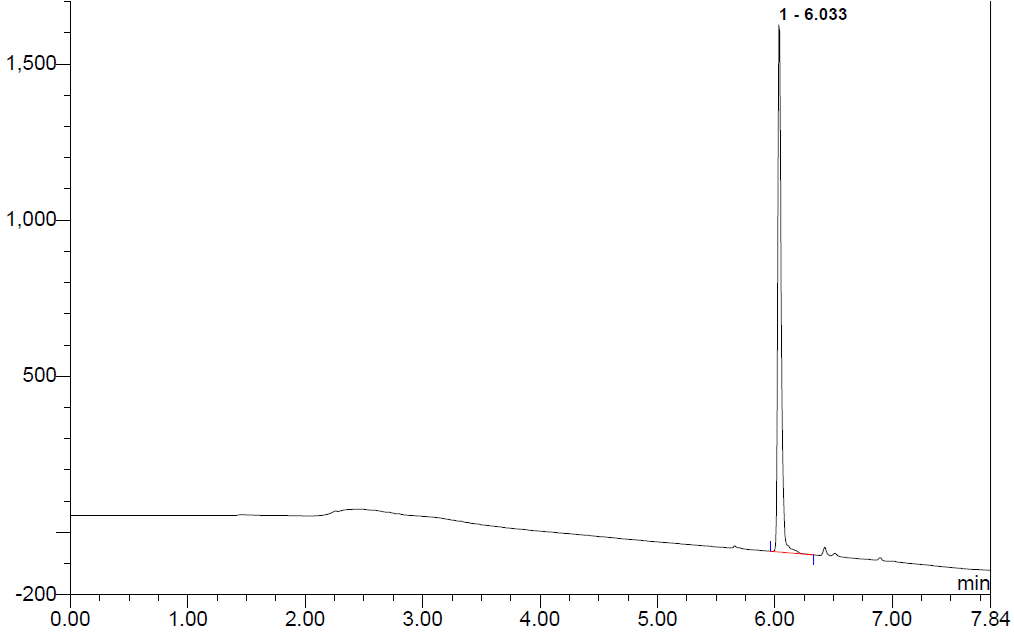


**Figure S24 HPLC chromatogram of complex 4 -** 0-100% acetonitrile in 5.84 min + 2 min constant 100% acetonitrile.


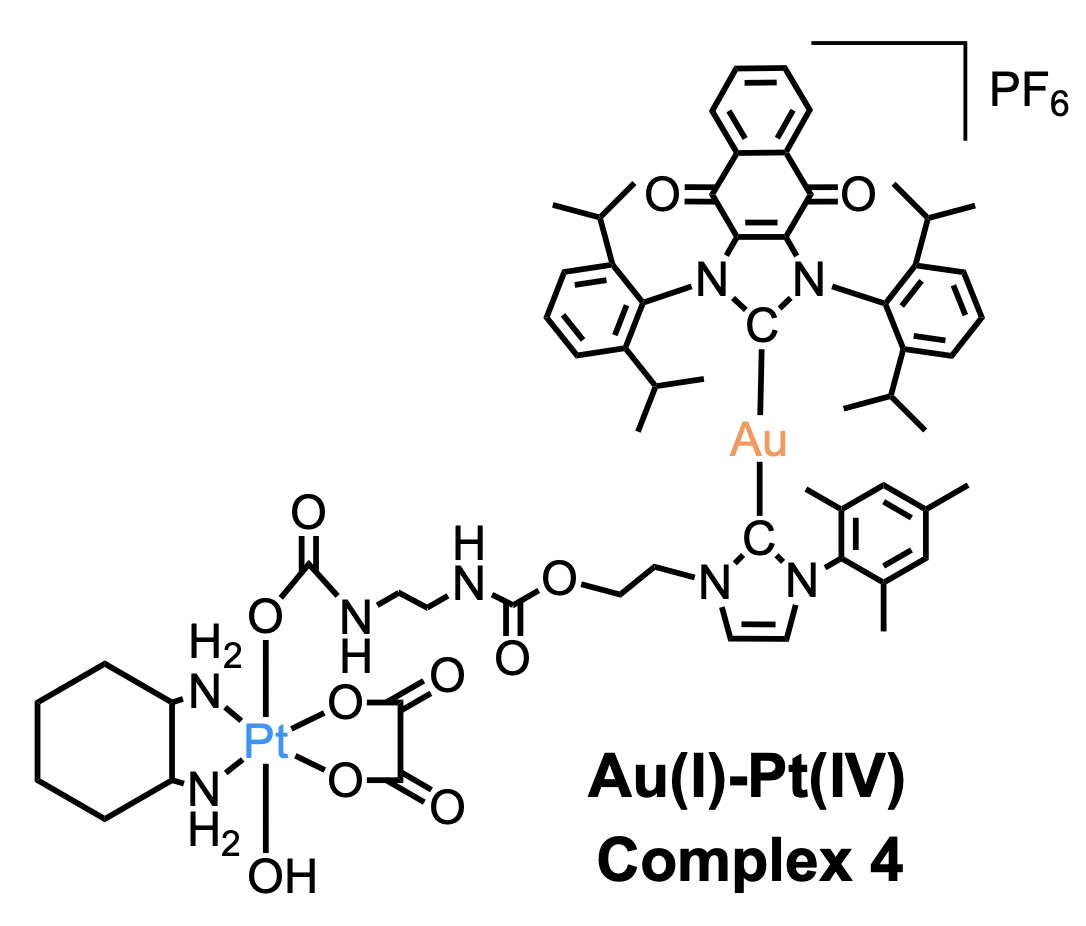


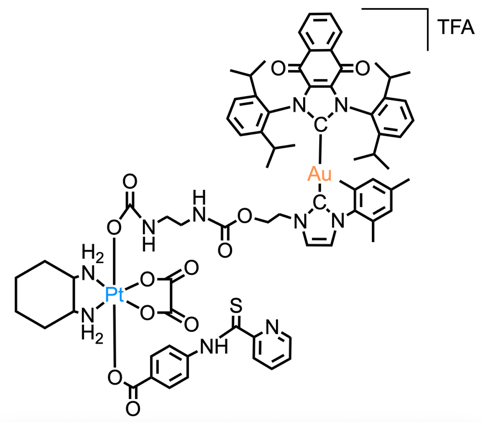


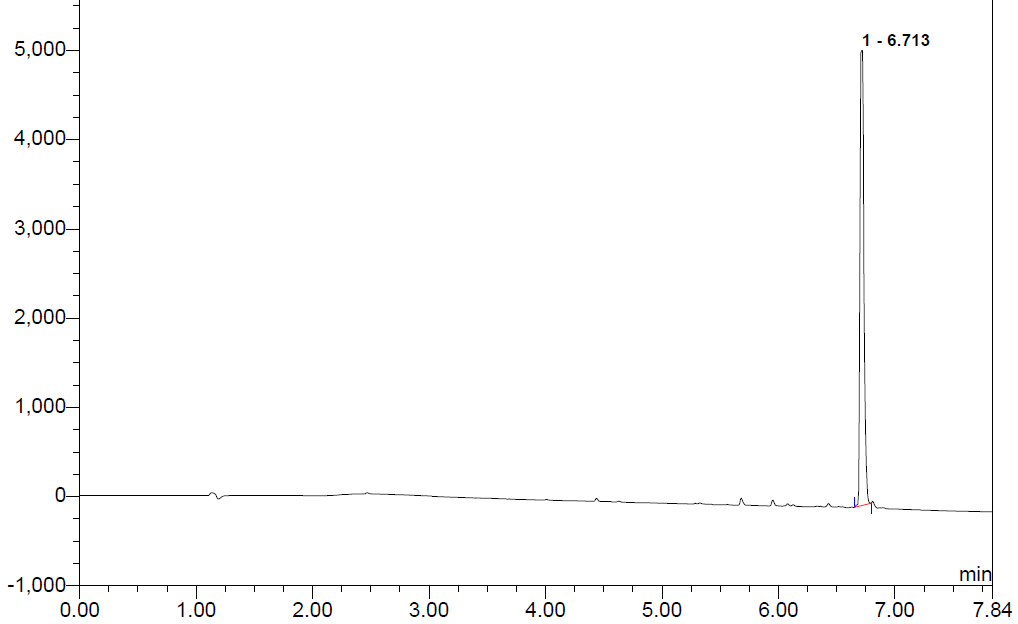


**Figure S25 HPLC chromatogram of oxali(N-(4-benzoic acid)-2-pyridinecarbothioamide) (Au(I)-Amine) -** 0-100% acetonitrile in 5.84 min + 2 min constant 100% acetonitrile.


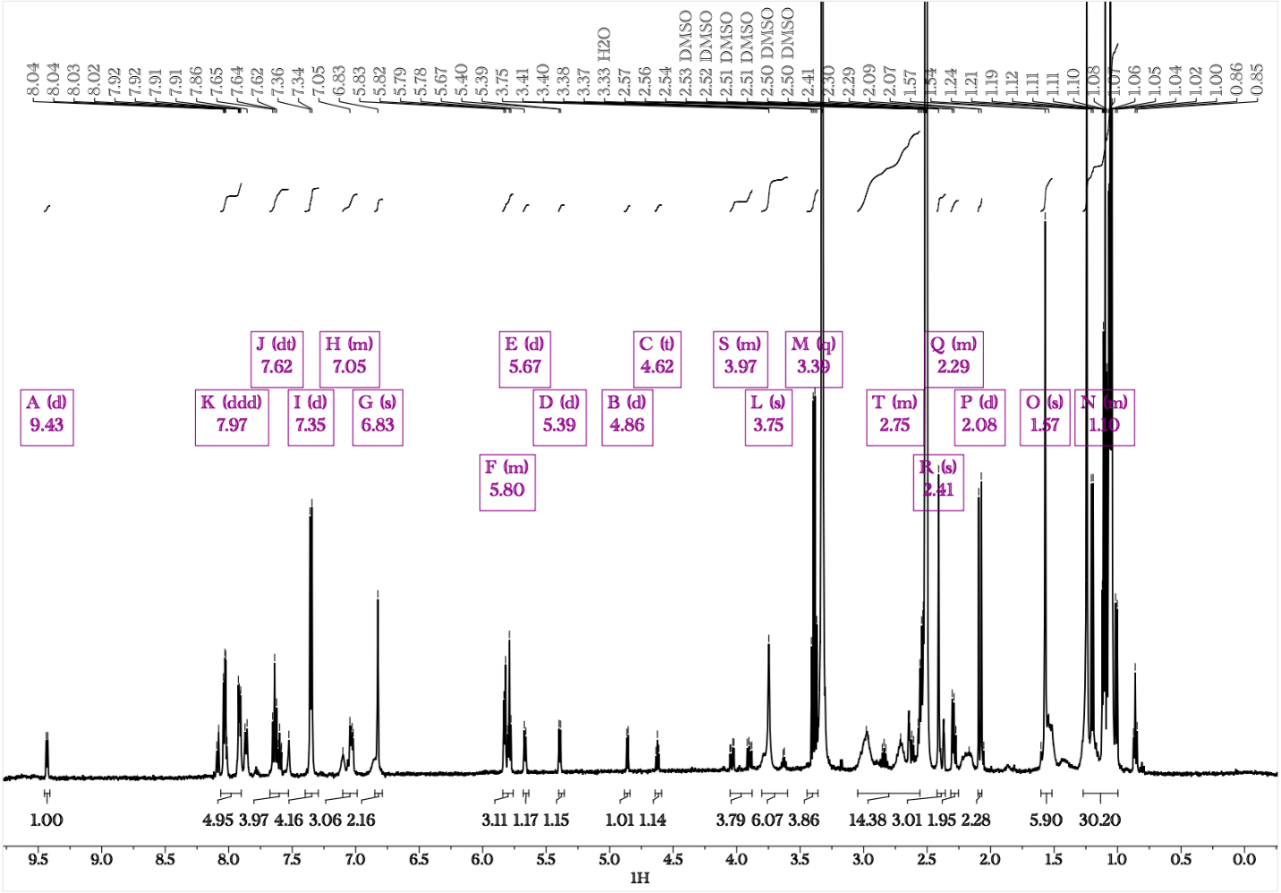


**Figure S26 ^1^H NMR spectrum of complex 5 in DMSO-*d*_6_**


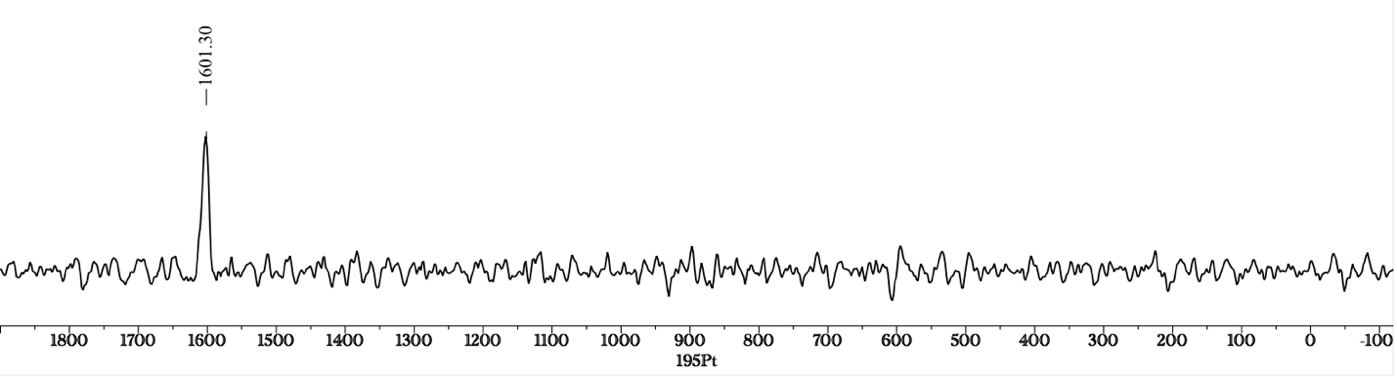


**Figure S27 ^195^Pt NMR spectrum of complex 5 in DMSO**


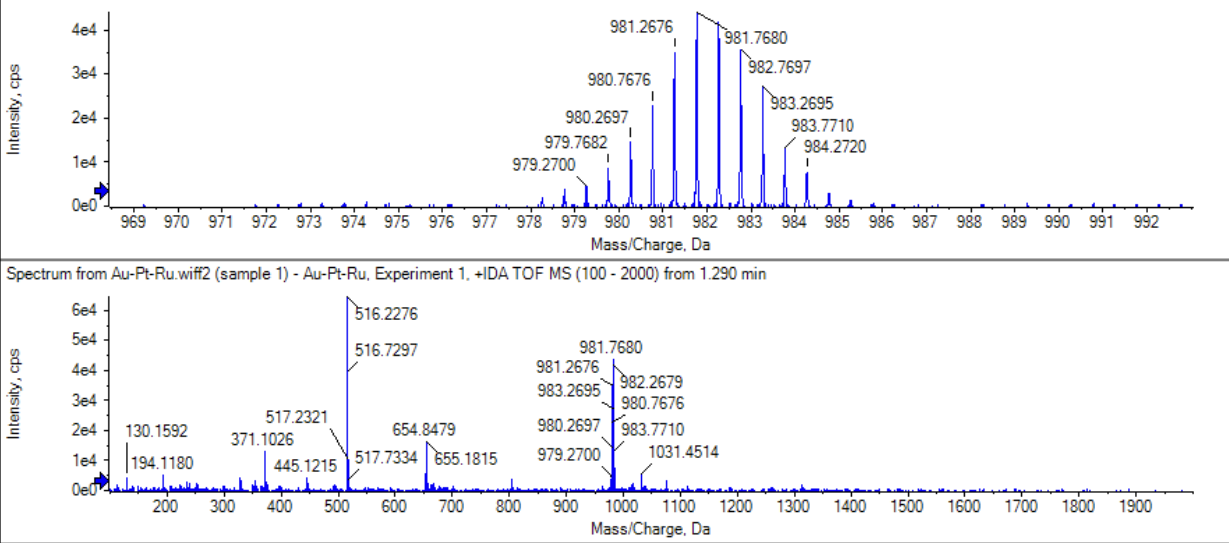

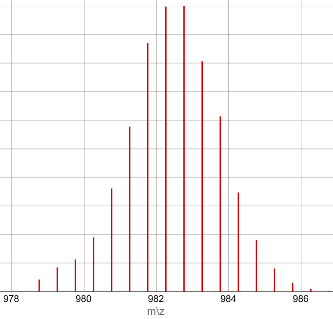


Calculated

**Figure S28 ESI-HRMS (+ve) of complex 5**


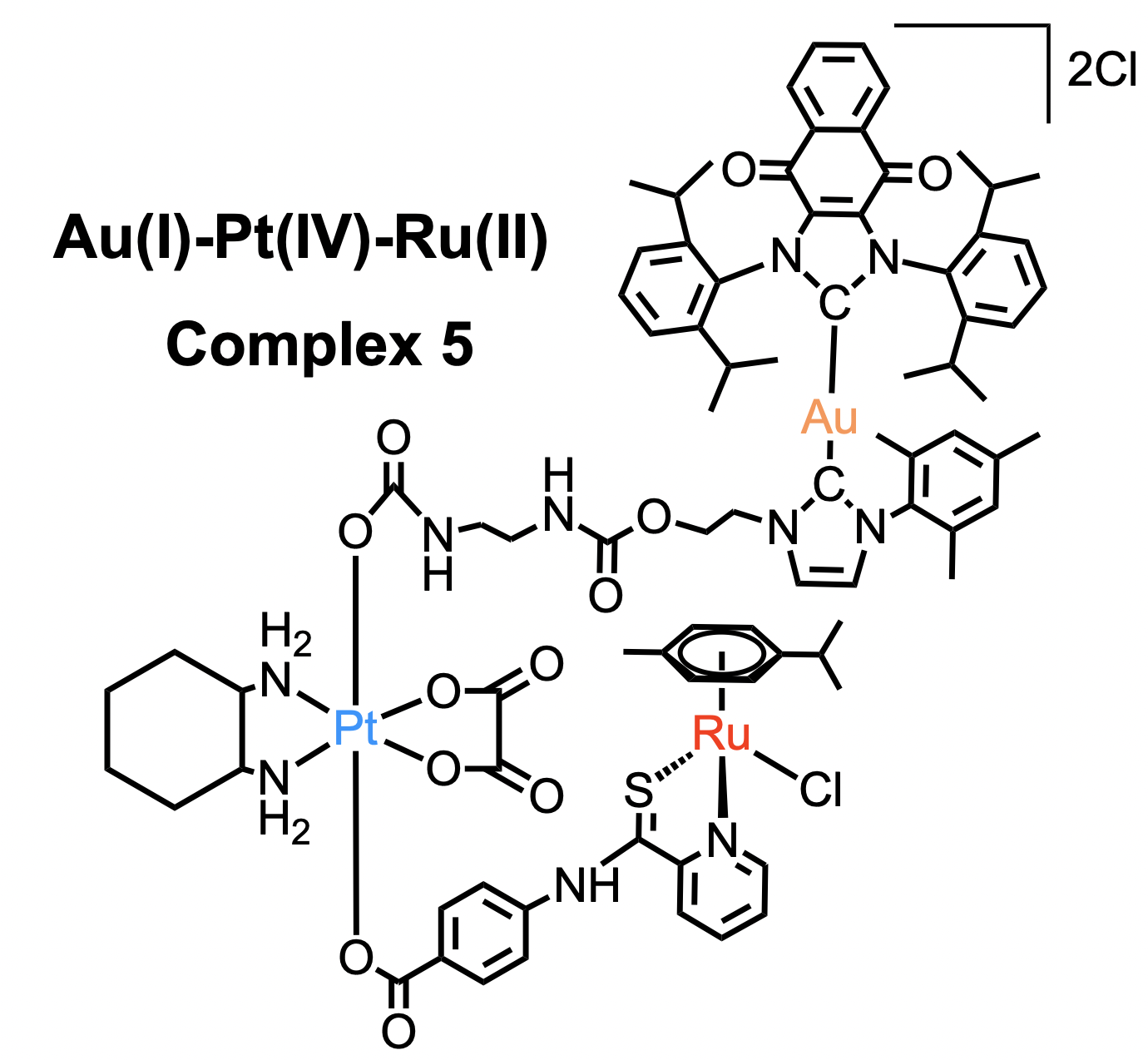

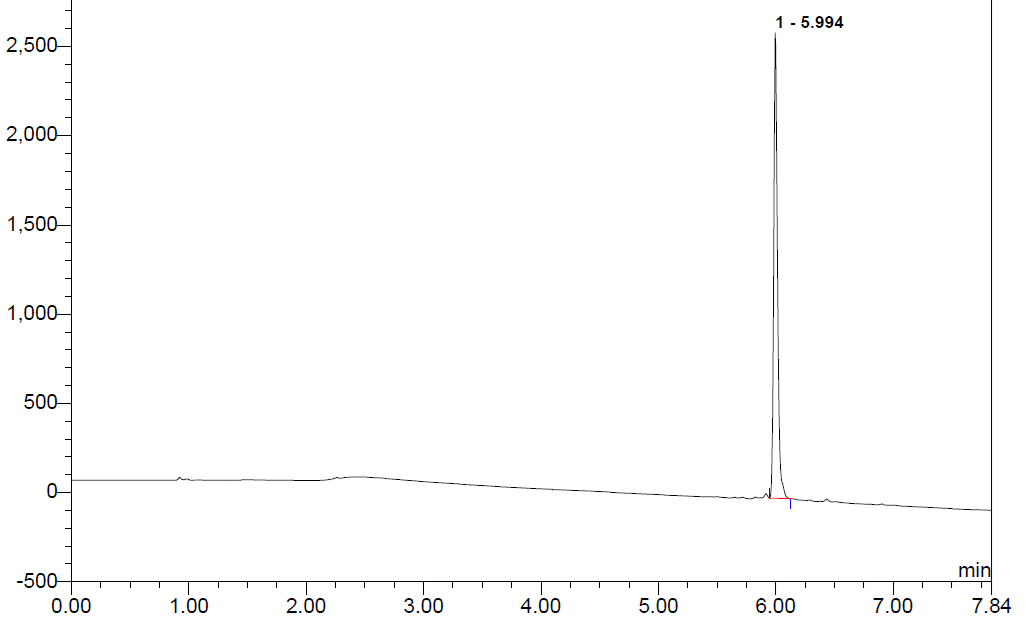


**Figure S29 HPLC chromatogram of complex 5 -** 0-100% acetonitrile in 5.84 min + 2 min constant 100% acetonitrile.


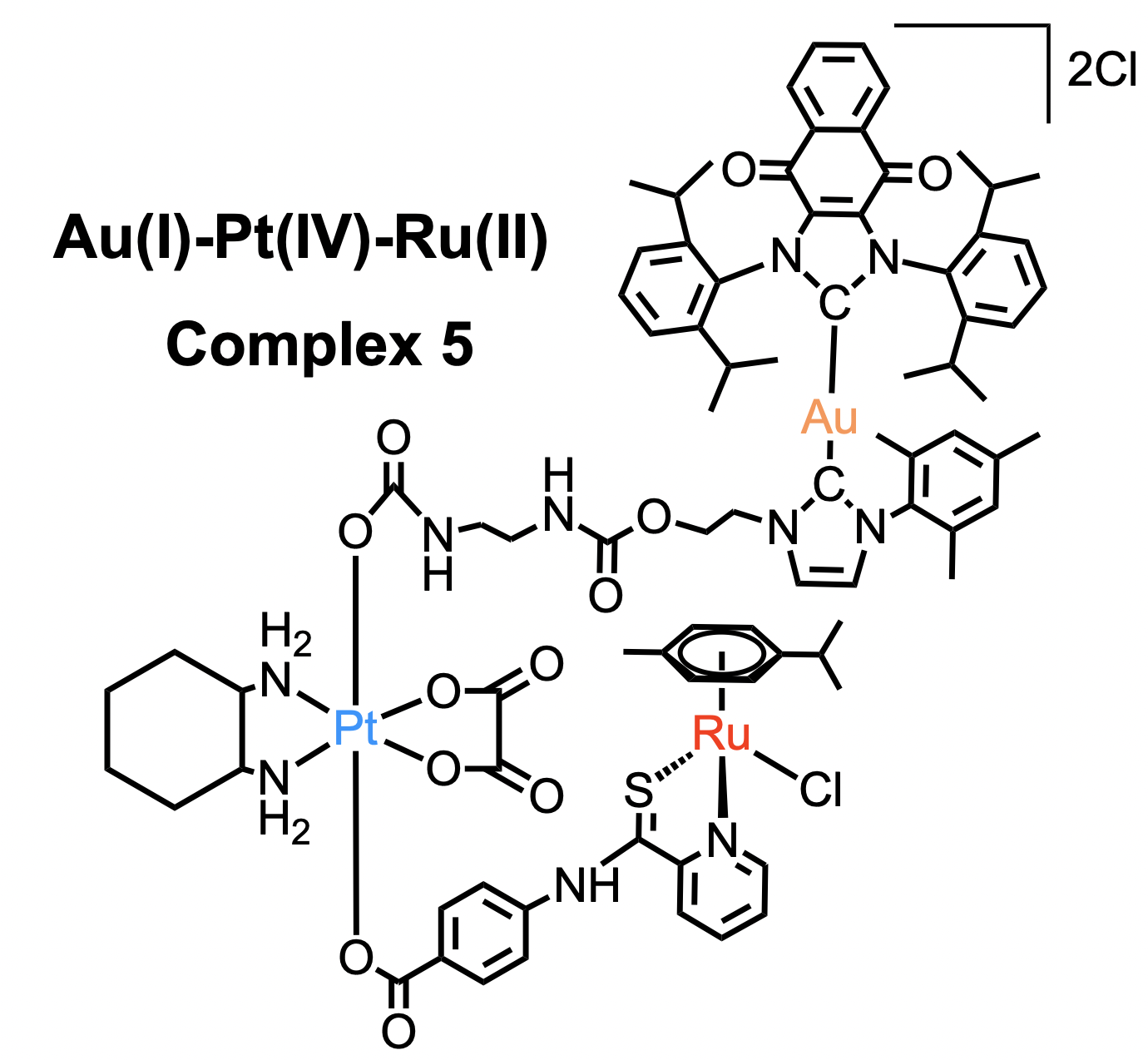


**Figure S30 Reduction and stability –** Half-life of bi- & tri-metallic complexes determined by HPLC.

**Figure S31 Complex 5 reduction products –** Reduction of triple metal complex 5 incubated with 10 equiv. Asc. at pH 7 and 37 ºC as monitored via LC-MS.

**Figure S32 Ruthenium dimer formation as inferred from an LC-HRMS analysis –** Plecstatin-1 dissolved in water or complex **5** reduction mixture after 4 h incubation at 37 ºC.

**Figure S33 Lipoate reduction assay –** HCT-116 cells were incubated with the indicated complexes at 1 µM concentrations for 24 h.


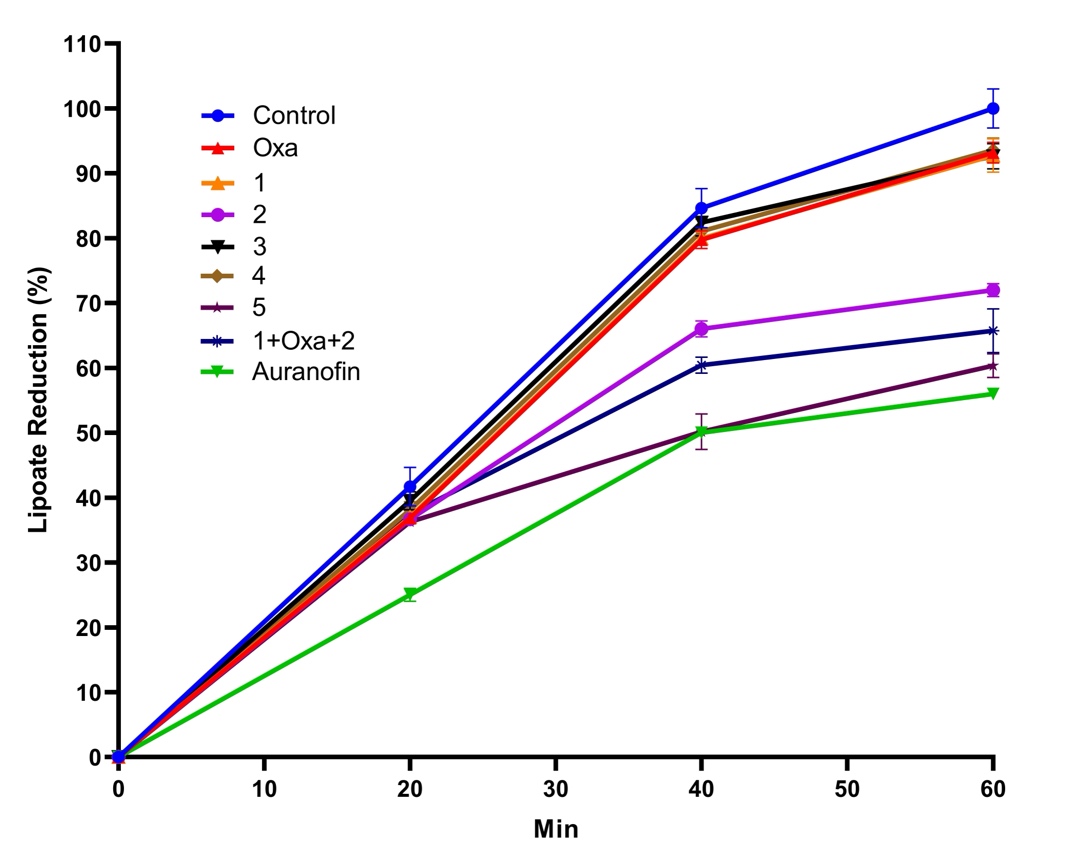


**Figure S34 Confocal microscopic studies of ROS generation after a 24 h incubation period at the IC_50_ concentrations -** CM-H_2_DCFDA dye was used as general oxidative stress marker.

**Figure S35 Annexin-V / PI FACS Analysis –** CT26 cells incubated with the indicated complexes at their respective at their IC_50_ concentrations for 24 h.

**Figure S36 CRT Translocation FACS analysis –** CT26 cells incubated with the indicated complexes at their respective IC_50_ concentrations for 24 h.


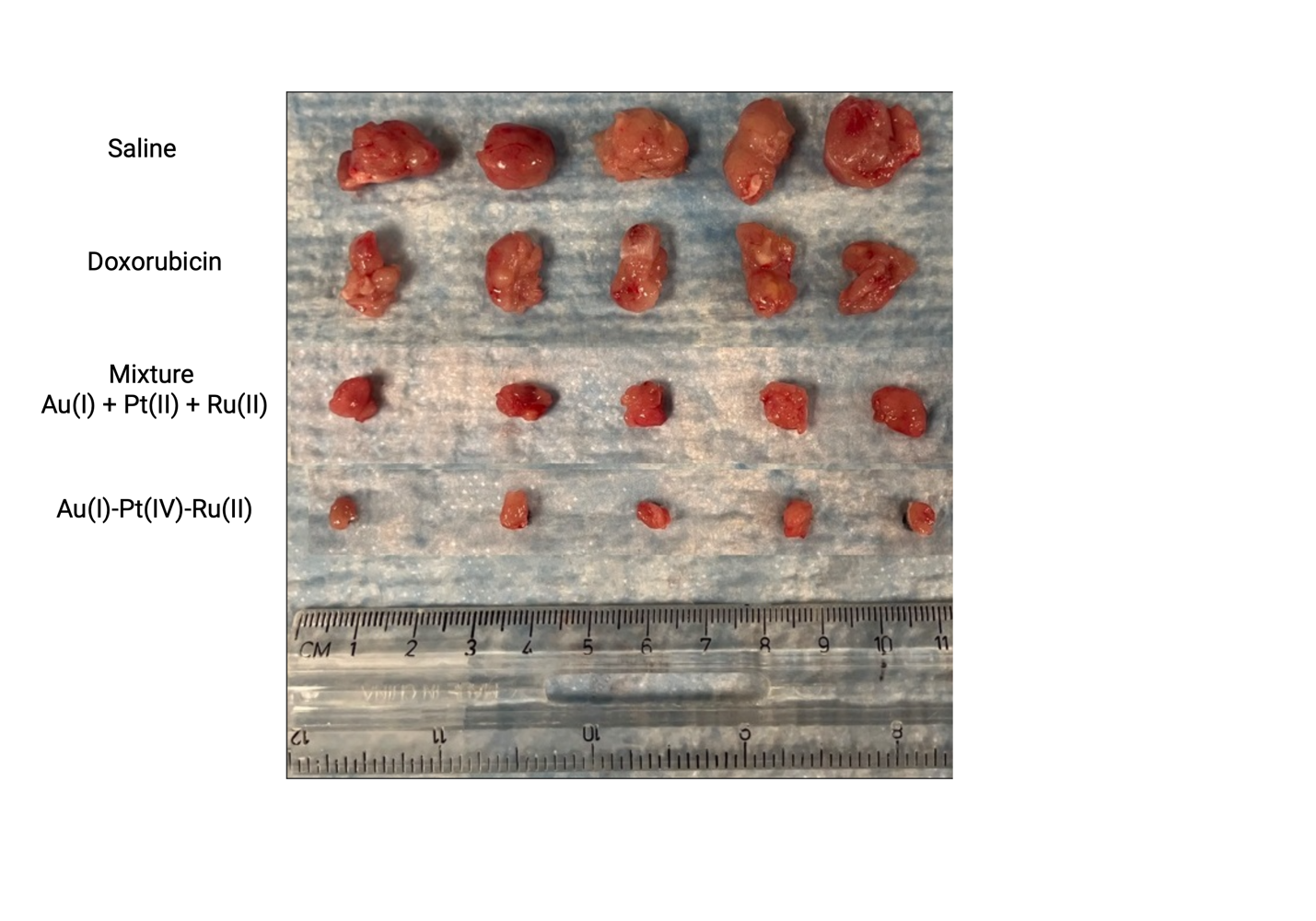


**Figure S37 Comparison of the tumor size –** Mice were euthanized on day 16 and tumors were resected.

**Figure S38 Example of WBC differential manual count –** 200 WBC were counted and classified by preparing blood smear samples of tail blood stained with Wright solution.

# References

(1) S. M. Meier, M. Hanif, Z. Adhireksan, V. Pichler, M. Novak, E. Jirkovsky, M. A. Jakupec, V. B. Arion, C. A. Davey, B. K. Keppler, C. G. Hartinger, *Chem. Sci.* **2013**, *4*, 1837–1846.

(2) S. Sen, S. Hufnagel, E. Y. Maier, I. Aguilar, J. Selvakumar, J. E. DeVore, V. M. Lynch, K. Arumugam, Z. Cui, J. L. Sessler, J. F. Arambula, *J. Am. Chem. Soc.* **2020**, *142*, 20536–20541.

(3) T. Babu, A. Sarkar, S. Karmakar, C. Schmidt, D. Gibson, *Inorg. Chem.* **2020**, *59*, 5182–5193.

(4) D. Cirri, S. Pillozzi, C. Gabbiani, J. Tricomi, G. Bartoli, M. Stefanini, E. Michelucci, A. Arcangeli, L. Messori, T. Marzo, *Dalton Trans.* **2017**, *46*, 3311–3317.

(5) S. Sen, Y. Li, V. Lynch, K. Arumugam, J. L. Sessler, J. F. Arambula, *Chem. Commun.* **2019**, *55*, 10627–10630.

(6) T.-C. Chou, P. Talalay, *Advances in Enzyme Regulation* **1984**, *22*, 27–55.

(7) B. dos Santos, M. C. Bion, M. Goujon-Svrzic, P. Maher, A. L. Dafre, *Analytical Biochemistry* **2024**, *687*, 115445.

(8) P. Liu, L. Zhao, O. Kepp, G. Kroemer, in *Methods in Enzymology* (Eds.: L. Galluzzi, N.-P. Rudqvist), Academic Press, **2020**, pp. 1–13.

(9) M. Bjorner, L. Zhu, *Pathobiol Aging Age Relat Dis* **2019**, *9*, 1647400.
